# Ruxolitinib exposure in patients with acute and chronic graft versus host disease in routine clinical practice—a prospective single-center trial

**DOI:** 10.1007/s00280-021-04351-w

**Published:** 2021-09-10

**Authors:** Nora Isberner, Sabrina Kraus, Götz Ulrich Grigoleit, Fatemeh Aghai, Max Kurlbaum, Sebastian Zimmermann, Hartwig Klinker, Oliver Scherf-Clavel

**Affiliations:** 1 Department of Internal Medicine II, University Hospital Würzburg, Oberdürrbacher Strasse 6, 97080 Würzburg, Germany; 2 Present Address: Department of Hematology, Oncology and Immunology, Helios Hospital Duisburg, Duisburg, Germany; 3 Department of Internal Medicine I and Core Unit Clinical Mass Spectrometry, Division of Endocrinology and Diabetology, University Hospital Würzburg, Würzburg, Germany; 4 Institute of Pharmacy and Food Chemistry, University of Würzburg, Würzburg, Germany

**Keywords:** Ruxolitinib, Graft versus host disease, Therapeutic drug monitoring, CYP3A4, CYP2C9, Toxicity

## Abstract

**Purpose:**

Knowledge on Ruxolitinib exposure in patients with graft versus host disease (GvHD) is scarce. The purpose of this prospective study was to analyze Ruxolitinib concentrations of GvHD patients and to investigate effects of CYP3A4 and CYP2C9 inhibitors and other covariates as well as concentration-dependent effects.

**Methods:**

262 blood samples of 29 patients with acute or chronic GvHD who were administered Ruxolitinib during clinical routine were analyzed. A population pharmacokinetic model obtained from myelofibrosis patients was adapted to our population and was used to identify relevant pharmacokinetic properties and covariates on drug exposure. Relationships between Ruxolitinib exposure and adverse events were assessed.

**Results:**

Median of individual mean trough serum concentrations was 39.9 ng/mL at 10 mg twice daily (IQR 27.1 ng/mL, range 5.6–99.8 ng/mL). Applying a population pharmacokinetic model revealed that concentrations in our cohort were significantly higher compared to myelofibrosis patients receiving the same daily dose (*p* < 0.001). Increased Ruxolitinib exposure was caused by a significant reduction in Ruxolitinib clearance by approximately 50%. Additional comedication with at least one strong CYP3A4 or CYP2C9 inhibitor led to a further reduction by 15% (*p* < 0.05). No other covariate affected pharmacokinetics significantly. Mean trough concentrations of patients requiring dose reduction related to adverse events were significantly elevated (*p* < 0.05).

**Conclusion:**

Ruxolitinib exposure is increased in GvHD patients in comparison to myelofibrosis patients due to reduced clearance and comedication with CYP3A4 or CYP2C9 inhibitors. Elevated Ruxolitinib trough concentrations might be a surrogate for toxicity.

**Supplementary Information:**

The online version contains supplementary material available at 10.1007/s00280-021-04351-w.

## Introduction

Graft versus host disease (GvHD) remains to be one of the major causes for morbidity and mortality after allogeneic hematopoietic stem cell transplantation (allo-HSCT) with 30–60% of recipients developing acute GvHD (aGvHD) and 30–70% developing chronic GvHD (cGvHD) [1–4]. The backbone of first-line therapy for moderate and severe GvHD still are systemic high-dose corticosteroids. Yet, treatment with corticosteroids comes along with severe toxicities while ultimately 40% of patients with aGvHD and 50–60% of patients with cGvHD do not show sustained responses [5–8]. Treatment for both acute and chronic steroid-refractory GvHD (SR-GvHD) comprise variable immunosuppressants, most of which are not approved for GvHD. Numerous clinical trials have been carried out without leading to evidence-based consensus regarding the ideal management of SR-GvHD [9–11].

In May 2019, Ruxolitinib was approved by the US Food and Drug administration (FDA) for treatment of SR-aGvHD based on the REACH1 trial [12]. Significant improvements in overall response, failure-free and overall survival compared to investigator’s choice were demonstrated by the REACH2 trial [13]. Ruxolitinib is not yet approved for treatment of SR-cGvHD, but primary findings of the REACH3 trial are promising with significantly greater overall response rate and improvements in failure-free survival and patient-reported symptoms in patients receiving Ruxolitinib compared to best available therapy [14].

Ruxolitinib is an orally administered small molecule multi-kinase inhibitor with potent and selective inhibition activity against Janus Kinase (JAK) 1/2 [15]. It is metabolized by hepatic enzymes of the cytochrome P450 (CYP) family, predominantly by CYP3A4 and to a lesser extent by CYP2C9 [16]. Comedication with perpetrators of these enzymes may lead to varying Ruxolitinib exposure. Especially coadministration of azoles, which are frequently used for antifungal prophylaxis, is important to note as all azoles are potent inhibitors of CYP3A4 and some additionally of CYP2C9. At present, dose reduction in GvHD patients is only recommended for coadministration of Ketoconazole [16] even though strong inhibitory potential has been demonstrated by other azoles as well [17, 18]. Genetic polymorphisms, epigenetic influences as well as other factors such as sex or age may additionally influence CYP metabolism and consequently Ruxolitinib concentrations [19]. Drug disposition may further be affected by concurrent use of proton pump inhibitors (PPI) due to pH-dependent solubility of Ruxolitinib [20, 21]. Moreover, hepatic or renal impairment as well as gastrointestinal or liver GvHD are common conditions after allo-HSCT [22–25] potentially influencing Ruxolitinib exposure.

Even though polypharmacy and organ impairment are frequent in allo-HSCT recipients, there are no data on Ruxolitinib exposure in clinical routine in patients with GvHD. Therefore, we conducted a prospective single-center clinical trial analyzing Ruxolitinib concentrations in patients treated for aGvHD or cGvHD of various organs in a routine clinical setting and evaluated Ruxolitinib exposure and effects of comedication and patient characteristics using a population pharmacokinetic (popPK) model and assessed concentration-dependent toxicities.

## Materials and methods

### Study design and patient selection

Patients were enrolled in a noninterventional prospective clinical trial at the University Hospital of Würzburg between February 2019 and February 2021. The study was approved by the Ethics Commission of the University of Würzburg (ref 199/18-am). All performed procedures were in accordance with the Declaration of Helsinki. Written informed consent was obtained from all patients. Patients were eligible for enrollment if they received Ruxolitinib at any dosing regimen for aGvHD or cGvHD of any organ after having undergone allo-HSCT and if they were ≥ 18 years of age. After enrollment blood samples and patient data were collected regularly during routine outpatient visits for 1 year. Treatment was managed at the discretion of the treating physician.

### Assessment of adverse events and collection of patient data

Adverse events (AE) were registered by questioning patients at each sampling time point. Patients were specifically asked for AE occurring in ≥ 1/100 patients according to the prescribing information [16]. Clinical AE were graded according to the Clinical Terminology for Adverse Events Criteria (CTCAE) Version 5.0 [26]. Further recorded patient data included age, sex, height, weight, ethnicity, smoking status, dosing regimen, and comedication. Relevant laboratory parameters were recorded in parallel to blood sampling (estimated glomerular filtration rate (eGFR), serum creatinine, serum albumin, total cholesterol, total bilirubin, hemoglobin, white blood cell count, absolute neutrophil count, platelet count, aspartate, and alanine aminotransferase).

### Sample collection

Serum samples were collected at each outpatient visit during routine blood withdrawal. Patients were asked to take Ruxolitinib after blood withdrawal. Date and time of last administration and blood withdrawal were recorded. Samples obtained at least 48 h after initiation or modification of therapy were considered steady state samples [27]. Samples obtained between eight and 30 h after last administration (depending on dosing scheme) were considered trough levels [16].

### Quantification of Ruxolitinib serum levels

Ruxolitinib concentrations were determined using a fully validated liquid chromatography-tandem mass spectrometry method (LC–MS/MS) [28]. In brief, after protein precipitation with acetonitrile separation was achieved on a Waters XBridge Phenyl 3.5 µm (2.1 × 50 mm) column using gradient elution (flow rate 400 μL/min). Ruxolitinib was quantified using ^2^H_4_-Ruxolitinib as internal standard via multiple reaction monitoring analysis using positive electrospray ionization. Lower level of quantification (LLOQ) of the method was 2 ng/mL. Concentrations < LLOQ were excluded from further statistical analysis but included as censored data (between 0 ng/mL and LLOQ) in the popPK analysis.

### Data processing and statistical analysis

Data were collected and processed using Microsoft Excel 2016 Version 16.0 (Microsoft Corporation, Redmond, WA, USA). Statistical calculations and visualization of results were performed with R Studio Version 1.2.5042 (RStudio Incorporation, Boston, MA, USA) and running R version 4.0.5 (R Foundation for Statistical Computing, Vienna, Austria, 2020). To reduce bias caused by the unbalanced number of samples per patient, all Ruxolitinib trough serum concentrations of the same patient at the same dosage were summarized into an individual mean trough serum concentration for descriptive statistical analysis. To fully display the degree of variability, concentrations were also summarized across all patients, stratified by dosing scheme. Pairwise comparisons were performed using Wilcoxon rank-sum test. Wilcoxon-signed-rank test was used for unpaired samples. For comparisons between more than two groups the Kruskal–Wallis one-way analysis of variance combined with Dunn’s test post hoc was used. Spearman’s correlation was used for analysis of AE. Logistic regression followed by receiver operator characteristic (ROC) analysis was used to evaluate the risk for the occurrence of AE of any grade or type on a visit in relation to the corresponding trough concentration on that visit. Due to the explorative nature of the study, *p* values obtained from multiple comparisons were not corrected for multiple testing. A *p* value < 0.05 was considered statistically significant. A Spearman’s rho (ρ) correlation was defined as follows: 0 to 0.3 or 0 to −0.3, weak; 0.3 to 0.7 or −0.3 to −0.7, moderate and 0.7 to 1 or −0.7 to −1, strong.

### Exploratory popPK analysis

An exploratory popPK analysis was conducted to identify relevant covariates influencing drug exposure, to predict the influence of strong CYP3A4 or CYP2C9 inhibitors or PPI and to generate population estimates for apparent oral clearance (CL/*F*) and apparent central volume of distribution (*V*_c_/F) as classical statistical analysis was not suitable due to the unbalanced number of samples per patient. The analysis was conducted on the individual raw data (as opposed to the descriptive statistics) using Monolix 2020R1 (Antony, France: Lixoft SAS, 2020) and was based on a previously published popPK model by Chen et al. [29] investigating pharmacokinetics in patients with primary and secondary myelofibrosis (MF).

Oral pharmacokinetics were described by a two-compartment model using first order absorption with lag time and linear elimination from the first compartment only. The population mean values for CL/*F* and *V*_c_/F were re-estimated using the SAEM algorithm implemented in Monolix. The remaining parameters, except for the covariate model, were fixed to the original values. As in the original model, all pharmacokinetic parameters were assumed to be log-normally distributed. Dichotomous categorical covariates, were implemented as follows:

1 $$\mathrm{log}{\mathrm{CL}/F}_{\mathrm{typical}}={\mathrm{logCL}/F}_{\mathrm{pop}}+{\beta }^{\mathrm{COV}}$$

with COV being 0 or 1 encoding for the presence or absence of covariate effect.

Continuous covariates were scaled to the population mean values using the following exponential relationship:

2 $$\mathrm{log}{\mathrm{CL}/F}_{\mathrm{typical}}={\mathrm{logCL}/F}_{\mathrm{pop}}+\beta \bullet \mathrm{COV}$$

with COV being the value of individual covariate, e.g., scaled eGFR = eGFR/70.2.

Inter-individual variability (*η*) was implemented for CL/*F* and *V*_c_/*F* according to:

3 $$\begin{gathered} \log{{{\text{CL}}}}/{{F_{{{\text{ind}}}} }} = \log {{{\text{CL}}}}/{{F_{{{\text{typical}}}} }} + \eta _{{{\text{ind}}, {{{\text{CL}}}}{F}}} \;{\text{with}}\;\eta _{{{{{\text{CL}}}}/{F}}} \sim N\left( {0,\omega _{{{{{\text{CL}}}}/{F}}} ^{2} } \right) \hfill \\ \log V_{c} /F_{{{\text{ind}}}} = \log V_{c} /F_{{{\text{typical}}}} + \eta _{{{\text{ind}},~Vc/F}} \;{\text{with}}\;\eta _{{Vc/F}} \sim N\left( {0,\omega _{{Vc/F}} ^{2} } \right) \hfill \\ \end{gathered}$$

Residual unexplained variability (RUV) was modeled using a proportional error model represented by the parameter b and the following equation:

4 $${\mathrm{Conc}}_{\mathrm{obs}}={\mathrm{Conc}}_{\mathrm{pred}} \bullet \left(1+ \epsilon \right)\mathrm{ with }\epsilon \sim N(0,{b}^{2})$$

Comedication was modeled as dichotomous categorical covariate (patient does/does not receive specific comedication) leading to the covariate model. Inter-individual variability was modeled for CL/*F* and *V*_c_/*F* to study the effect of comedication and patient characteristics on Ruxolitinib pharmacokinetics leading to the base model which was used as a starting point for the development of the covariate model. A stepwise approach was used to develop the covariate model. Covariate effects were assessed using the Wald test and by comparing objective function values (OFV, which is the −2-log-likelihood) and derived descriptors such as Bayesian Information Criteria (BIC), corrected Bayesian Information Criteria (cBIC), and Akaike Information Criteria (AIC). The criterion for forward inclusion was an improvement of the OFV (*p* ≤ 0.1). In the backward elimination, a limit for the *p* value of < 0.05 was applied. The following covariates were tested: the presence of strong or moderate CYP3A4 or CYP2C9 inhibitors on CL/*F*, the presence of PPI on *F* (as relative bioavailability), aspartate aminotransferase (AST) on CL/*F*, total body weight on *V*_c_/*F*, eGFR on CL/*F*, sex on CL/*F*, and smoking habit on CL/*F*.

The model published by Chen et al. was also encoded in R using the mrgsolve package for R [30]. Applying patient covariates and dosing schemes, the model was used to predict every observed concentration of every patient included in our study based on individual patient covariates and dosing data. Additionally, steady state concentrations of 1000 virtual typical patients receiving 10 mg Ruxolitinib twice daily were simulated with random effects using 1000 virtual patients derived from patient characteristics of our population (Table 1) to generate a typical serum concentration–time profile including 95% prediction intervals in steady state.

**Table 1:** Baseline patient demography

Patient characteristic	No. of patients	%
Total	29	
Age, median (range), (IQR)	53 (22–78), (18)	
Weight (kg), mean (range)	74.1 (46–111)	
Height (cm), mean (range)	174 (156–196)	
BMI (kg/m^2^), median (range), (IQR)	23.8 (16.8–43.4), (6.3)	
Gender		
Male	17	58.6
Female	12	41.4
Ethnicity		
Caucasian	29	100
Smoking status		
Smoker	2	6.9
Non-smoker	27	93.1
CYP inhibitors^a^		
Strong CYP3A4 or CYP2C9 inhibitor		
≥ 1 inhibitor	24	82.8
None	5	17.2
Moderate or strong CYP3A4 or CYP2C9 inhibitor		
1 inhibitor	25	86.2
≥ 2 inhibitors	1	3.4
None	3	10.3
Proton pump inhibitor		
Yes	21	72.4
No	8	27.6
Diagnosis^b^		
Acute GvHD	3	10.3
Skin	3	10.3
Gut	1	3.5
Liver	1	3.5
Chronic GvHD	26	89.7
Skin	22	75.9
Oral	9	31.0
Ocular	11	37.9
Gut	8	27.6
Esophagus/stomach	1	3.5
Liver	9	31.0
Lung	3	10.3
Joints	2	6.9
Pericardial/pleural effusions	1	3.5
Duration of treatment at enrollment (days), median (range)	76 (3–1425)	

^a^Refers to administration of CYP inhibitors at inclusion

^b^Refers to diagnosis and organ involvement at initiation of Ruxolitinib therapy

## Results

### Patient and sample characteristics

In total, 30 patients were included in the study. One patient was excluded from further analysis due to self-reported non-adherence. Consequently, 262 Ruxolitinib concentrations (including 182 trough concentrations) of 29 patients with various underlying diseases were analyzed. All samples were obtained in steady state. The median number of samples per patient was 8 (IQR: 12, range: 1–23). 66.8% of samples were collected under a daily dose of 20 mg. Other observed daily doses were 5, 10, 15, 30, and 40 mg (1.53%, 15.3%, 11.8%, 0.76%, and 0.76% of all samples). No patient had signs of ongoing gastrointestinal or liver GvHD > stage 1 at the time of sample collection. Details on patient demography and sample characteristics are presented in Table 1 and Supplementary Table 1, baseline laboratory characteristics at inclusion can be found in Supplementary Table 2.

### Observed Ruxolitinib serum concentrations

**Fig. 1:**
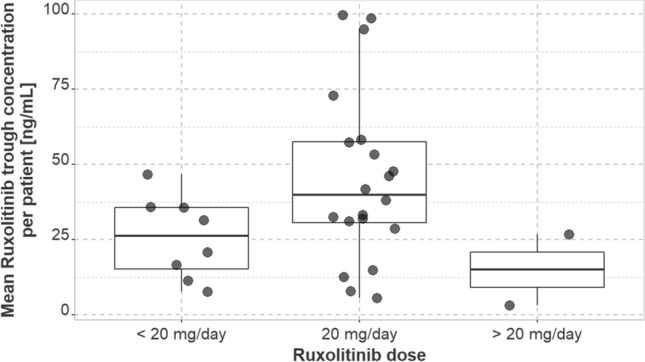
Median of observed individual mean trough serum concentrations of Ruxolitinib stratified by daily Ruxolitinib dose; patients who experienced dose adjustment might occur multiple times

Median Ruxolitinib trough serum concentrations of all samples across all individuals are presented in Table 2. Median of individual mean trough serum concentrations was 39.9 ng/mL at 20 mg daily (IQR, 27.1 ng/mL; range, 5.6–99.8 ng/mL, *n* = 20 individuals), 15 ng/mL at > 20–40 mg daily (IQR, 11.8 ng/mL, range, 3.2–26.8 ng/mL, *n* = 2 individuals), and 26.2 ng/mL at 5–< 20 mg daily (IQR, 20.4 ng/mL; range, 7.7–46.8 ng/mL, *n* = 8 individuals) (Fig. 1).

**Table 2:** Steady state Ruxolitinib trough serum concentrations of all samples across all individuals

Daily dose (mg)	No. of samples (no. of excluded samples < LLOQ)	Median (ng/mL)	IQR (ng/mL)	Range (ng/mL)
All	174 (8)	31.4	31.95	2.2–229
20	120 (2)	40.5	33.9	2.7–229
> 20–40	3 (1)	25.1	12.1	4.3–28.5
5–< 20	51 (5)^a^	17.5	10.9	2.2–155

*LLOQ* lower limit of quantification (2 ng/mL)

^a^All trough serum concentrations at 5 mg daily were ≤ 2.17 ng/mL (3 of 4 samples < LLOQ)

**Fig. 2:**
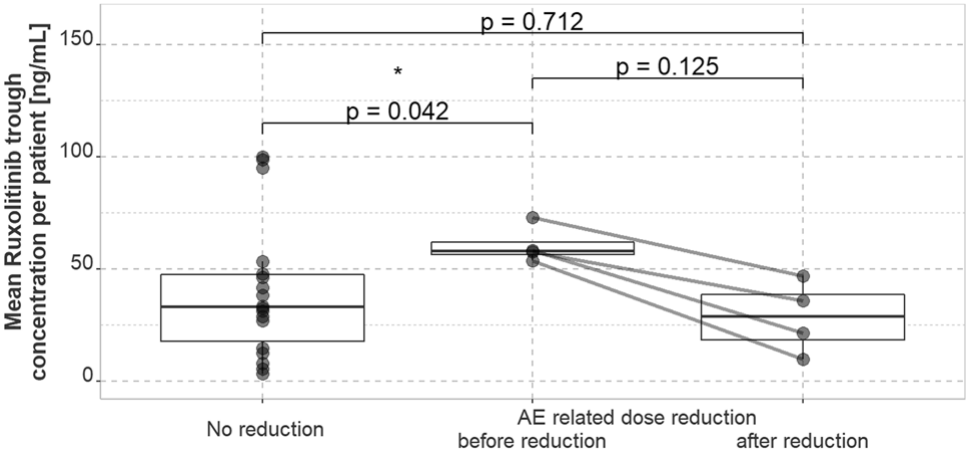
Mean trough serum concentrations of Ruxolitinib of patients receiving 10 mg twice daily in patients without AE-related dose reduction (*n* = 18) compared to patients with AE-related dose reduction (*n* = 4) before and after reduction. *AE* adverse event

Patients not requiring AE-related dose reduction at a daily dose of 20 mg had a median of individual mean trough serum concentrations of 39.8 ng/mL (IQR, 29.6 ng/mL; *n* = 18 individuals). Patients who had dose reductions for AE at the same daily dose had a significantly higher median of individual mean trough serum concentrations of 60.6 ng/mL (IQR, 5.39 ng/mL; *n* = 4 individuals) (*p* = 0.042). After dose reduction, median of mean trough concentrations was 28.4 ng/mL (IQR, 20.1 ng/mL) in these patients and did no longer differ significantly from concentrations of patients receiving 20 mg daily without need for AE-associated dose reduction (*p* = 0.712) (Fig. 2).

### Simulated Ruxolitinib serum concentrations

**Fig. 3:**
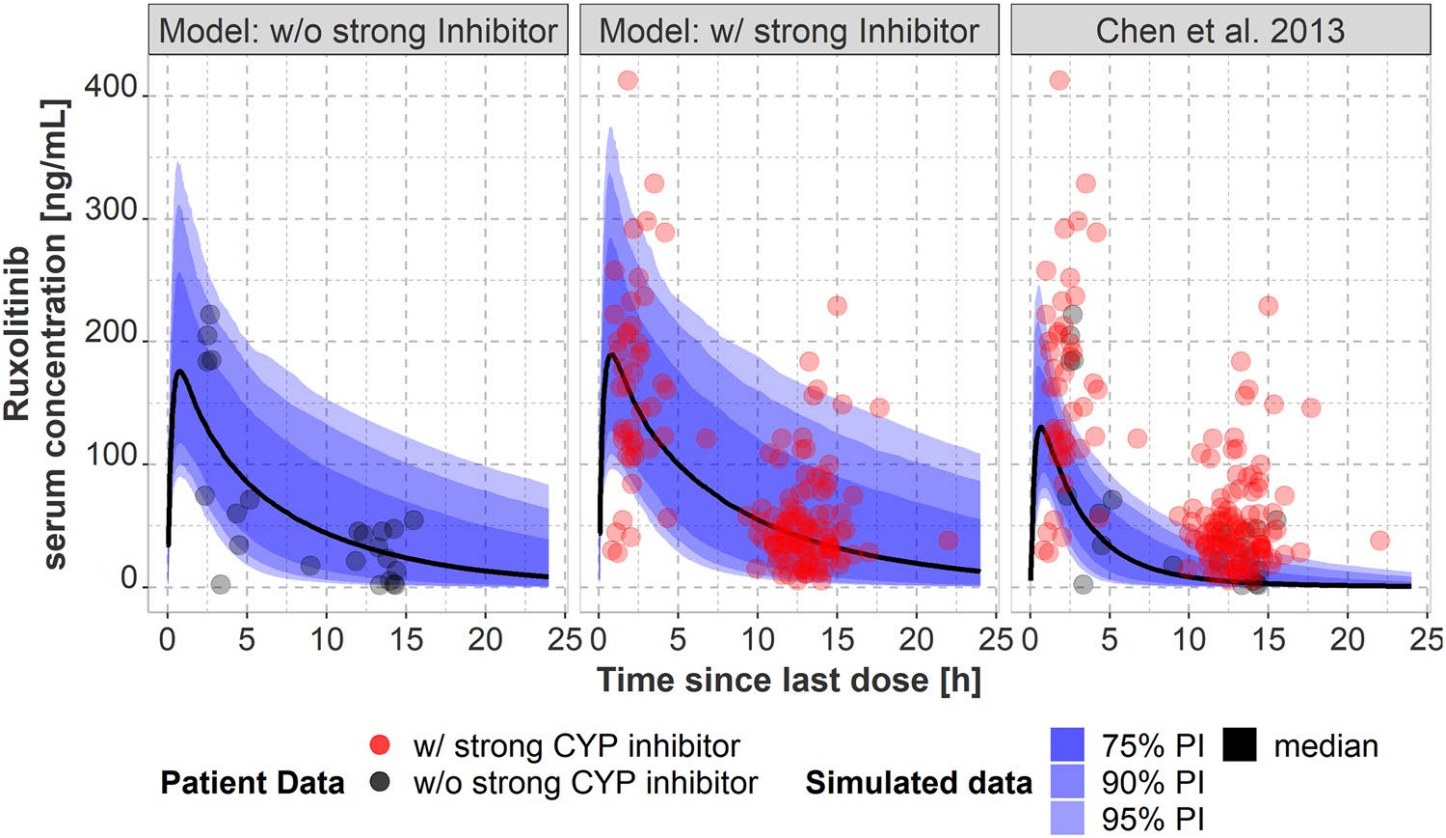
Comparison between observed Ruxolitinib concentrations at 10 mg twice daily in patients (dots) and simulated (popPK model predicted) concentrations using a simulated serum concentration–time profile including median, 75%, 90%, and 95% prediction interval in steady state for 1000 typical patients of our population receiving 10 mg Ruxolitinib twice daily. Predictions were performed with either our modified model for patients without (**a**) and with (**b**) comedication with strong CYP 3A4 or CYP2C9 inhibitors or with Chen et al.’s previously published popPK model (**c**) obtained in myelofibrosis patients using 1000 virtual patients sampled from our GvHD population receiving a dose of 10 mg twice daily. *popPK* population pharmacokinetic

Chen et al.’s popPK model [29] for patients with MF was used to predict every observed concentration of all patients included in our study using covariates without random effects and the recorded dosing information. Simulated Ruxolitinib concentrations were significantly lower than observed real-life concentrations in our study (*p* < 0.001). The median paired differences for Ruxolitinib trough and non-trough serum concentrations were 27.1 ng/mL (IQR 32.4 ng/mL) and 64.9 ng/mL (IQR 96.4 ng/mL), respectively. Using Chen et al.’s popPK model for simulating 1000 typical patients taking a dose of 10 mg twice daily with random effects based on the mean patient characteristics of our population corroborates this finding, since a great part of Ruxolitinib trough concentrations of patients in our cohort receiving 10 mg twice daily was not contained within the 95% prediction interval and the mean trough concentration was underpredicted (Fig. 3c). Using our modified model for GvHD patients with and without comedication with relevant strong CYP inhibitors proved to be suitable as the majority of concentrations was within the 75% prediction interval (Fig. 3a, b).

### Ruxolitinib clearance

Generated population estimates for CL/*F* and *V*_c_/*F* for our GvHD population revealed that Ruxolitinib clearance is reduced by approximately 50% in comparison to the population investigated by Chen et al. (patients with MF). The typical CL/F obtained in our study was 9.74 L/h whereas Chen et al. found values of 22.1 L/h for a typical male and 17.7 L/h for a typical female, respectively. We did not observe an impact of patient sex and bodyweight on clearance and volume of distribution as described by Chen et al. after evaluation of the base model (Supplementary Table 3).

### Investigation of covariates on Ruxolitinib exposure

Comedication (Supplementary Table 4), patient characteristics and laboratory parameters were used in the covariate model to investigate the influence of covariates on inter-individual variability on clearance, central volume of distribution and relative bioavailability. Comedication with at least one strong CYP3A4 or CYP2C9 inhibitor was the only significant covariate on Ruxolitinib CL/F (OFV improvement: *p* = 0.029, Wald test: *p* = 0.003). Simultaneous administration of PPI did not affect Ruxolitinib exposure, neither did age, sex, AST, body weight, smoking habit, or eGFR. The effect of strong CYP3A4 or CYP2C9 inhibitors on Ruxolitinib clearance (β_CL/F,STRONG_INH_) explained about 4% of inter-individual variability on CL/F (Supplementary Table 3). Back transformation of the covariate effect (β_CL/F,STRONG_INH_ = −0.16) showed that CL/F was reduced by 15% due to coadministration of at least one strong CYP3A4 or CYP2C9 inhibitor compared to patients of our study population not receiving relevant strong inhibitors. However, neither BIC nor cBIC did improve upon inclusion of this covariate. Visual predictive check using prediction corrected concentrations of the covariate model showed that the majority of the observed data fell within the 90% prediction intervals and that the median tracks the middle of the observed data (Supplementary Fig. 1). Plotting observed versus predicted concentrations (Supplementary Fig. 2) revealed that our model tends to overestimate low concentrations in the population prediction. Further diagnostic plots did not reveal any anomalies (Supplementary Figs. 3 and 4).

### Clinical adverse events

**Fig. 4:**
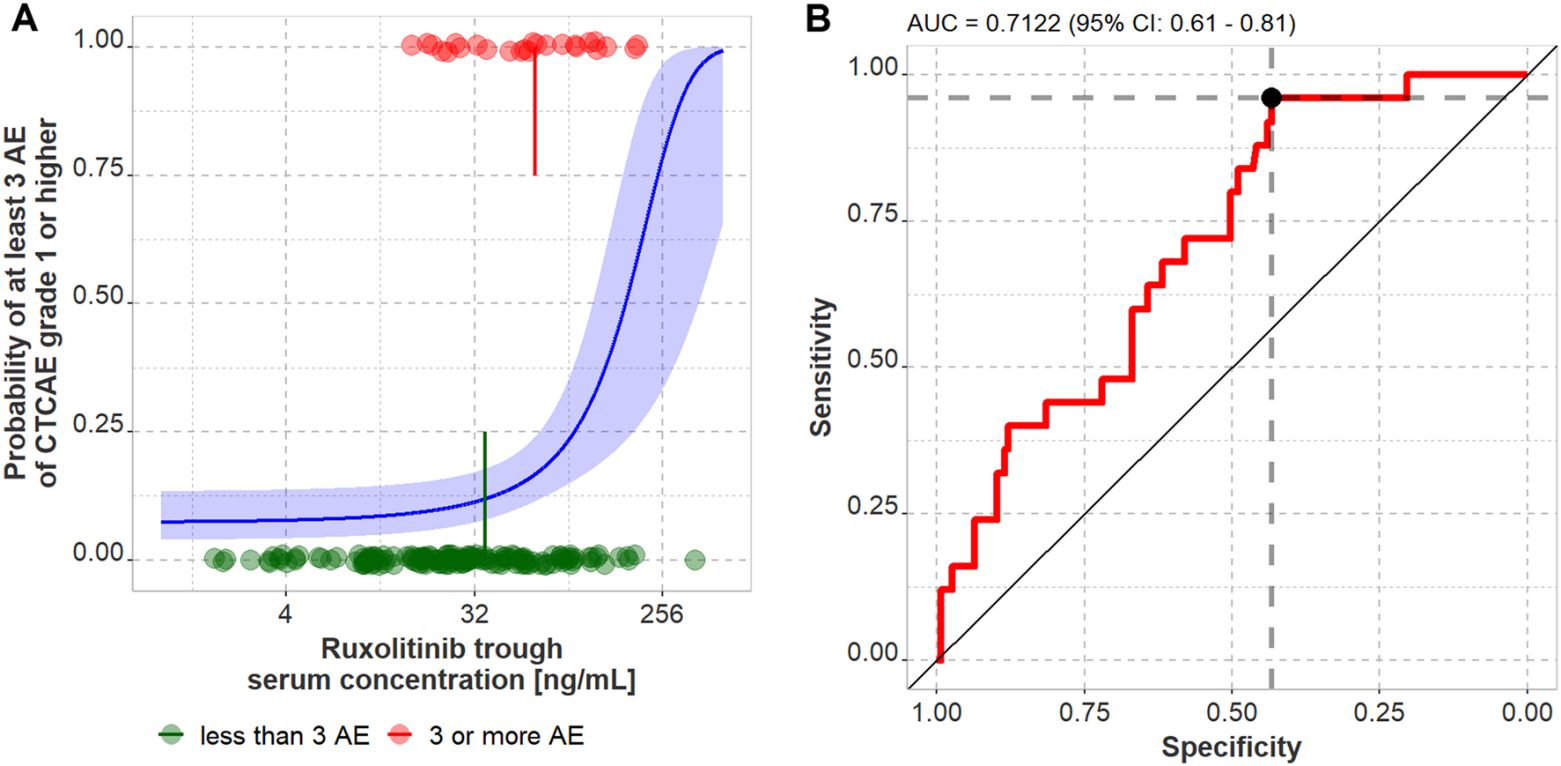
**a** Exploratory logistic regression investigating the effect of Ruxolitinib trough serum concentration on the probability of occurrence of at least three AE CTCAE grade 1 or higher. Dots represent individual measurements of all patients with available trough levels, lines and shaded area represent the estimate obtained from logistic regression and 95% confidence intervals, and the vertical lines represent the mean trough concentrations in both groups (35.9 vs. 62.8 ng/mL, respectively). **b** Receiver-operator-characteristics (ROC) analysis revealed an association between Ruxolitinib trough serum concentration and the risk to experience at least 3 AE of CTCAE grade 1 or higher. The optimal cut-off value was calculated using the Youden’s Index and is a Ruxolitinib trough serum concentration of 21.1 ng/mL. *AE* adverse event

Altogether, 302 clinical AE were reported at the time of blood sampling including 250 clinical AE of grade 1 and 46 AE of grade 2. Only 2.0% of all reported clinical AE were of grade 3, no AE of grade 4 or 5 were reported. On average 0.95 clinical AE of grade 1, 0.18 clinical AE of grade 2 and 0.02 clinical AE of grade 3 were reported at each sampling time point. For vertigo and cephalgia, a significant correlation between Ruxolitinib trough concentration and the occurrence of grade 1 AE on the same visit was observed (*p* < 0.05) (Supplementary Fig. 5). For all other documented clinical AE no significant correlation was found. Yet, using logistic regression without stratification for AE type and grade revealed that the risk of experiencing at least three AE of any grade is related to the Ruxolitinib trough serum concentration (*p* < 0.01) (Fig. 4). The odds ratio to experience at least three AE of any grade in patients with trough concentrations above the threshold found in the ROC analysis (21.1 ng/mL, see Fig. 4) was 8.8 (90% confidence interval: 3.3–21.4 by non-parametric bootstrapping).

### Laboratory adverse events

The prescribing information recommends dose reductions in GvHD patients with clinically significant thrombocytopenia, absolute neutrophil count < 1000/µl or bilirubin elevations of at least three times the upper limit of normal. Hence, we investigated correlations between thrombocyte count, absolute neutrophil count and bilirubin, respectively, and Ruxolitinib trough concentrations in patients of whom at least three (any dosing regimen) or two trough samples (10 mg twice daily) existed. Visual analysis of the data (Supplementary Figs. 6–8) did not reveal obvious effects of Ruxolitinib exposure on any parameter.

## Discussion

Data on Ruxolitinib exposure, especially in clinical settings, is scarce. Merienne et al. analyzed Ruxolitinib concentrations in 33 samples of 19 patients (underlying disease and dosing regimen unknown) and found remarkably high interpatient variability [31] which is in accordance with our findings. They reported a median *C*_ss_ _min_ of 11 ng/mL, a pharmacokinetic study conducted in eight healthy individuals found a *C*_ss_ _min_ of approximately 20 ng/mL at 30 mg daily [27] and the analysis of one *C*_ss_ _min_ in a MF patient showed a concentration of 15.8 ng/mL at 5 mg daily [32]. In contrast, median of individual mean *C*_ss_ _min_ at 10 mg twice daily was substantially higher (39.9 ng/mL) in our population suggesting higher exposure in GvHD patients. Yet, only limited conclusions can be drawn due to the small sample sizes and heterogenicity of the studies concerning underlying disease and dosing regimens.

To compensate for the unbalanced number of samples per patient in our study as a potential source for bias, we chose the population approach to predict Ruxolitinib concentrations based on a popPK model by Chen et al. established for patients with MF [29]. Interestingly, real-life concentrations were significantly higher than simulated Ruxolitinib concentrations which further indicates higher exposure in patients with GvHD compared to patients with MF. We were able to attribute this increase in drug exposure to a strong reduction in Ruxolitinib clearance (approximately 50%) whereas Ruxolitinib clearance in Chen’s study population was similar to that of healthy individuals [27]. It is important to note that this effect was independent of coadministration of relevant strong CYP inhibitors. Comedication with at least one strong CYP3A4 or CYP2C9 inhibitor further reduced Ruxolitinib clearance by 15%. Of course, records of concomitant medication could be incomplete, impacting the estimated effect of CYP-inhibiting medication. Additionally, the low number of patients investigated in our study, especially patients receiving no inhibitor, imposes the risk of over- or underestimating the effect of CYP inhibitors. This was underlined by the lack of improvement in BIC and cBIC. Yet, three patients contributed samples to both groups. Also, our findings are in line with a previously published popPK analysis characterizing pharmacokinetics of Ruxolitinib in patients with aGvHD which also found lower oral clearance (66.7%) and a slower rate of absorption (28%) in comparison to MF patients [33]. The fact that we could not confirm the influence of sex and body weight on Ruxolitinib clearance as reported by Chen et al. might be due to the small sample size. However, Chen et al. concluded that these effects were not clinically significant. Overall, the developed popPK models resulted in an adequate description of Ruxolitinib pharmacokinetics in GvHD patients, but the observed covariate effect should be interpreted with care due to the low number of patients without CYP-inhibiting comedication and the explorative nature of the analysis (no *p* value correction for multiple testing).

Specifics of allo-HSCT recipients should be taken into consideration when speculating about potential factors contributing to altered drug exposure and clearance. First, even though the observed reduction in clearance was independent of the concomitant uptake of relevant strong CYP inhibitors, it can be presumed that further drug–drug interactions caused by a combination of several moderate and weak CYP3A4 or CYP2C9 inhibitors affected clearance, especially considering the extend of polypharmacy in allo-HSCT recipients [34, 35] and in our population. Due to the small proportion of patients receiving multiple CYP inhibitors at the same time, this covariate could not be included in the model. However, it is noteworthy that we observed remarkably high Ruxolitinib trough concentrations in two patients receiving dual CYP inhibition with Posaconazole (strong CYP3A4 inhibitor) and Atorvastatin (moderate CYP3A4 and CYP2C9 inhibitor) or Amiodarone (moderate CYP2C9 and weak CYP3A4 inhibitor) (Supplementary Fig. 9). Second, changes in hepatic Ruxolitinib clearance are likely even though no patients with high-grade liver dysfunction were included in our study. Mild to moderate liver dysfunction caused by conditioning regimens, previous conventional chemotherapy, hepatotoxic comedication, iron overload or low-grade liver GvHD are frequent, yet often underdiagnosed complications. Third, impaired renal function is common after allo-HSCT [22–24] and might contribute to increased Ruxolitinib exposure. In our population eGFR was no significant covariate on clearance, but the small number of samples included with an eGFR < 60 mL/min imposes a limitation to our study. As sarcopenia is common in allo-HSCT recipients [36–38], eGFR might fail to detect minor renal dysfunction in our cohort. Fourth, other pharmacokinetic properties might be modified, e.g., reduced gastrointestinal absorption due to modification of the gut microbiota.

Both clinical and laboratory AE were frequent but mild in our study population which is in contrast to the prescribing information where a substantial number of grade 3–4 AE and treatment discontinuations were reported [16]. Presumably, AE are underestimated in our population for several reasons: AE were only recorded at the time of blood sampling. Therefore, the number of AE does not reflect the actual occurrence of AE during the entire interval. Additionally, all our samples were collected from outpatients. Especially AE ≥ grade 3 are likely to lead to hospital admission and where consequently missed. Lastly, median treatment duration at enrollment was 76 days. As severe and dose-limiting AE often occur shortly after initiation of therapy, their number is probably underestimated. Therefore, patients who tolerate Ruxolitinib well are over-represented in our study population. Still, we could show a highly significant correlation between vertigo and cephalgia CTCAE grade 1 and Ruxolitinib exposure. Additionally, logistic regression revealed a highly significant correlation between the risk of experiencing at least three AE of any grade and Ruxolitinib trough serum concentration and non-parametric testing showed significantly higher individual mean trough concentrations (60.6 ng/mL) in patients experiencing AE-related dose reduction (before dosage adaptation) in comparison to patients not requiring dose reduction. The fact that the odds ratio to experience at least three AE of any grade in patients with trough concentrations above 21.1 ng/mL is 8.8 underscores the impact of trough concentrations on AE. However, the variability in both groups is large and several patients tolerated equally high mean trough concentrations well. Nevertheless, these results give rise to the hypothesis that patients with increased Ruxolitinib trough concentrations might be at higher risk for AE and hence dose reduction or treatment discontinuation. This hypothesis warrants further systematic investigation.

When analyzing our data on Ruxolitinib exposure it has to be kept in mind that our study was conducted in a real-life clinical setting. Therefore, Ruxolitinib administration was self-reported by the patients and inaccuracy in reported times might have biased the data. To test our hypothesis that the occurrence of AE is associated with elevated Ruxolitinib concentrations in GvHD patients, a larger prospective cohort study including patients at therapy initiation should be conducted and any reported AE should lead to analysis of Ruxolitinib concentrations, especially AE leading to hospital admission. A significant number of patients with renal or hepatic impairment should be included as recommendations on dose reductions are currently based on studies conducted in otherwise healthy subjects after single-dosing [39]. Moreover, the impact of intestinal and liver GvHD should be investigated. Subtherapeutic Ruxolitinib concentrations might contribute to the low response rates in patients with gastrointestinal involvement reported in several publications [12, 40, 41]. A previous popPK model has identified the stage of liver involvement as a covariate for Ruxolitinib clearance in patients with aGvHD [33]. Yet, data on real-life concentrations in patients with liver GvHD are scarce. As no patient of our population discontinued therapy due to a lack of effectiveness, no conclusions on subtherapeutic Ruxolitinib exposure and treatment outcome can be drawn.

Taken together, our findings demonstrate a high degree of variability in Ruxolitinib concentrations and significantly higher drug exposure in GvHD patients in comparison to patients with MF receiving the same dose, most likely caused by frequent comedication with several moderate and weak CYP3A4 and CYP2C9 inhibitors, often in combination with azoles (strong CYP3A4 inhibitors). However, the reduced recommended maintenance dose of Ruxolitinib in GvHD patients (10 mg twice daily) in comparison to MF patients (20 mg twice daily) seems adequate to compensate for the reduced clearance observed in the GvHD population [16]. Nevertheless, our results suggest a relationship between elevated Ruxolitinib trough concentrations and the risk for toxicity and AE-related dose reduction. Therefore, therapeutic drug monitoring of Ruxolitinib might be beneficial for risk groups, especially at the beginning of therapy, e.g., patients receiving extensive comedication with multiple CYP3A4 or CYP2C9 inhibitors or patients with dose-limiting AE.

## Supplementary Information

Below is the link to the electronic supplementary material.

Supplementary file1 (DOCX 4583 KB)

## Data Availability

All data generated and analyzed during this study are available from the corresponding author on reasonable request.

## Code availability

The popPk model and R-code will be made available from the corresponding author on reasonable request.
