# Supplementary material for: Ruxolitinib exposure in patients with acute and chronic graft versus host disease in routine clinical practice—a prospective single-center trial
Source: Cancer Chemother Pharmacol. 2021 Sep 10;88(6):973–83. doi: 10.1007/s00280-021-04351-w (PMC8536600; doi:10.1007/s00280-021-04351-w)
Supplement: Supplementary file 1 — Supplementary file1 (DOCX 4583 KB) [file 280_2021_4351_MOESM1_ESM.docx]

**Electronic supplementary material**

**Ruxolitinib exposure in patients with acute and chronic graft versus host disease in routine clinical practice – a prospective single-center trial**

**Running title: Ruxolitinib exposure in graft versus host disease**

Nora Isberner^1, *^, Sabrina Kraus^1^, Götz Ulrich Grigoleit^1, a^, Fatemeh Aghai^1^, Max Kurlbaum^2^, Sebastian Zimmermann^3^, Hartwig Klinker^1^, Oliver Scherf-Clavel^3^

^1^ University Hospital Würzburg, Department of Internal Medicine II, Germany

* Corresponding author: Dr. Nora Isberner, University Hospital Würzburg, Department of Internal Medicine II, Oberdürrbacher Strasse 6, 97080 Würzburg, Germany, e-mail address: isberner_n@ukw.de,
ORICD: 0000-0001-7217-9191

^a^ Present address: Helios Hospital Duisburg, Department of Hematology, Oncology and Immunology, Germany

^2^ University Hospital Würzburg, Department of Internal Medicine I and Core Unit Clinical Mass Spectrometry, Division of Endocrinology and Diabetology, Germany

^3^ Institute of Pharmacy and Food Chemistry, University of Würzburg, Germany

**Supplementary Tables**

**Supplementary Table 1. Sample characteristics**

| **Sample characteristic** | **No. of samples** | **%** |
| --- | --- | --- |
| **Total**  Included samples (in calibration range)  Excluded samples < LLOQ | **262**  254  8 | **100**  96.9  3.1 |
| **Samples / patient, median (range)** | 8 (1 – 23) |  |
| **Trough sample** | 182 | 69.5 |
| **Daily Dose (mg), mean (range)**  20 mg daily (samples < LLOQ)  <20 mg daily (samples < LLOQ)  > 20 mg daily (samples < LLOQ) | 17.8 (5-40)  175 (5)  75 (2)  4 (1) | 66.8  28.6  1.5 |
| **Dosing scheme**  Twice daily  Once daily  Three times per day | 229  7  26 | 87.4  2.7  9.9 |
| **Samples of patients with eGFR < 60 mL/min/1.73m^2^ at sample collection** | 74 | 28.2 |

*LLOQ* lower limit of quantification (2 ng/mL), *eGFR* estimated glomerular filtration rate

**Supplementary Table 2. Baseline laboratory parameters at inclusion**

| **Parameter** | **Mean** | **Median** | **SD** | **Range** |
| --- | --- | --- | --- | --- |
| eGFR (mL/min/1.73m²) | 70.2 | 67.2 | 19.6 | 30.5-119.0 |
| Serum creatinine (mg/dL) | 1.06 | 1.04 | 0.35 | 0.51-2.09 |
| Serum albumin (g/dL) | 4.4 | 4.3 | 0.38 | 3.9-5.1 |
| Total cholesterol (mg/dL) | 248 | 218 | 58 | 158-383 |
| Total bilirubin (mg/dL) | 0.34 | 0.30 | 0.18 | 0.2-1.1 |
| Hemoglobin (g/dL) | 11.95 | 12.10 | 1.62 | 8.0-16.3 |
| White blood cell count (1/µL) | 6172 | 5500 | 2525 | 3400-14300 |
| Absolute neutrophil count (1/µL) | 3853 | 3310 | 2233 | 820-10550 |
| Platelet count (1/µL) | 295966 | 271000 | 160468 | 29000-639000 |
| AST (U/L) | 40.3 | 37.1 | 17.2 | 11.7-99.0 |
| ALT (U/L) | 63.4 | 43.6 | 66.2 | 16.2-331.6 |

*eGFR* estimated glomerular filtration rate, *AST* aspartate aminotransferase, *ALT* alanine aminotransferase

**Supplementary Table 3. Population pharmacokinetic parameters for the base model and the covariate model including strong CYP inhibitors as covariates**

|  | Base model | | Covariate model | |
| --- | --- | --- | --- | --- |
|  | Population mean | % RSE | Population mean | % RSE |
| k_a_ (h^-1^) | 4.12 | *fixed*^**^ | 4.12 | *fixed*^**^ |
| T_Lag_ (h) | 0.0545 | *fixed*^**^ | 0.0545 | *fixed*^**^ |
| CL/F (L/h) | 8.55 | 11.3 | 9.74 | 11.1 |
| V_c_/F (L) | 64.8 | 12.3 | 61.9 | 10.9 |
| Q/F (L/h) | 2.53 | *fixed*^**^ | 2.53 | *fixed*^**^ |
| V_per_/F (L) | 11.2 | *fixed*^**^ | 11.2 | *fixed^**^* |
| *β*_CL, STRONG_INH_ | - | - | -0.16 | 30.5 |
| *ω*_CL/F_^*^ | 0.52 | 16.4 | 0.48 | 18.2 |
| *ω*_V_^*^ | 0.25 | 83.6 | 0.29 | 41.3 |
| RUV b^*^ | 0.50 | 5.61 | 0.50 | 5.51 |
| AIC | -992.92 | | -995.68 | |
| -2LL | -1002.92 | | -1007.68 | |
| BIC | -975.1 | | -974.3 | |
| BICc | -979.49 | | -978.69 | |

^*^ as standard deviations of respective normal distribution
^**^ obtained from Chen X, Williams WV, Sandor V, Yeleswaram S. Population Pharmacokinetic

Analysis of Orally-Administered Ruxolitinib (INCB018424 Phosphate) in Patients With Primary

Myelofibrosis (PMF), Post-Polycythemia Vera Myelofibrosis (PPV-MF) or Post-Essential

Thrombocythemia Myelofibrosis (PET MF). J Clin Pharmacol. 2013;53(7):721–30

*RSE* relative standard error, *RUV* residual unexplained variability*, AIC* Akaike Information Criteria, -*2LL* -2-log-likelihood, *BICc* corrected Bayesian Information Criteria, *BIC* Bayesian Information Criteria

**Supplementary Table 4. Comedication of all patients stratified by potential to induce or inhibit CYP3A4 or CYP2C9**

| **Drug** | **CYP3A4** | **Strength** | **CYP2C9** | **Strength** | **Reference**^a^ |
| --- | --- | --- | --- | --- | --- |
| **Strong CYP3A4 or CYP2C9 inhibitors / inducers** | | | | |  |
| Fluconazole | inhibitor | strong | inhibitor | strong | (1) |
| Loperamide | inhibitor | strong | - | - | (1) |
| Posaconazole | inhibitor | strong | - | - | (1) |
| Voriconazole | inhibitor | strong | inhibitor | weak | (1,2) |
| **Moderate CYP3A4 or CYP2C9 inhibitors / inducers** | | | | |  |
| Atorvastatin | inhibitor | moderate | Inhibitor | moderate | (1,3) |
| Deferasirox | inducer | moderate | - | - | (1,4) |
| Amiodarone | inhibitor | weak | inhibitor | moderate | (1,5) |
| Fluvastatin | inhibitor | unknown | inhibitor | moderate | (1) |
| **Weak CYP3A4 or CYP2C9 inhibitors / inducers** | | | | |  |
| Cyclosporine | inhibitor | weak | - | - |  |
| Mirtazapine | inhibitor | weak | inhibitor | - | (1) |
| Ranitidine | inhibitor | weak | inhibitor | - | (1) |
| Sirolimus | inhibitor | weak | inhibitor | weak | (1) |
| Candesartan | - | - | inhibitor | weak | (1) |
| **CYP3A4 or CYP2C9 inhibitors / inducers of unknown strength**^b^ | | | | |  |
| Azithromycin | inhibitor | unknown | - | - | (1) |
| Budesonide | inhibitor | unknown | inhibitor | unknown | (1) |
| Etoricoxib | inhibitor | unknown | inhibitor | unknown | (1) |
| Fenofibrate | - | - | inhibitor | unknown | (1) |
| Lercanidipine | inhibitor | unknown | - | - | (1) |
| Letermovir | inhibitor | unknown | inducer | unknown | (1) |
| Simvastatin | inhibitor | unknown | inhibitor | unknown | (1) |
| Sulfamethoxazole | inhibitor | unknown | inhibitor | unknown | (1,6) |
| Nifedipine | inducer / inhibitor | unknown | inducer / inhibitor | unknown | (1) |
| Omeprazole | inducer / inhibitor | unknown | - | - | (1) |
| Quinine | inducer / inhibitor | unknown | - | - | (1) |
| Hydrocortisone | inducer | unknown | inducer | unknown | (1) |
| Metamizole | inducer | unknown | - | - | (1) |
| Clopidogrel | - | - | inhibitor | unknown | (1) |
| Torasemide | - | - | inhibitor | unknown | (1) |
| **Comedication without known effects on CYP3A4 or CYP2C9 metabolism** | | | | |  |
| Acetylsalicylic acid | - | - | - | - | (1) |
| Acyclovir | - | - | - | - | (1) |
| Allopurinol | - | - | - | - | (1) |
| Amitriptyline | - | - | - | - | (1) |
| Ampicillin | - | - | - | - | (1) |
| Apixaban | - | - | - | - | (1) |
| Atovaquone | - | - | - | - | (1) |
| Bisacodyl | - | - | - | - | (1) |
| Bisoprolol | - | - | - | - | (1) |
| Brivudine | - | - | - | - | (1) |
| Budesonide | - | - | - | - | (1) |
| Calcium | - | - | - | - | (1) |
| Carvedilol | - | - | - | - | (1) |
| Digitoxin | - | - | - | - | (1) |
| Dihydralazine | - | - | - | - | (1) |
| Dydrogesterone | - | - | - | - | (1) |
| Eltrombopag | - | - | - | - | (1) |
| Empagliflozin | - | - | - | - | (1) |
| Enoxaparin | - | - | - | - | (1) |
| Eplerenone | - | - | - | - | (1) |
| Escitalopram | - | - | - | - | (1) |
| Etanercept | - | - | - | - | (1) |
| Etoricoxib | - | - | - | - | (1) |
| Fentanyl | - | - | - | - | (1) |
| Filgrastim | - | - | - | - | (1) |
| Folic acid | - | - | - | - | (1) |
| Fosfomycin | - | - | - | - | (1) |
| Gabapentin | - | - | - | - | (1) |
| Hydrochlorothiazide | - | - | - | - | (1) |
| Hydrocortisone | - | - | - | - | (1) |
| Hydromorphone | - | - | - | - | (1) |
| Ibuprofen | - | - | - | - | (1) |
| Iron | - | - | - | - | (1) |
| Isosorbide dinitrate | - | - | - | - | (1) |
| Levothyroxine | - | - | - | - | (1) |
| Magnesium | - | - | - | - | (1) |
| Meropenem | - | - | - | - | (1) |
| Mesalazine | - | - | - | - | (1) |
| Metformin | - | - | - | - | (1) |
| Methocarbamol | - | - | - | - | (1) |
| Metoclopramide | - | - | - | - | (1) |
| Metoprolol | - | - | - | - | (1) |
| Morphine | - | - | - | - | (1) |
| Moxonidine | - | - | - | - | (1) |
| Mycophenolate mofetil | - | - | - | - | (1) |
| Pantoprazole | - | - | - | - | (1) |
| Polyethylene glycol | - | - | - | - | (1) |
| Potassium | - | - | - | - | (1) |
| Potassium iodide | - | - | - | - | (1) |
| Pravastatin | - | - | - | - | (1) |
| Prednisolone | - | - | - | - | (1) |
| Pregabalin | - | - | - | - | (1) |
| Ramipril | - | - | - | - | (1) |
| Rizatriptan | - | - | - | - | (1) |
| Scopolamine | - | - | - | - | (1) |
| Simethicone | - | - | - | - | (1) |
| Sucralfate | - | - | - | - | (1) |
| Sulbactam | - | - | - | - | (1) |
| Tamsulosin | - | - | - | - | (1) |
| Tilidine/Naloxone | - | - | - | - | (1) |
| Torasemide | - | - | - | - | (1) |
| Trimethoprim | - | - | - | - | (1,6) |
| Trospium | - | - | - | - | (1) |
| Urapidil | - | - | - | - | (1) |
| Ursodeoxycholic acid | - | - | - | - | (1) |
| Valganciclovir | - | - | - | - | (1) |
| Vancomycin | - | - | - | - | (1) |
| Vitamin B1 | - | - | - | - | (1) |
| Vitamin B6 | - | - | - | - | (1) |
| Vitamin D | - | - | - | - | (1) |
| Zolpidem | - | - | - | - | (1) |

^a^ (1) www.drugbank.ca
(2) Jeong S, Nguyen PD, Desta Z. Comprehensive In Vitro Analysis of Voriconazole Inhibition of Eight Cytochrome P450 (CYP) Enzymes: Major Effect on CYPs 2B6, 2C9, 2C19, and 3A. Antimicrob Agents Chemother. 2009 Feb;53(2):541–51
(3) Jacobsen W, Kirchner G, Hallensleben K, Mancinelli L, Deters M, Hackbarth I, et al. Comparison of cytochrome P-450-dependent metabolism and drug interactions of the 3-hydroxy-3-methylglutaryl-CoA reductase inhibitors lovastatin and pravastatin in the liver. Drug Metab Dispos Biol Fate Chem. 1999 Feb;27(2):173–9.
(4) Skerjanec A, Wang J, Maren K, Rojkjaer L. Investigation of the pharmacokinetic interactions of deferasirox, a once-daily oral iron chelator, with midazolam, rifampin, and repaglinide in healthy volunteers. J Clin Pharmacol. 2010 Feb;50(2):205–13.
(5) Ohyama K, Nakajima M, Suzuki M, Shimada N, Yamazaki H, Yokoi T. Inhibitory effects of amiodarone and its N-deethylated metabolite on human cytochrome P450 activities: Prediction of in vivo drug interactions. Br J Clin Pharmacol. 2000 Mar;49(3):244–53.
(6) Wen X, Wang J-S, Backman JT, Laitila J, Neuvonen PJ. Trimethoprim and sulfamethoxazole are selective inhibitors of CYP2C8 and CYP2C9, respectively. Drug Metab Dispos Biol Fate Chem. 2002 Jun;30(6):631–5.

^b^ Inhibitors / Inducers of unknown strength according to www.drugbank.ca were considered weak inhibitors / inducers if literature research did not reveal other aspects

**Supplementary Figures**


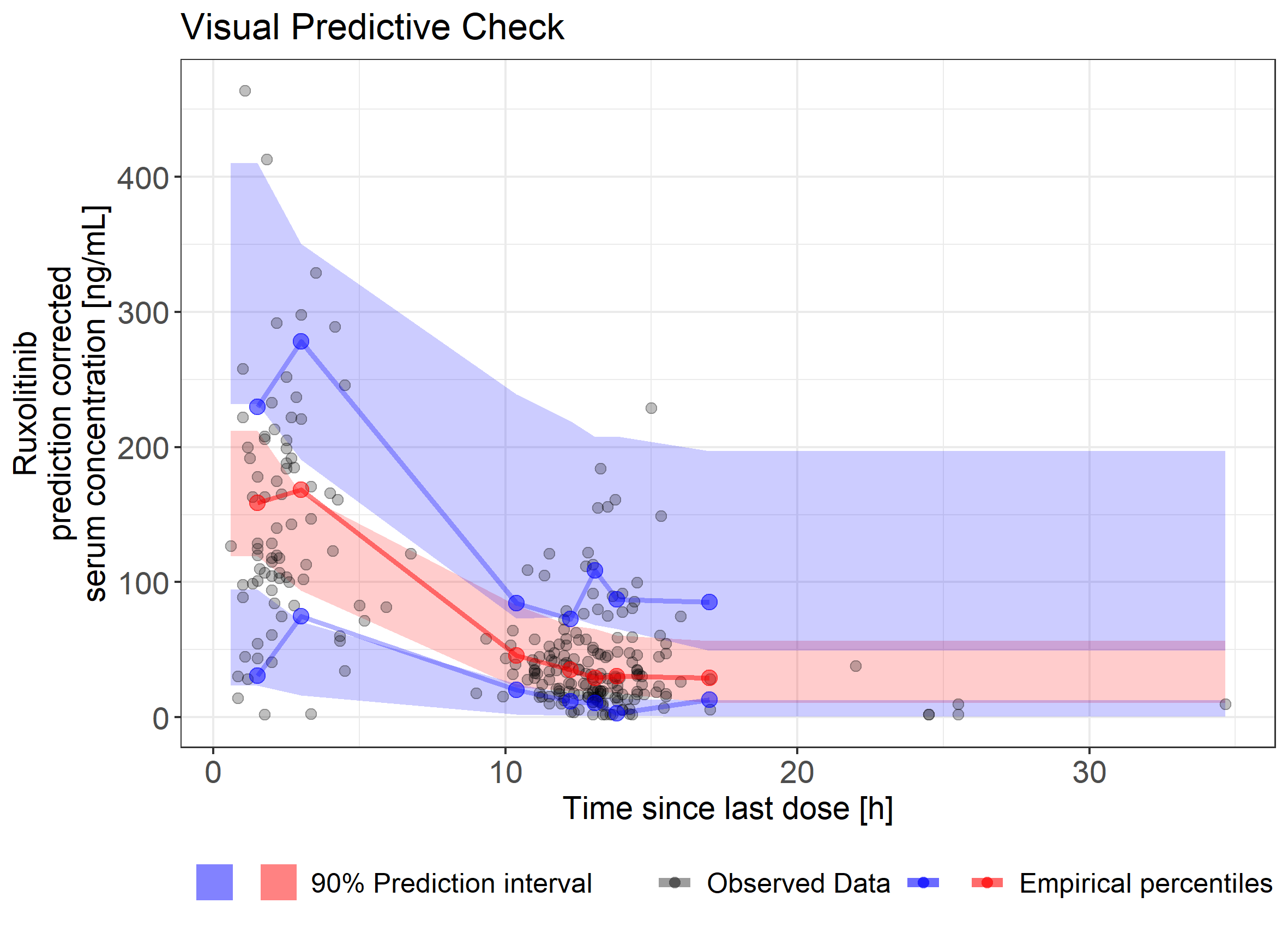


**Supplementary Fig. 1.** Visual predictive Check. Solid lines represent the 5th (lower blue), 50th (red), and 95th (upper blue) percentile of the observed data. Shaded regions represent the 90% confidence intervals surrounding the 5th, 50th, and 95th percentile of the predicted data. Overall, the plot demonstrates that the model predictions captured the majority of observed Ruxolitinib concentrations between the 5th and 95th percentiles of the simulated values.


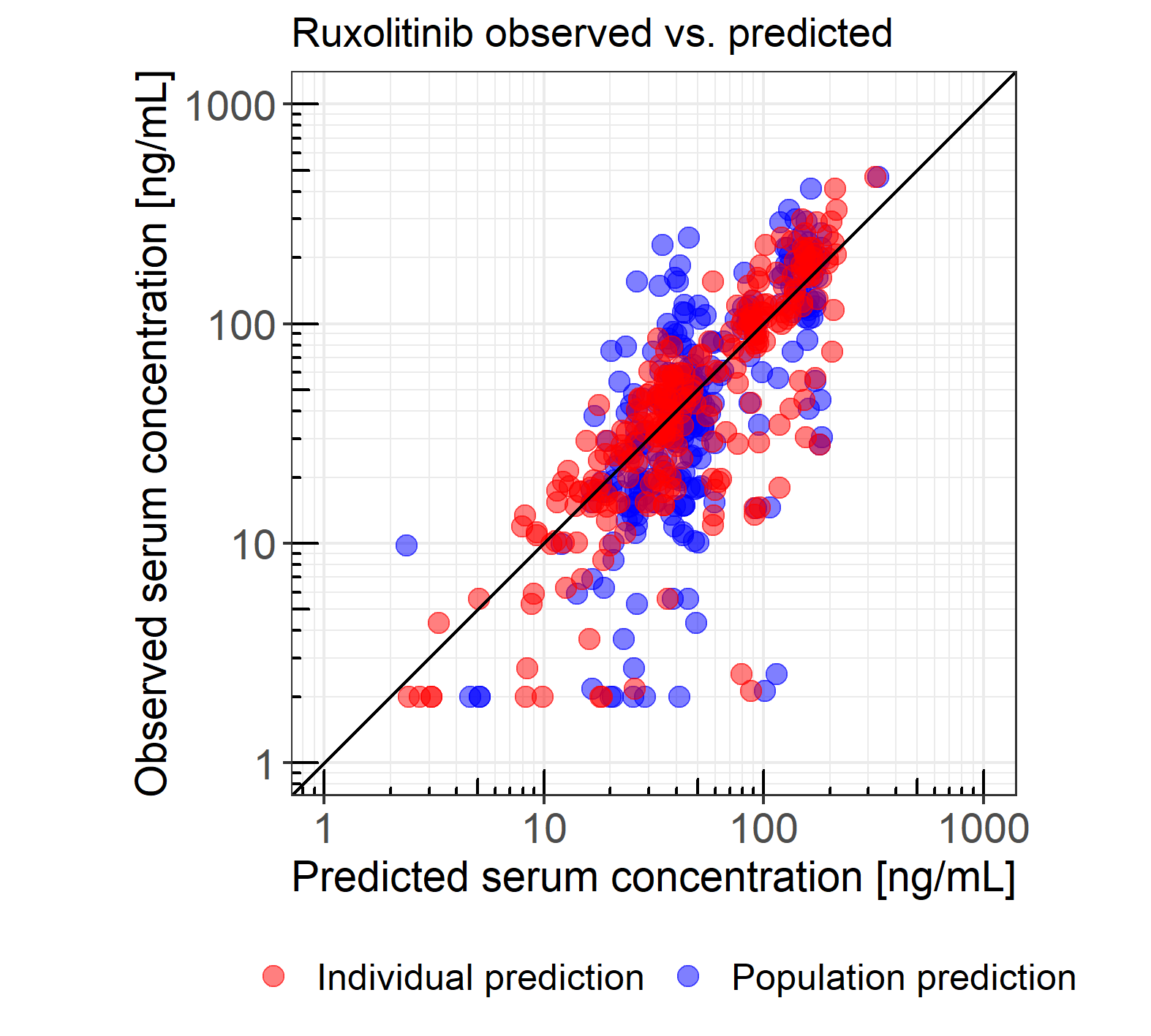


**Supplementary Fig. 2:** Observed versus predicted Ruxolitinib serum concentrations of the covariate model.

| 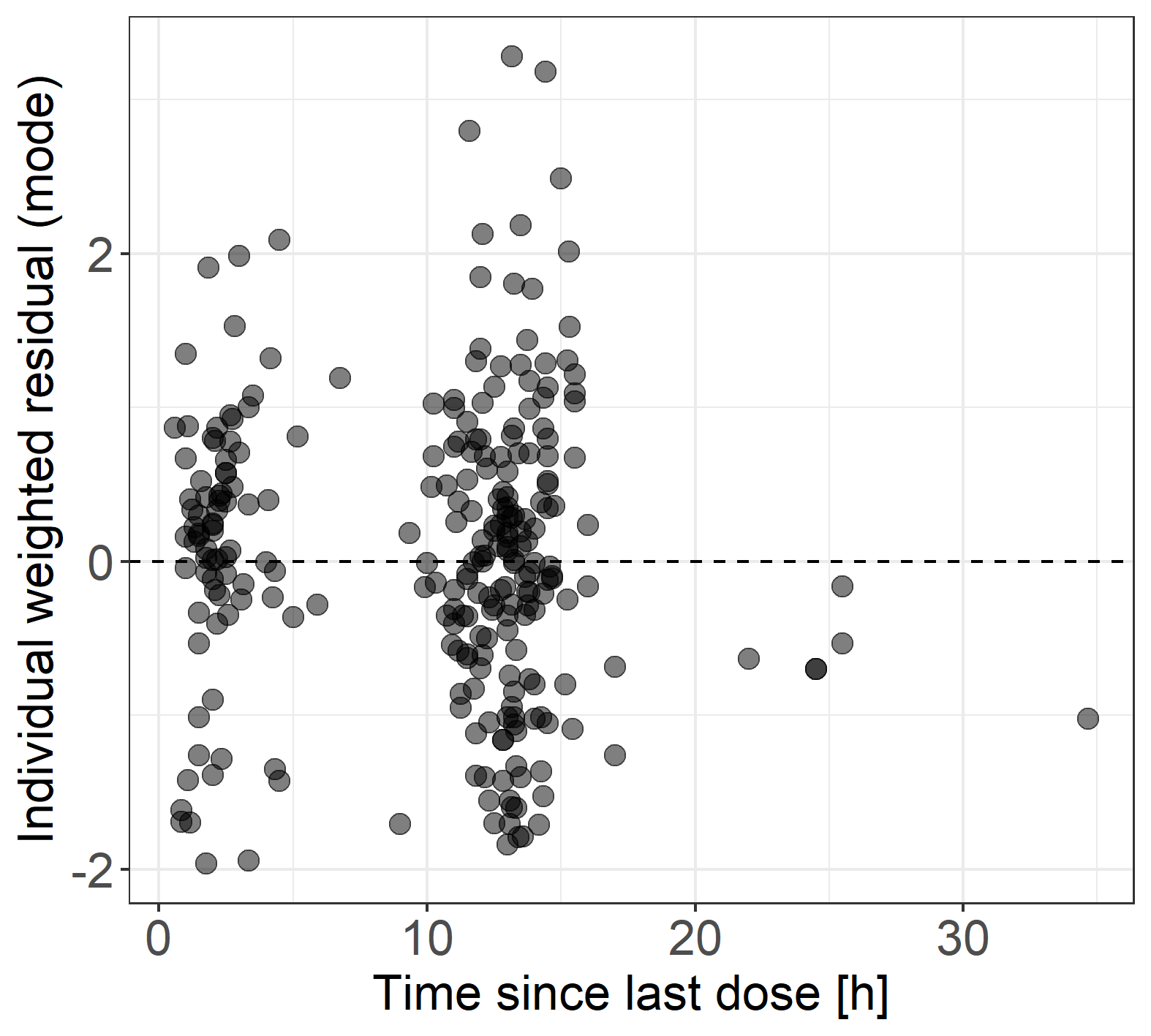 | 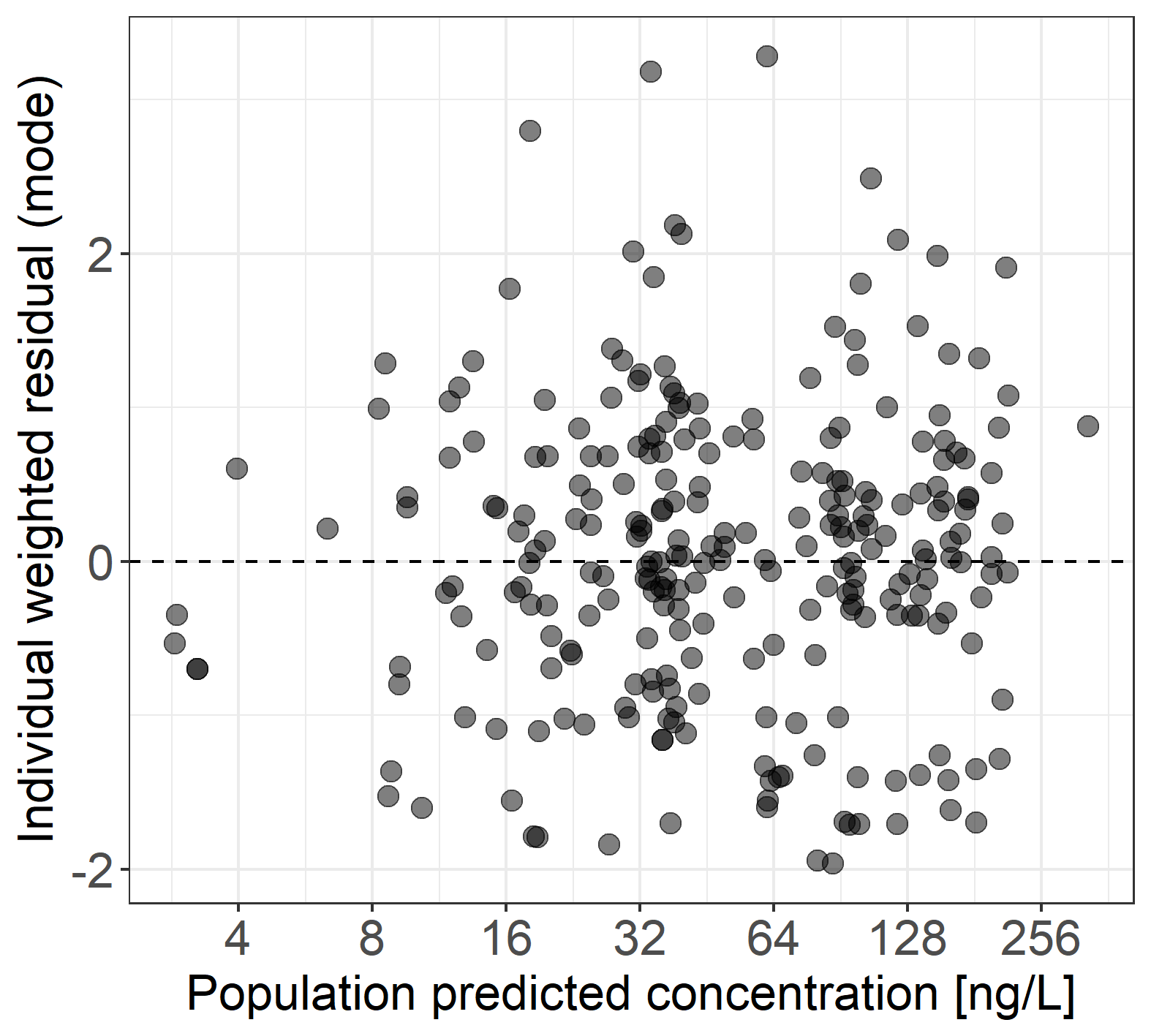 |
| --- | --- |

**Supplementary Fig. 3:** Individual weighted residual (IWRES) versus time and predicted concentration, respectively.


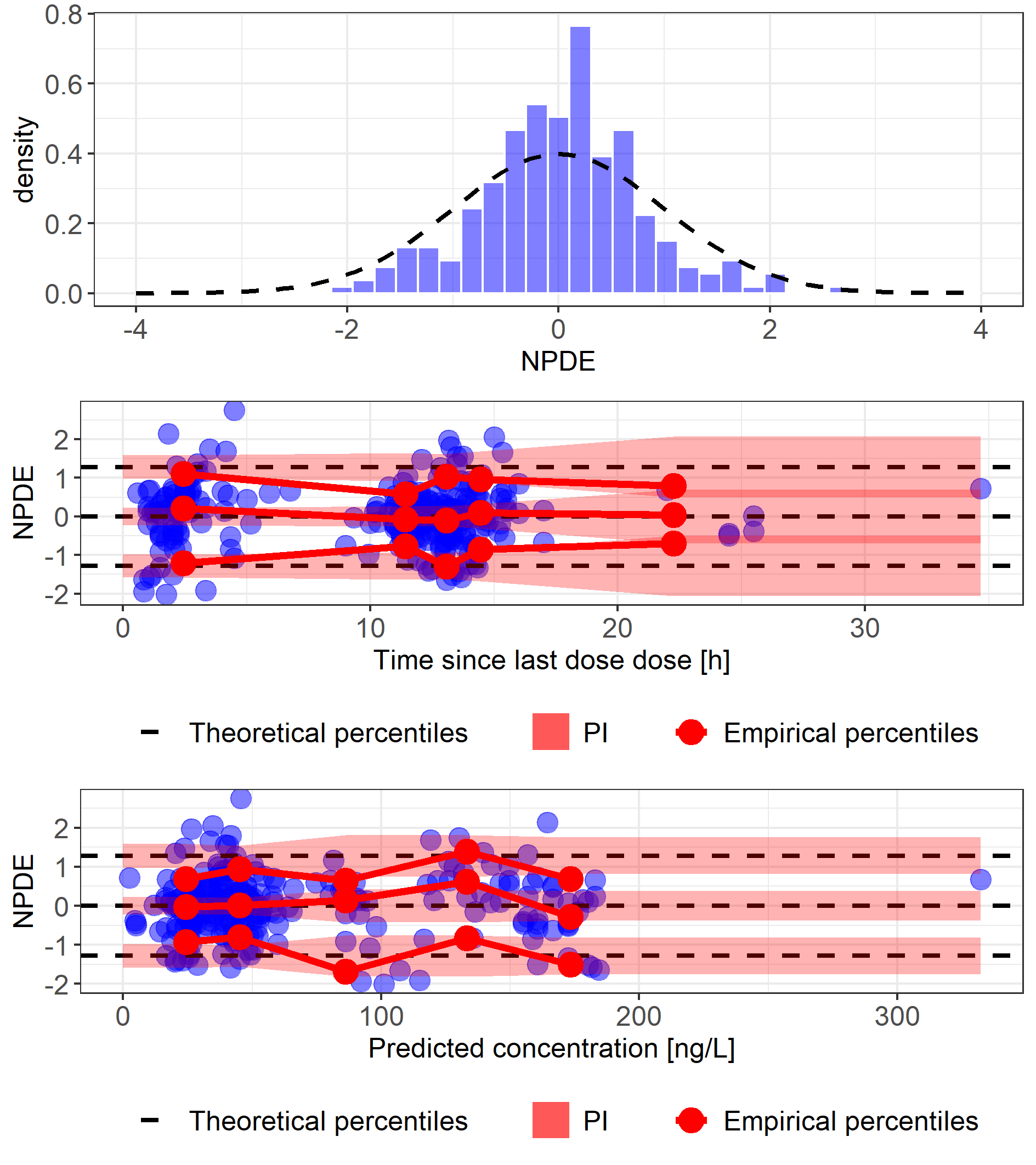


**Supplementary Fig. 4:** NPDE plots.
NPDE normalized prediction distribution error


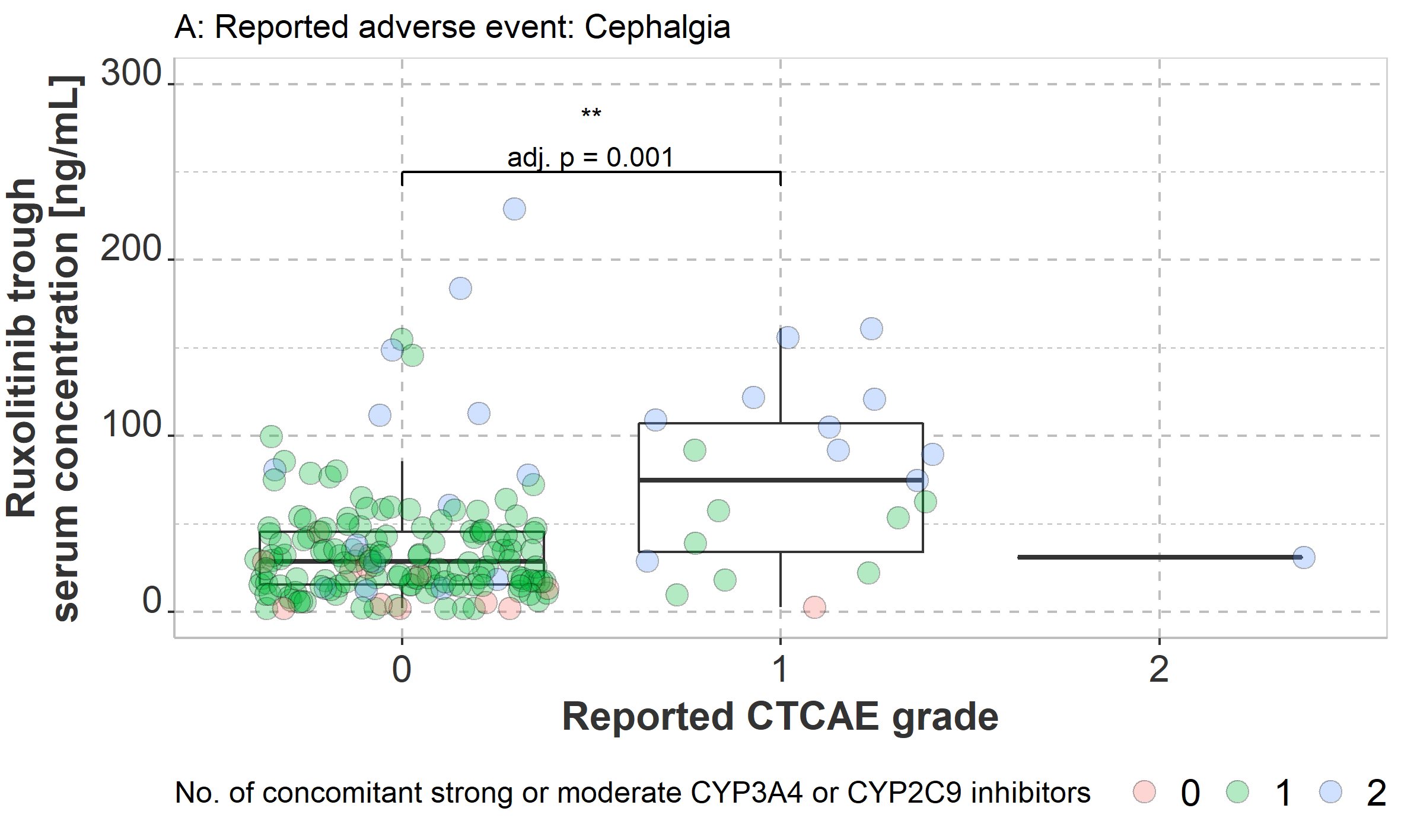

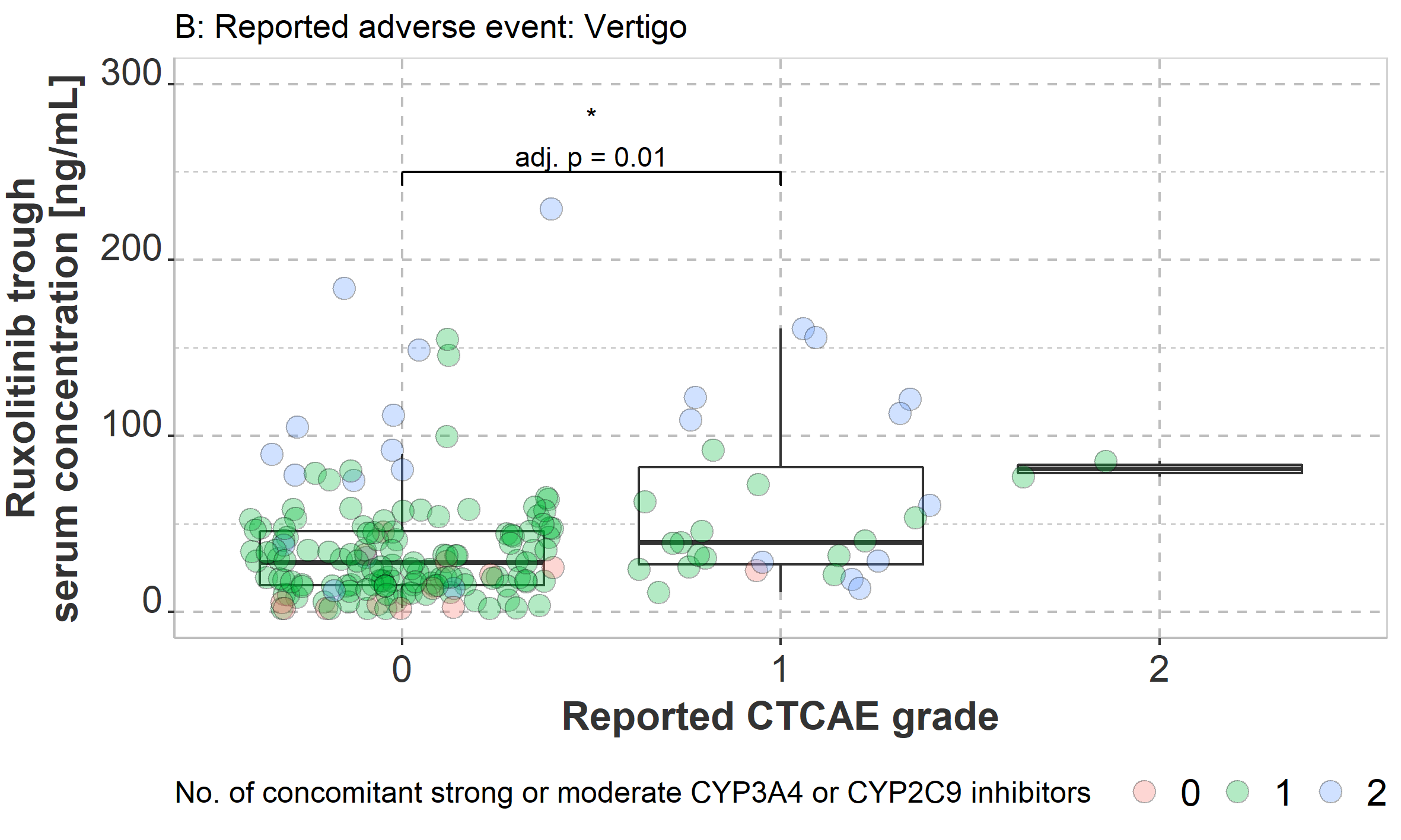


**Supplementary Fig. 5:** Ruxolitinib trough serum concentrations stratified by CTCAE grade of adverse events (cephalgia (a) or vertigo (b))

| **Supplementary Fig. 6:** Thrombocyte count vs. Ruxolitinib trough serum concentration | |
| --- | --- |
| 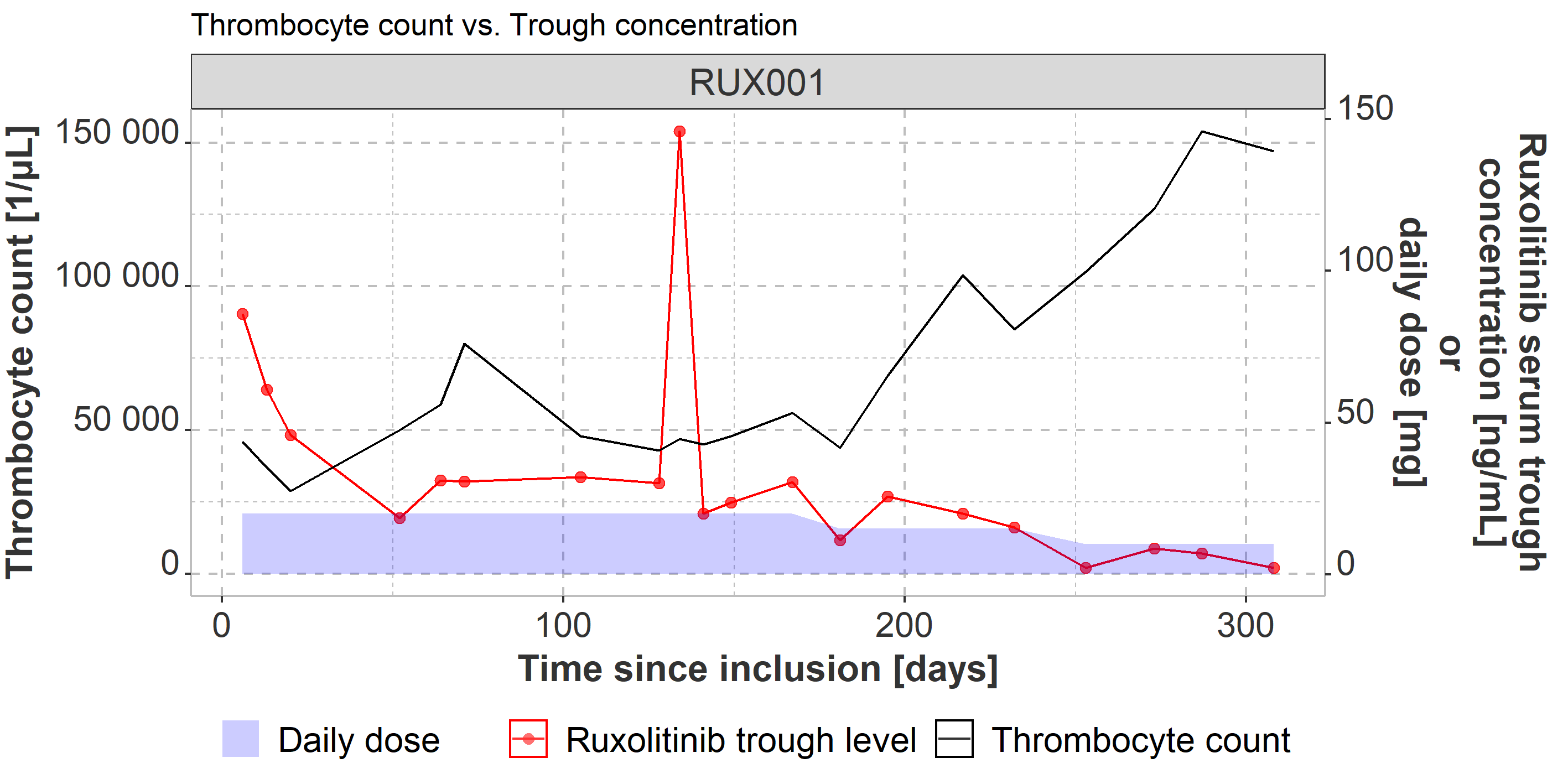 | 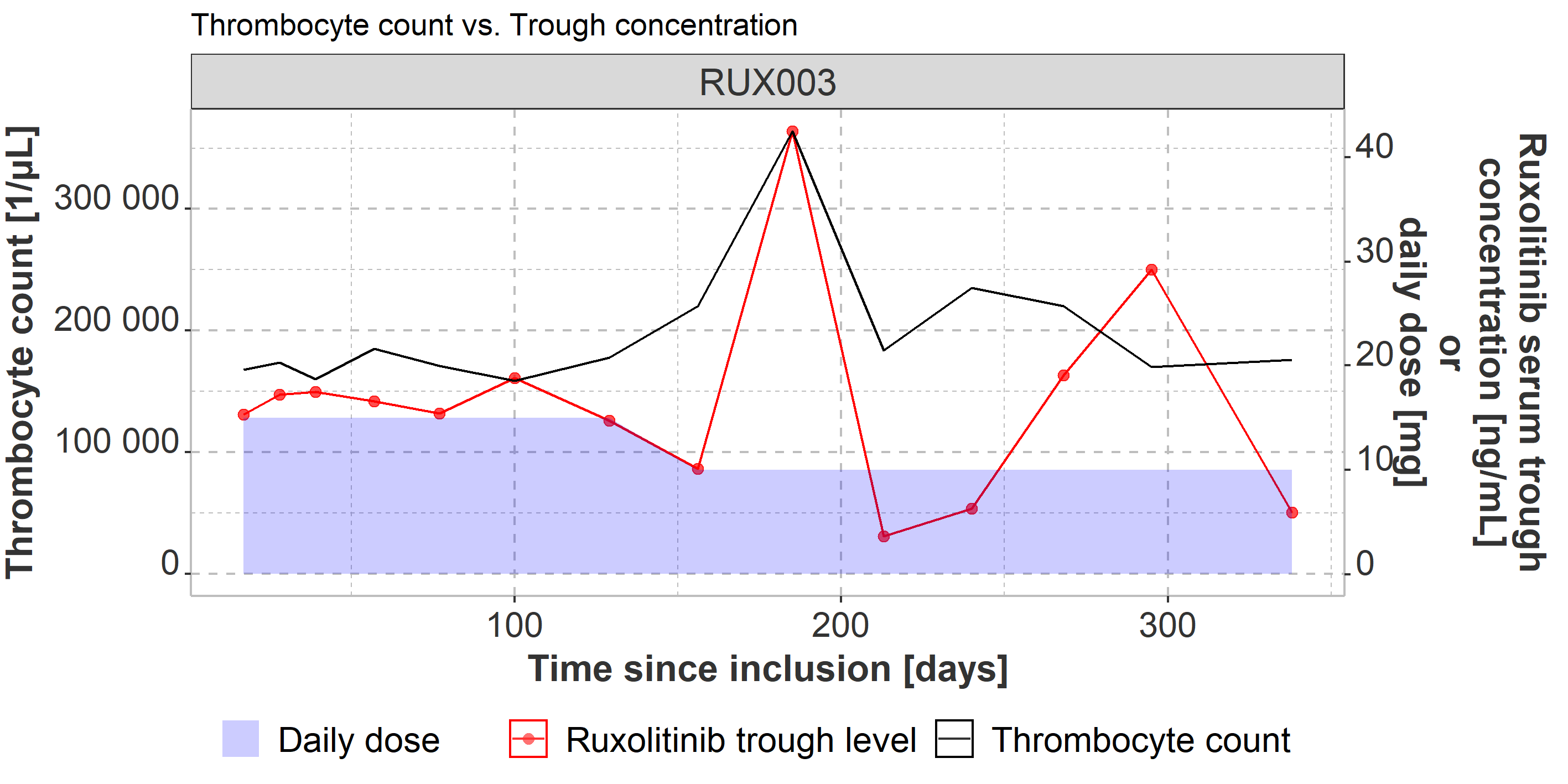 |
| 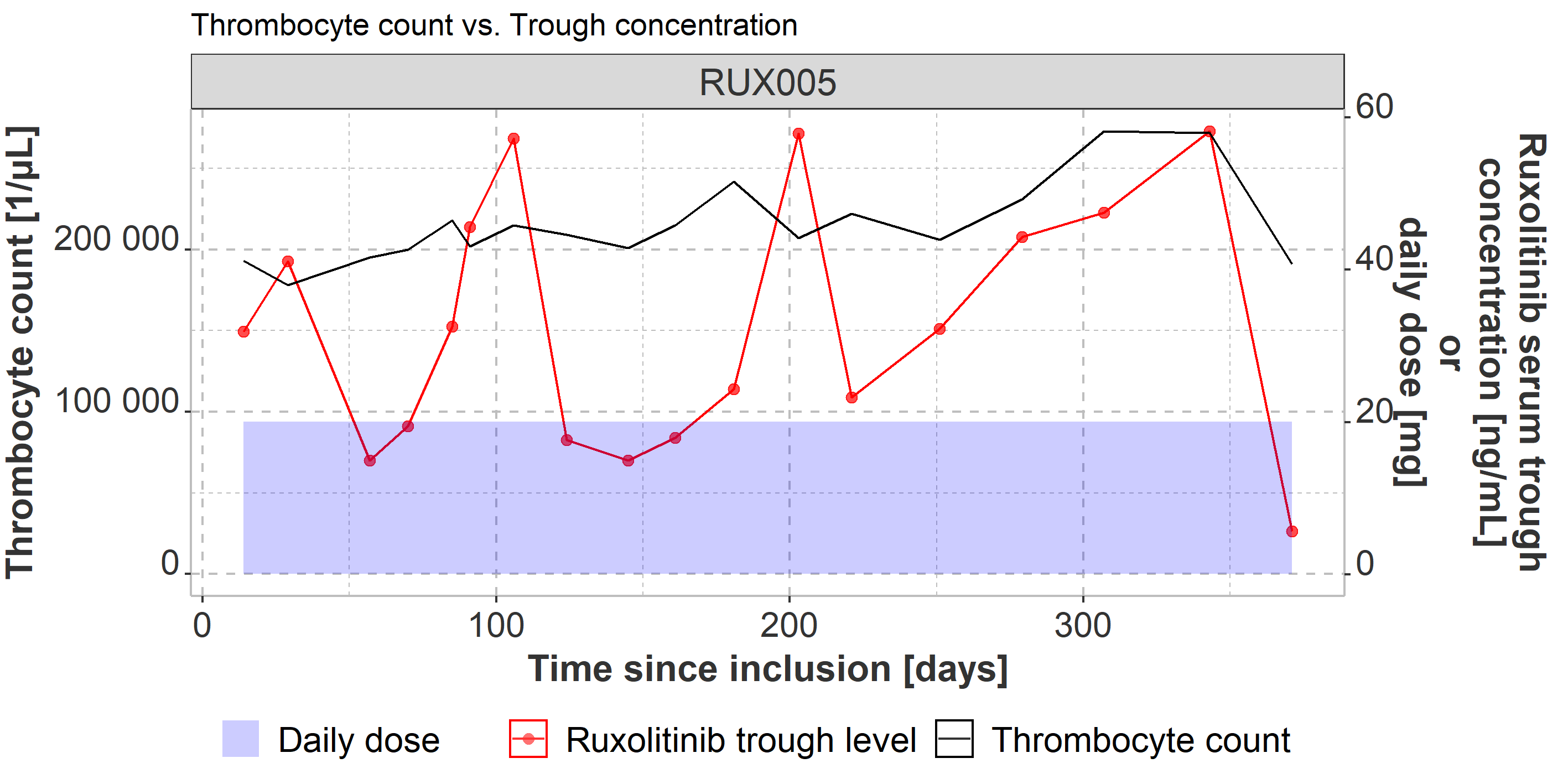 | 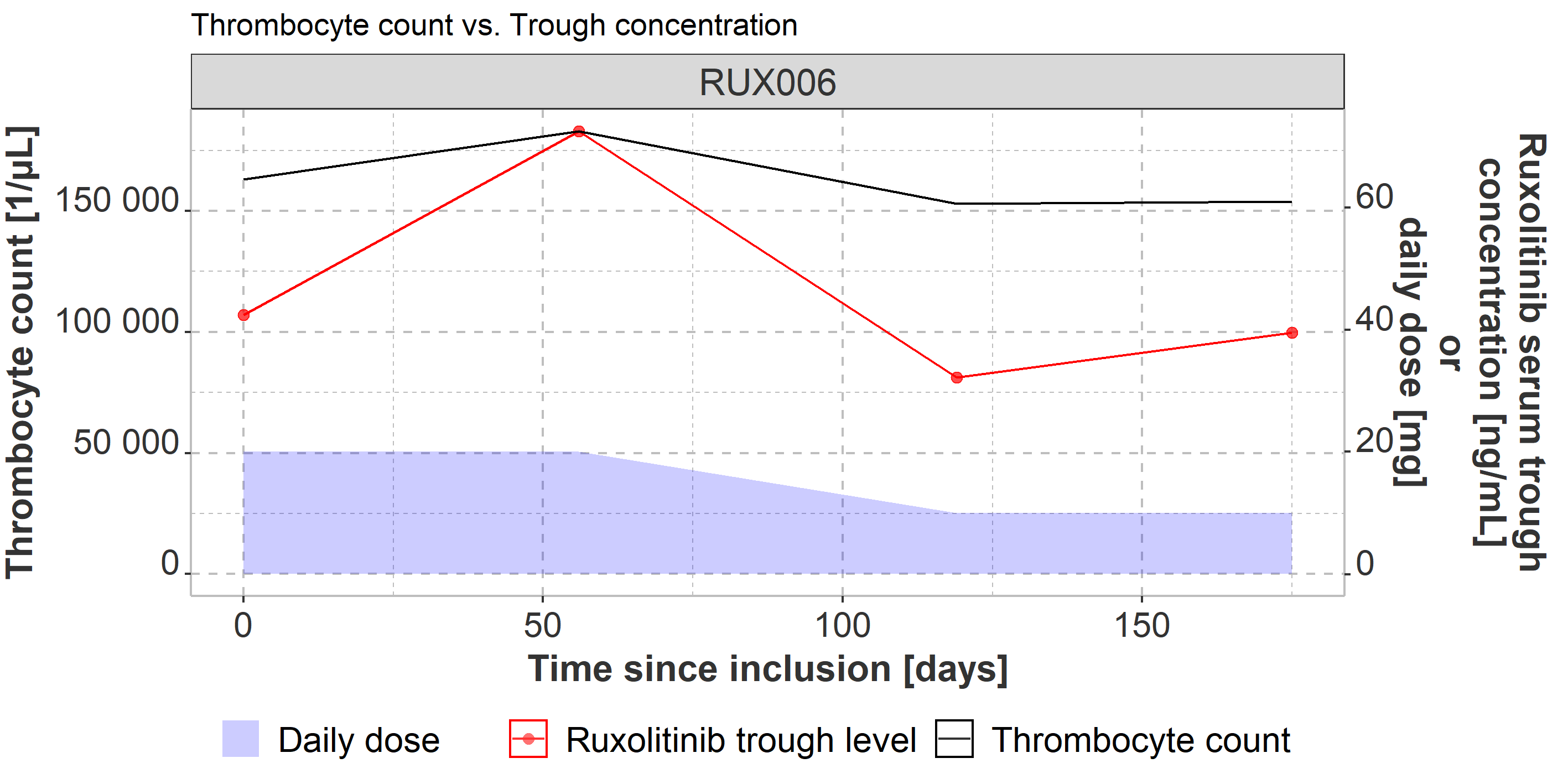 |
| 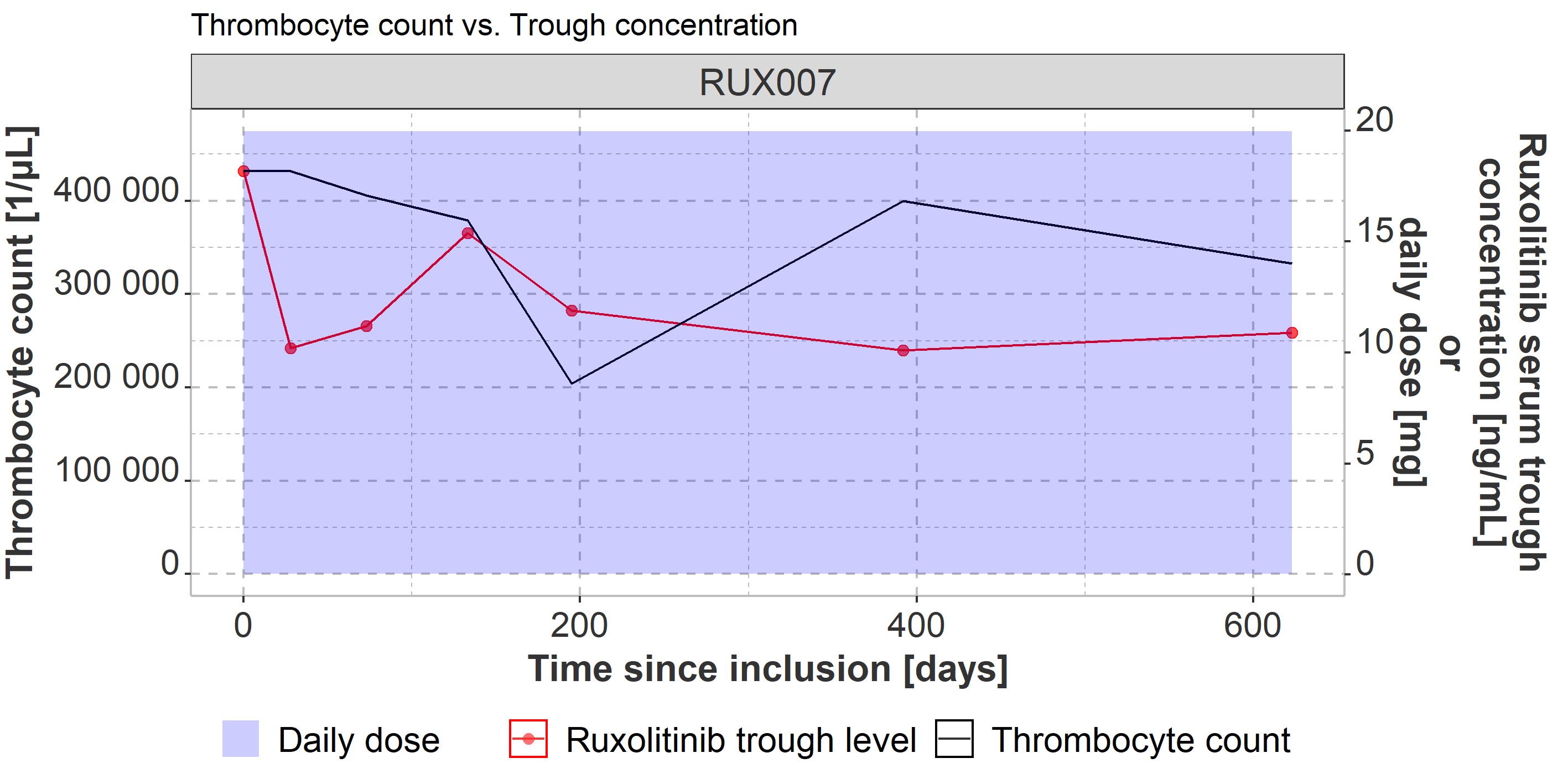 | 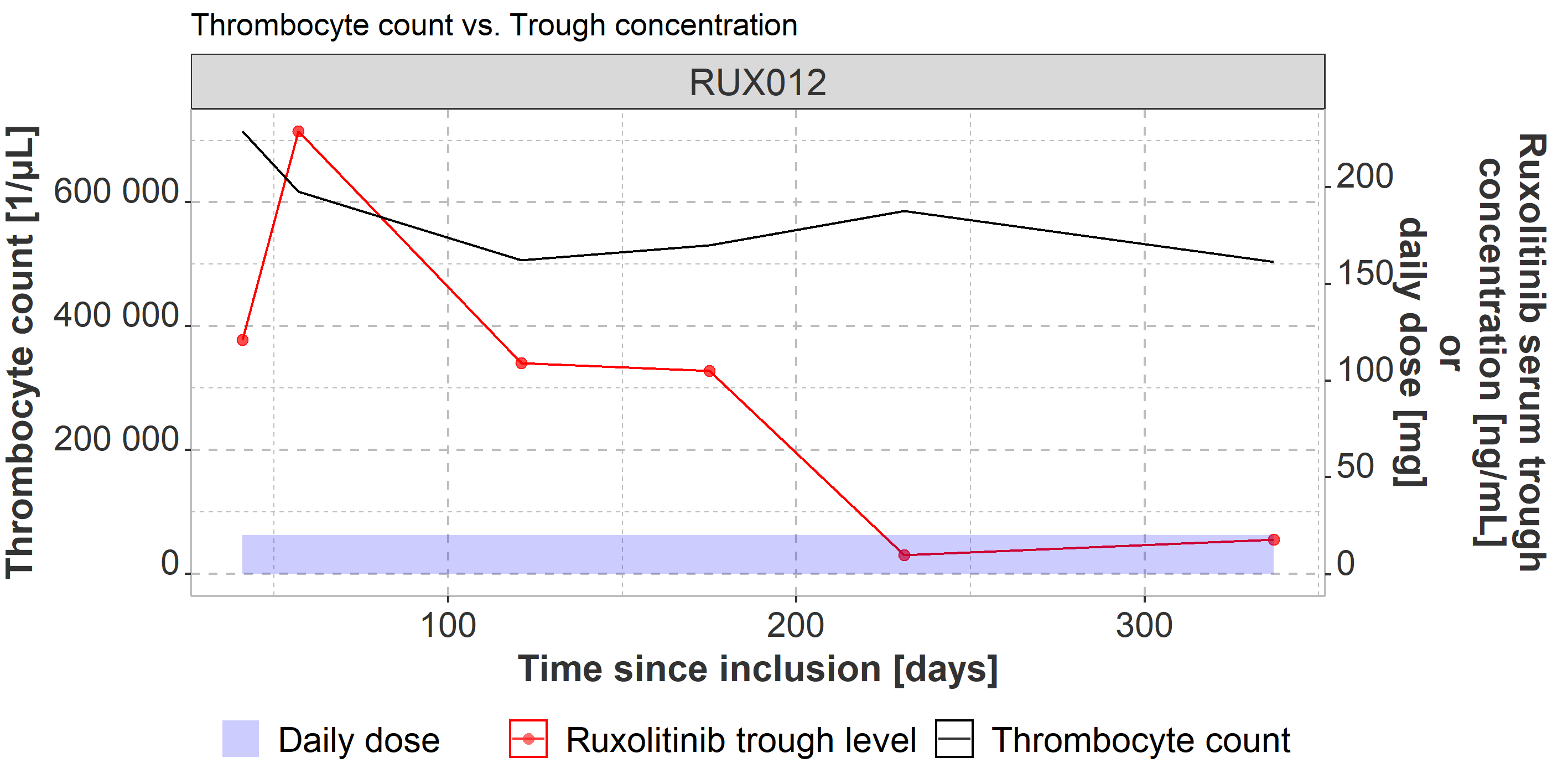 |
| 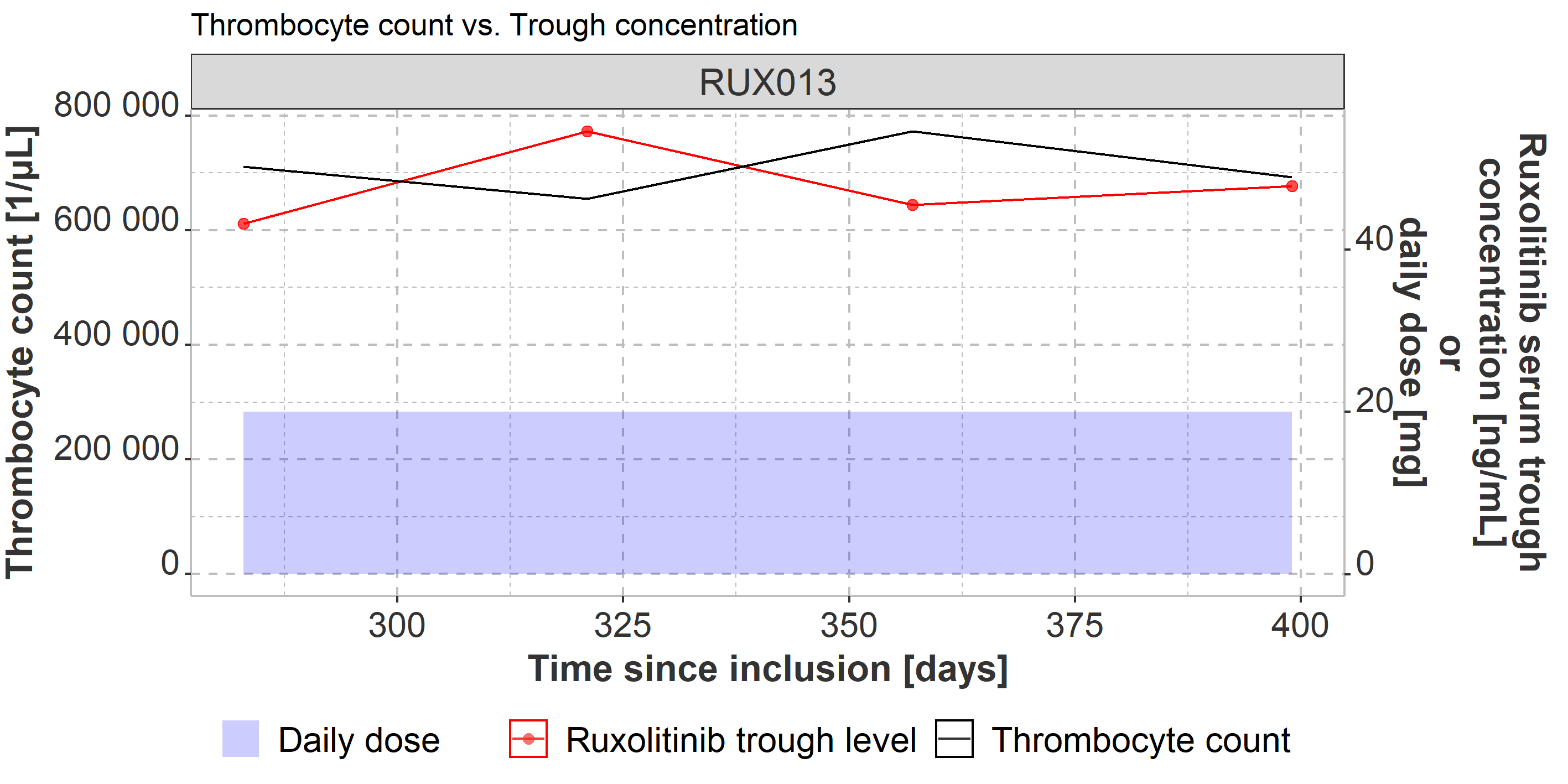 | 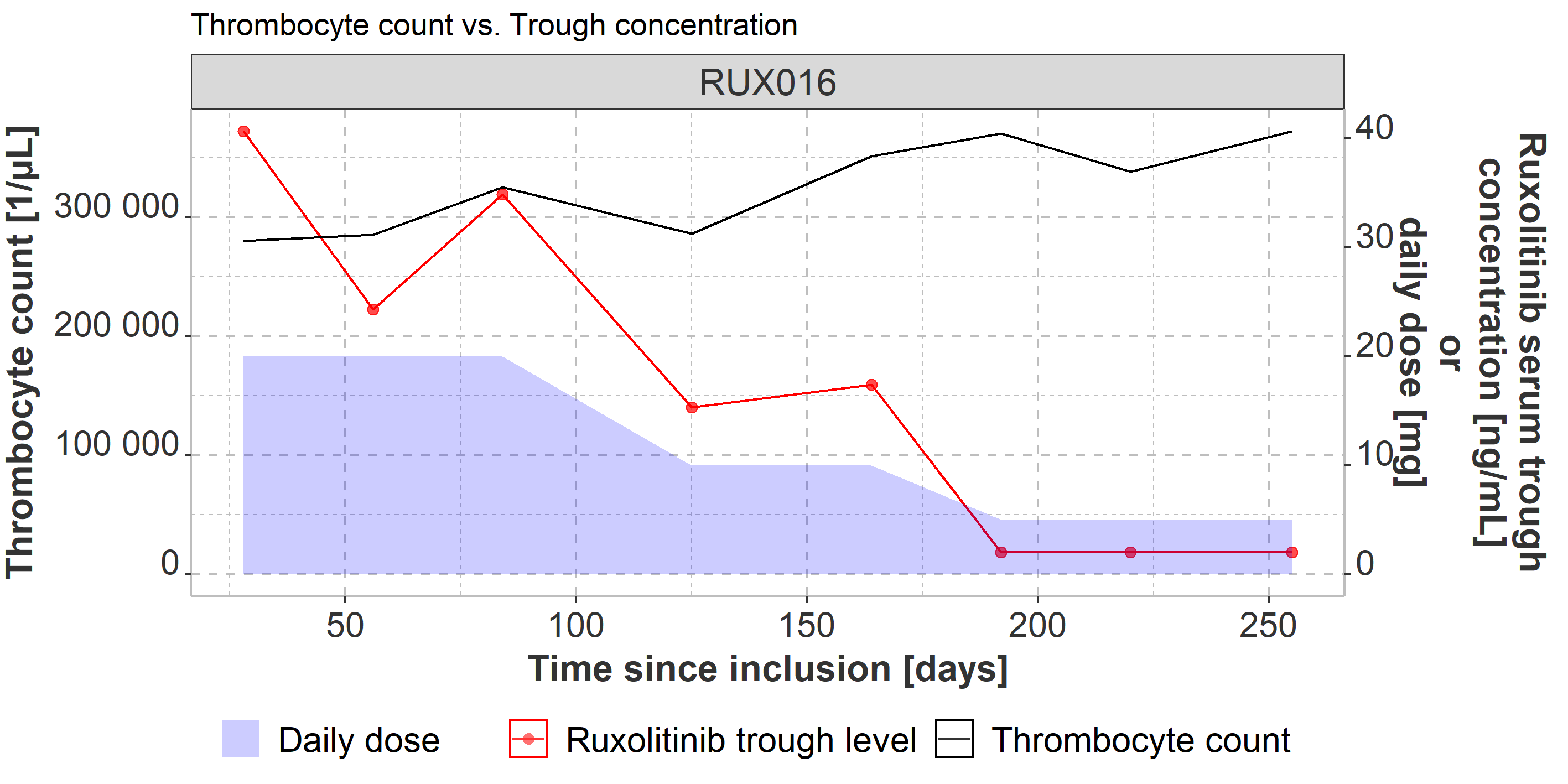 |
| 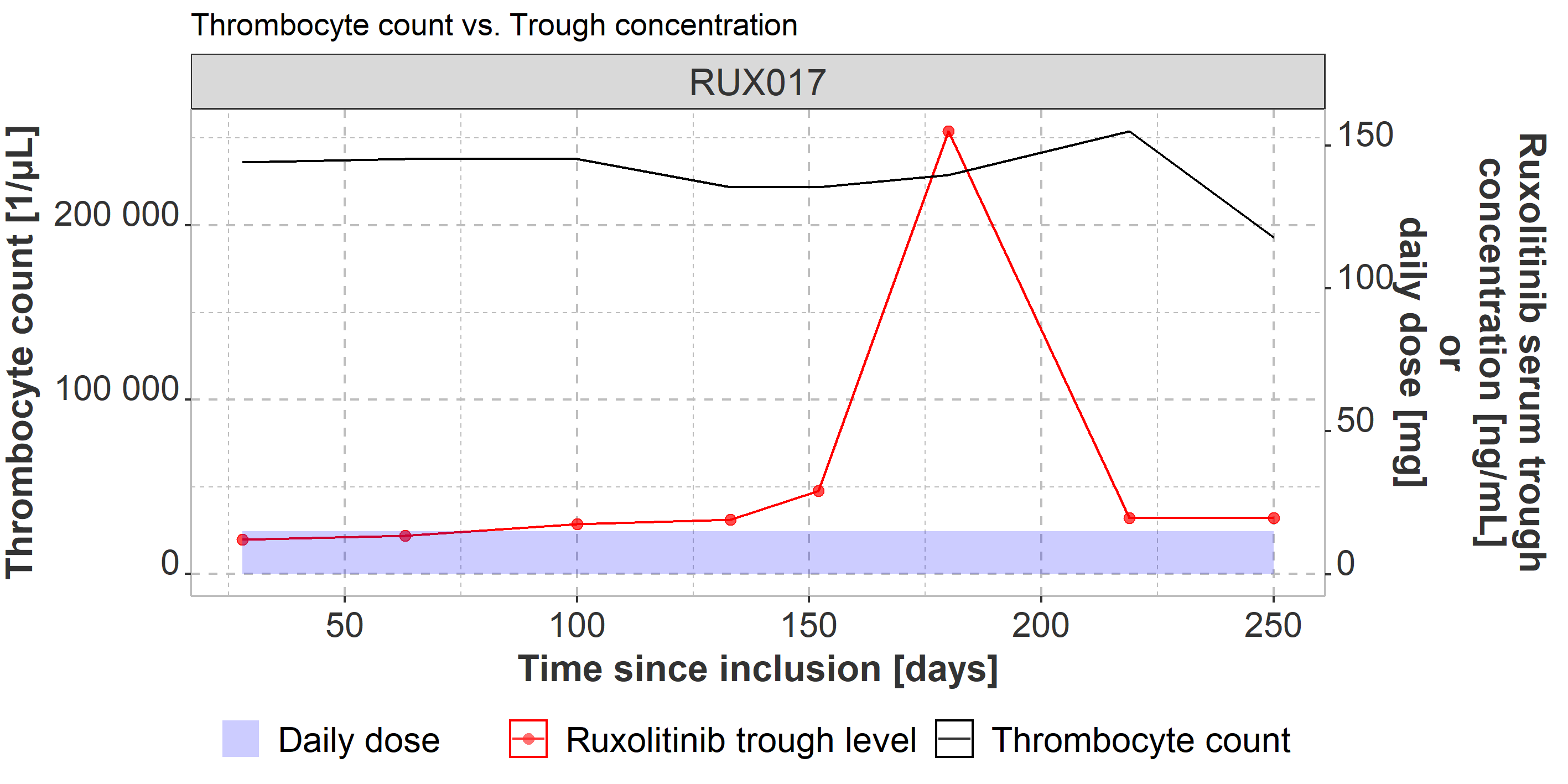 | 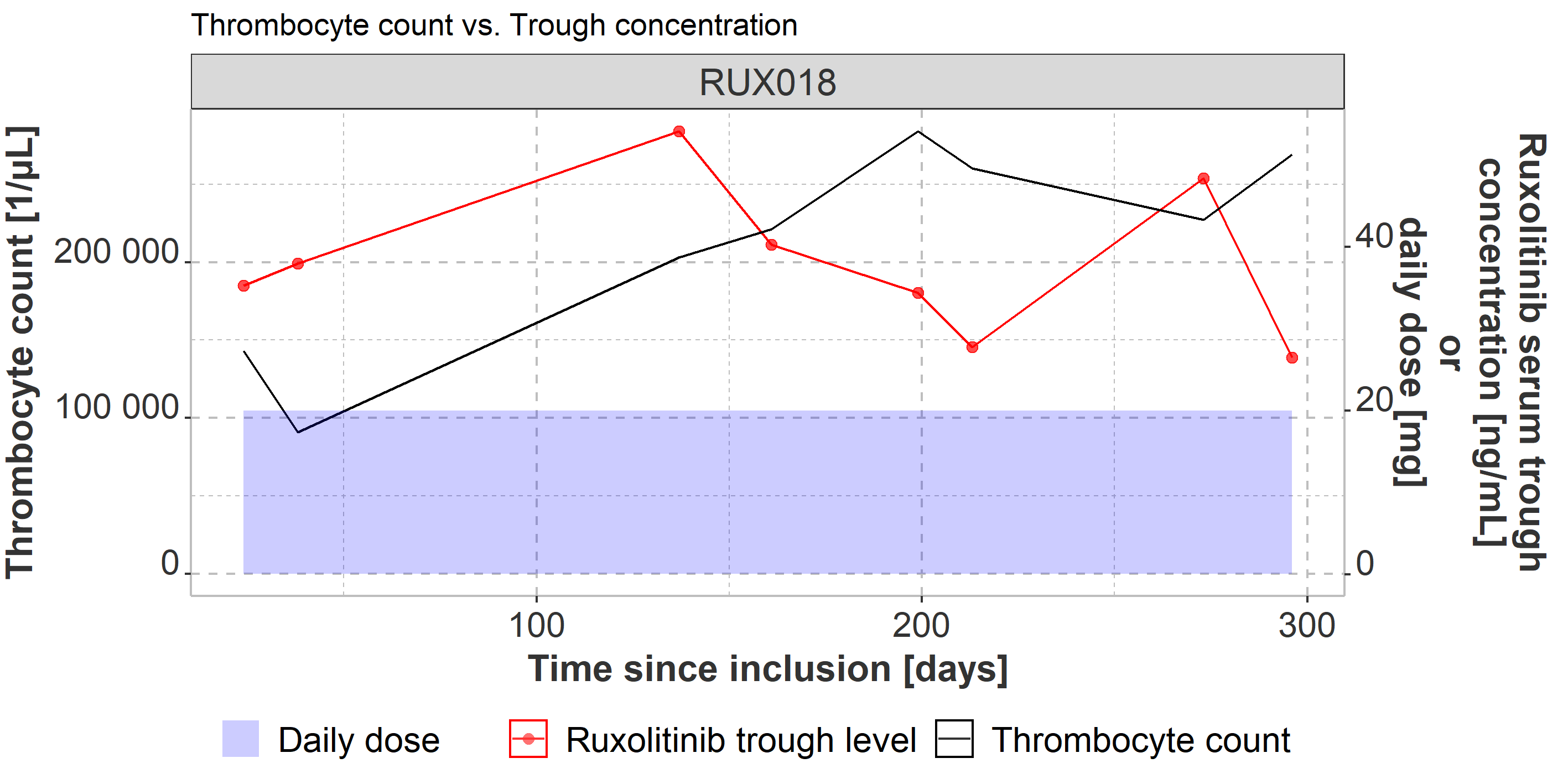 |
| 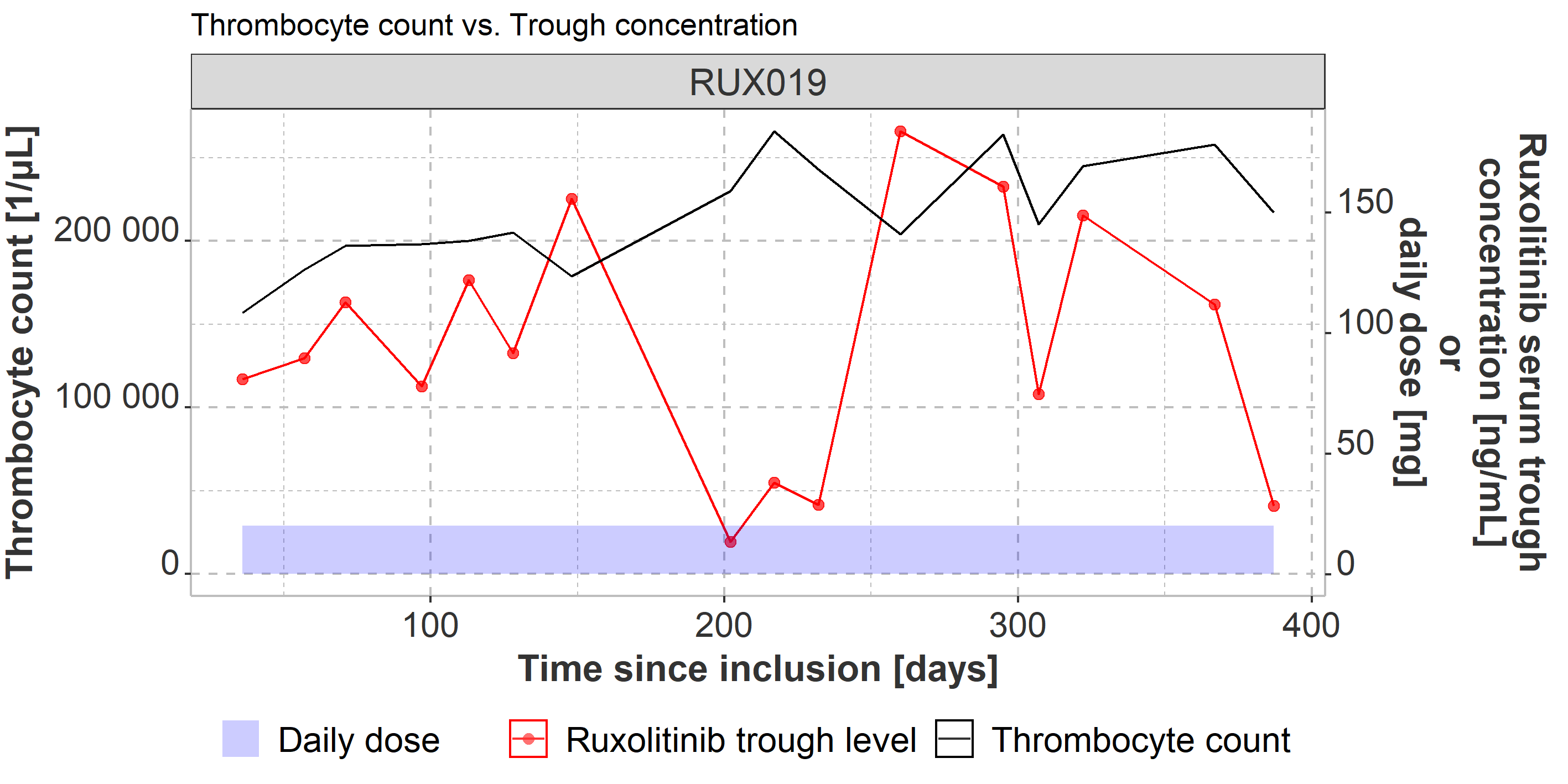 | 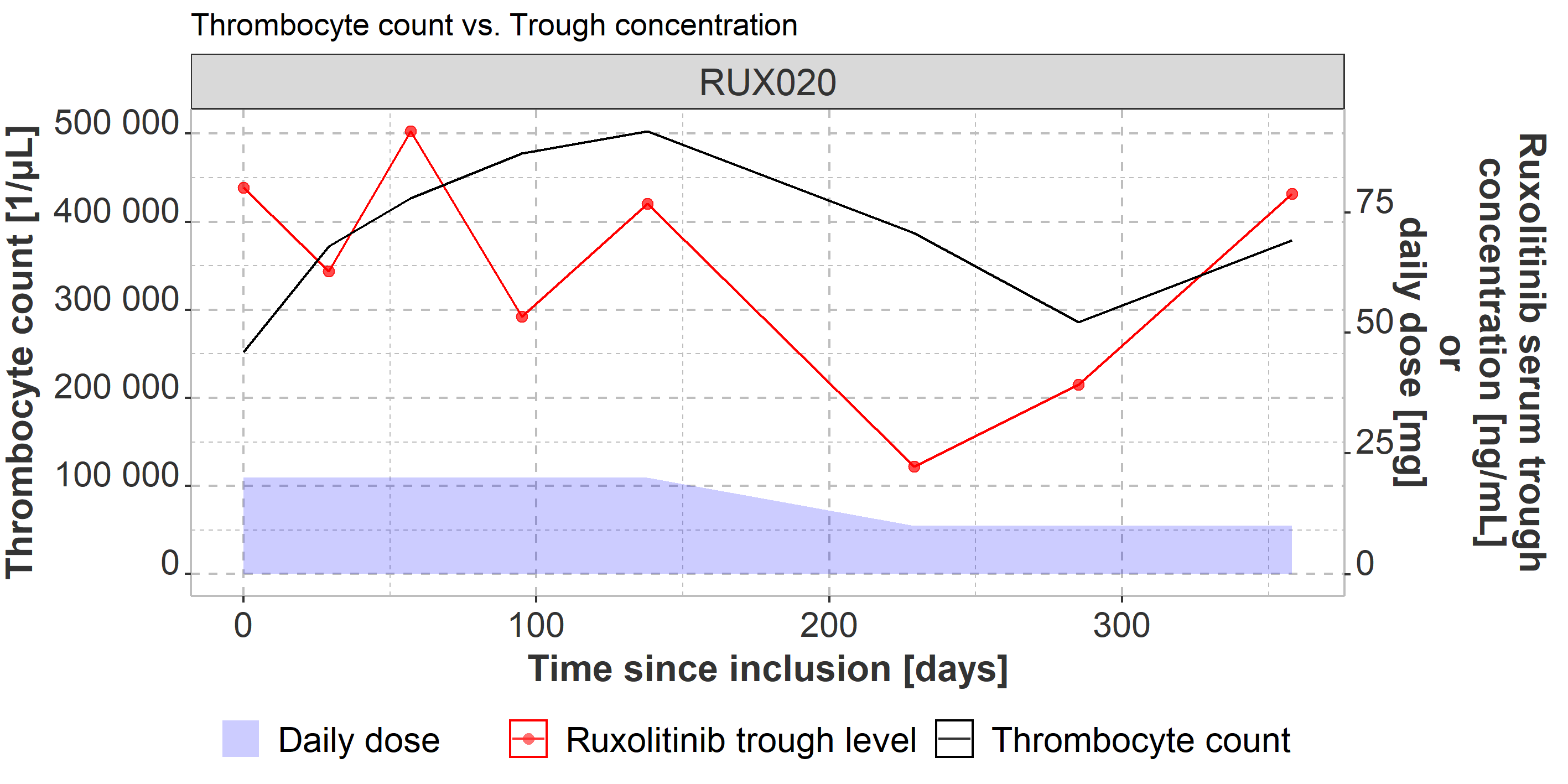 |
| 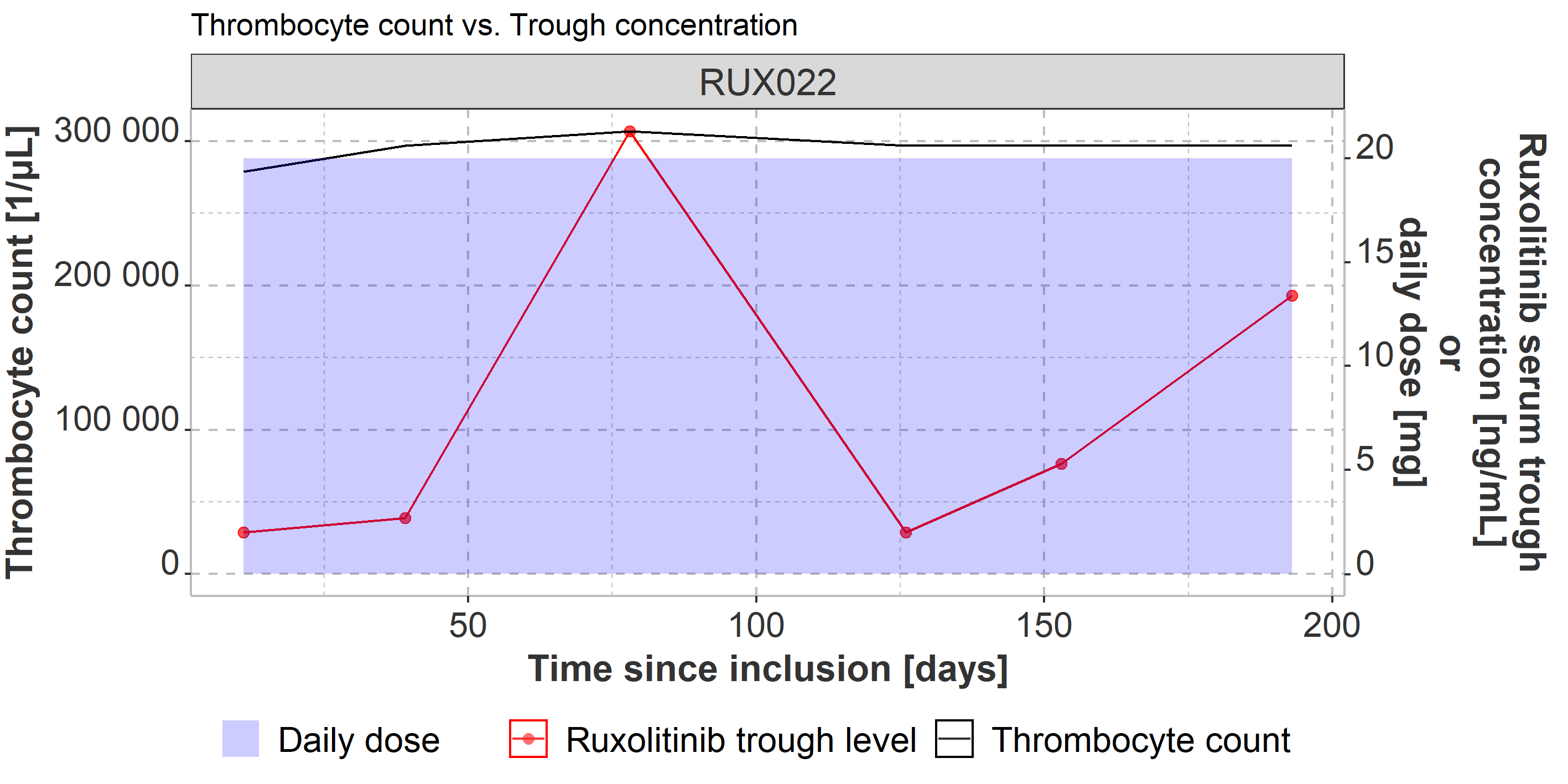 | 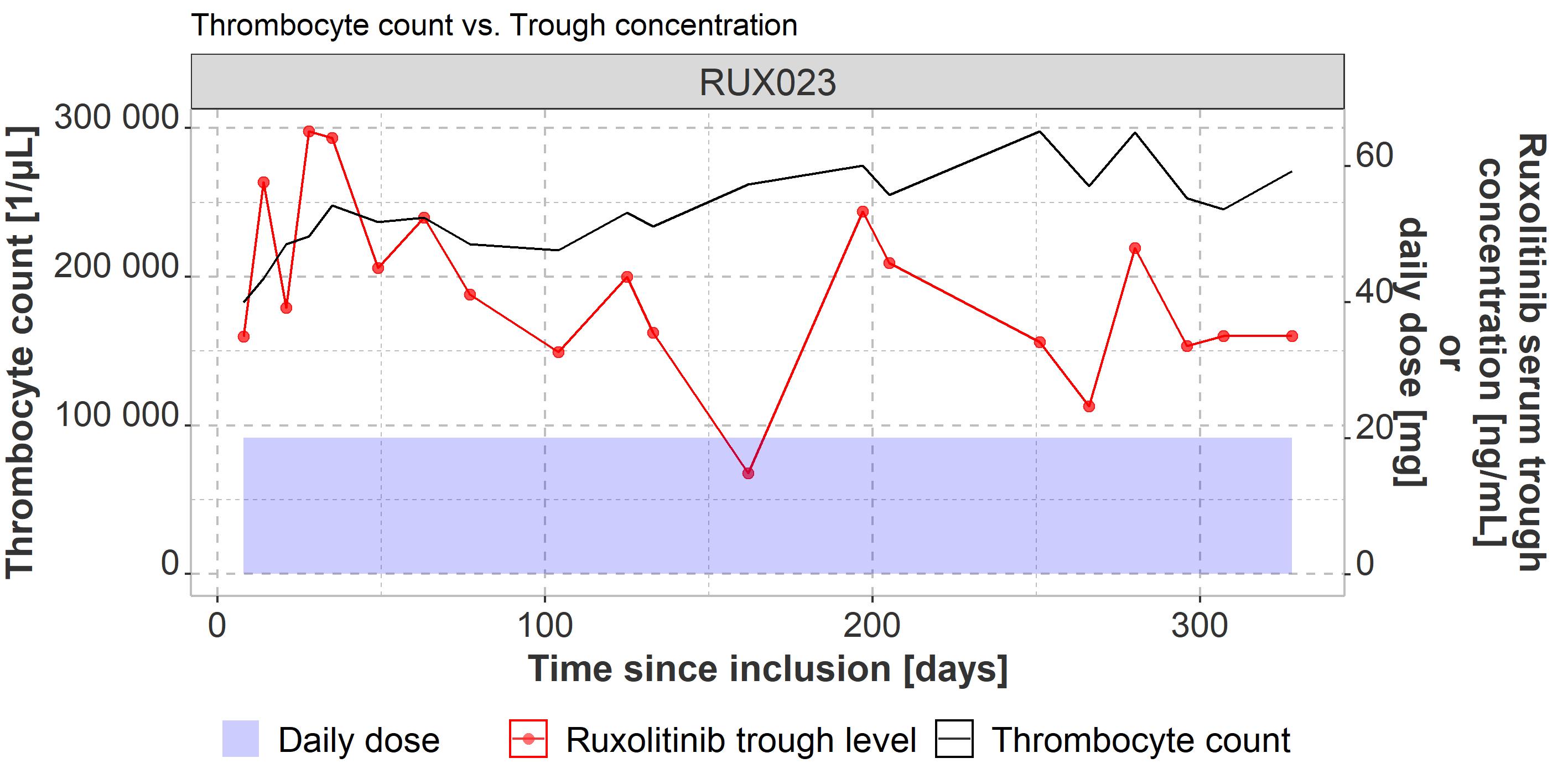 |
| 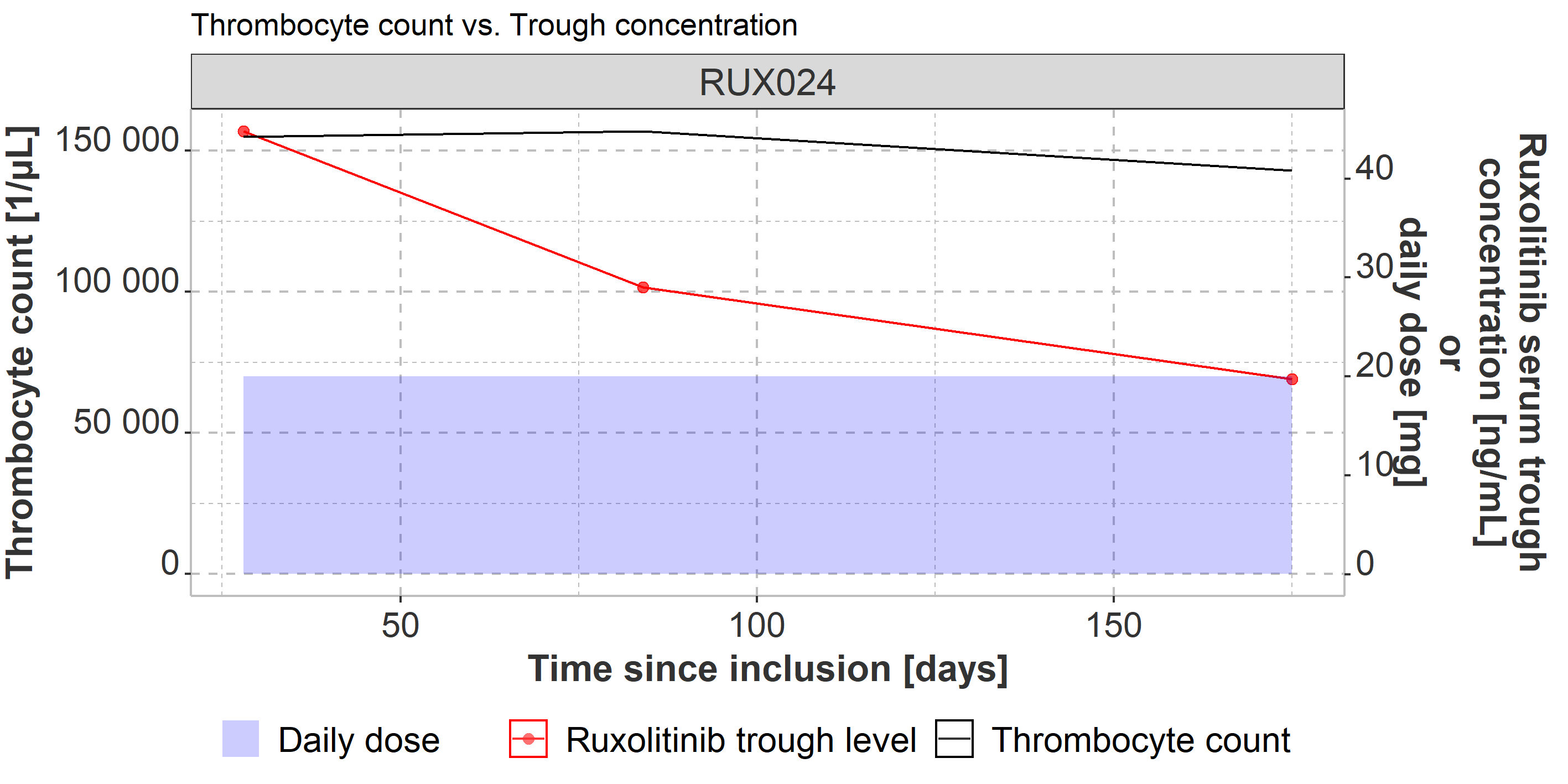 | 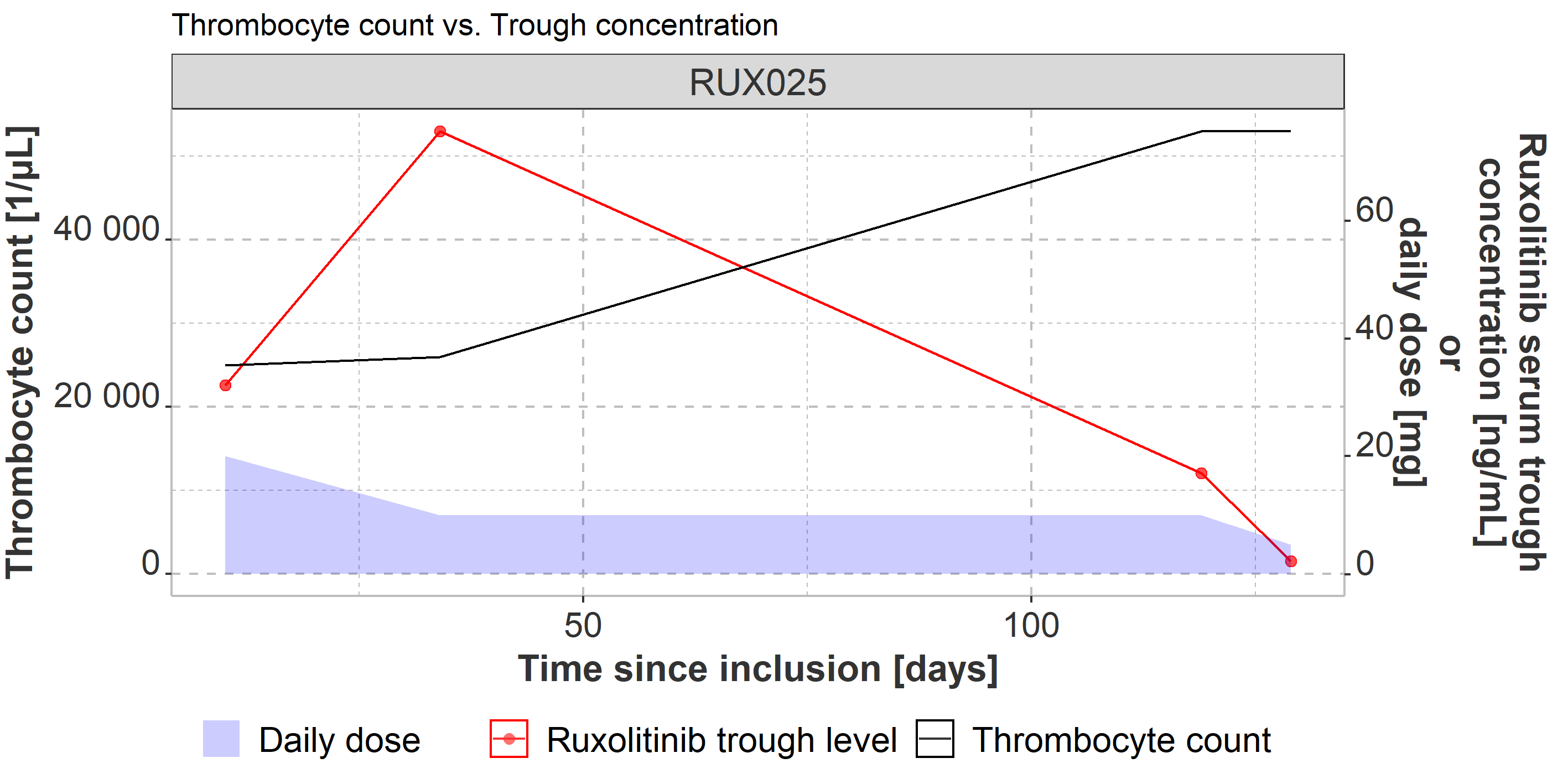 |
| 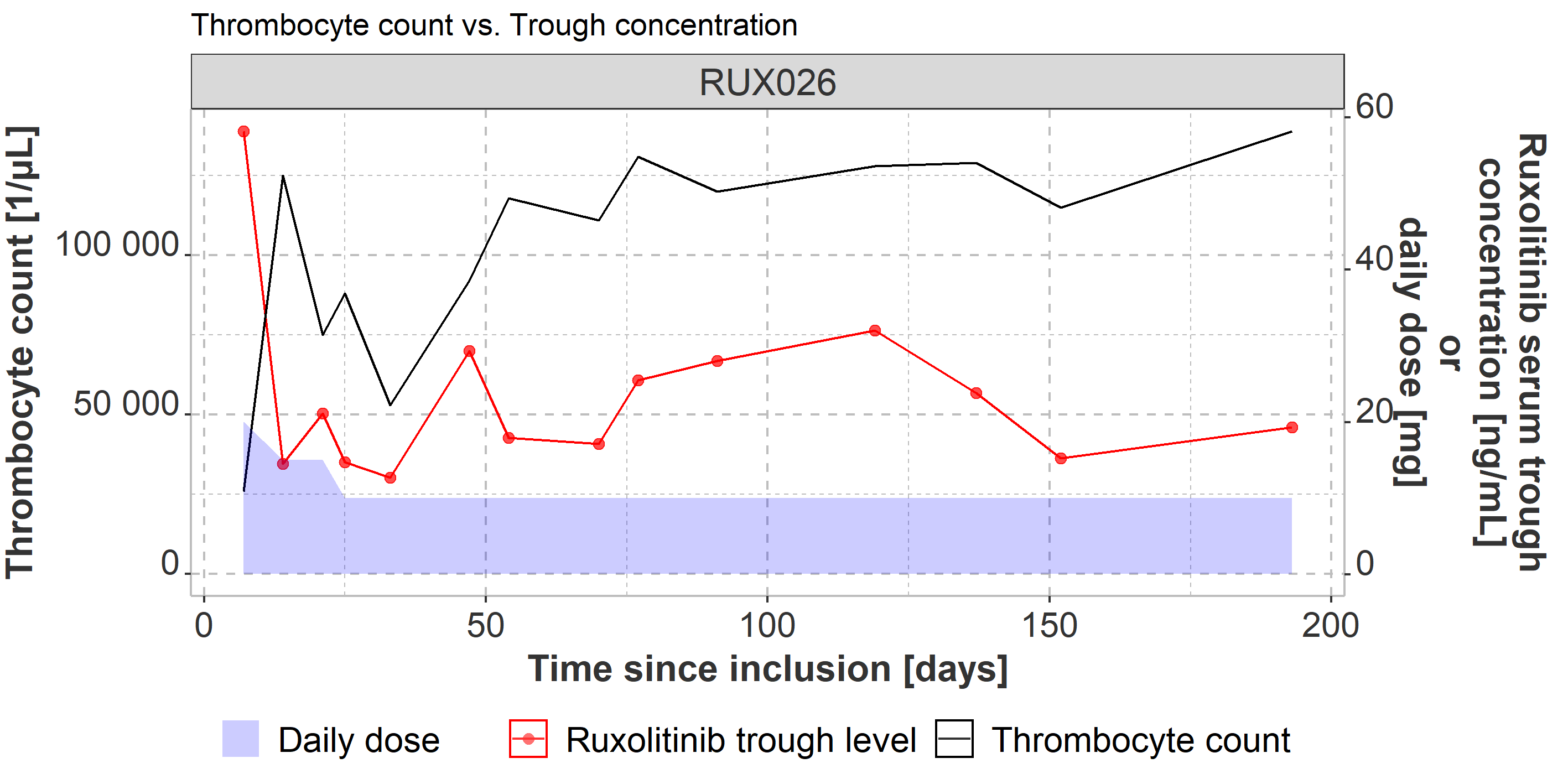 | 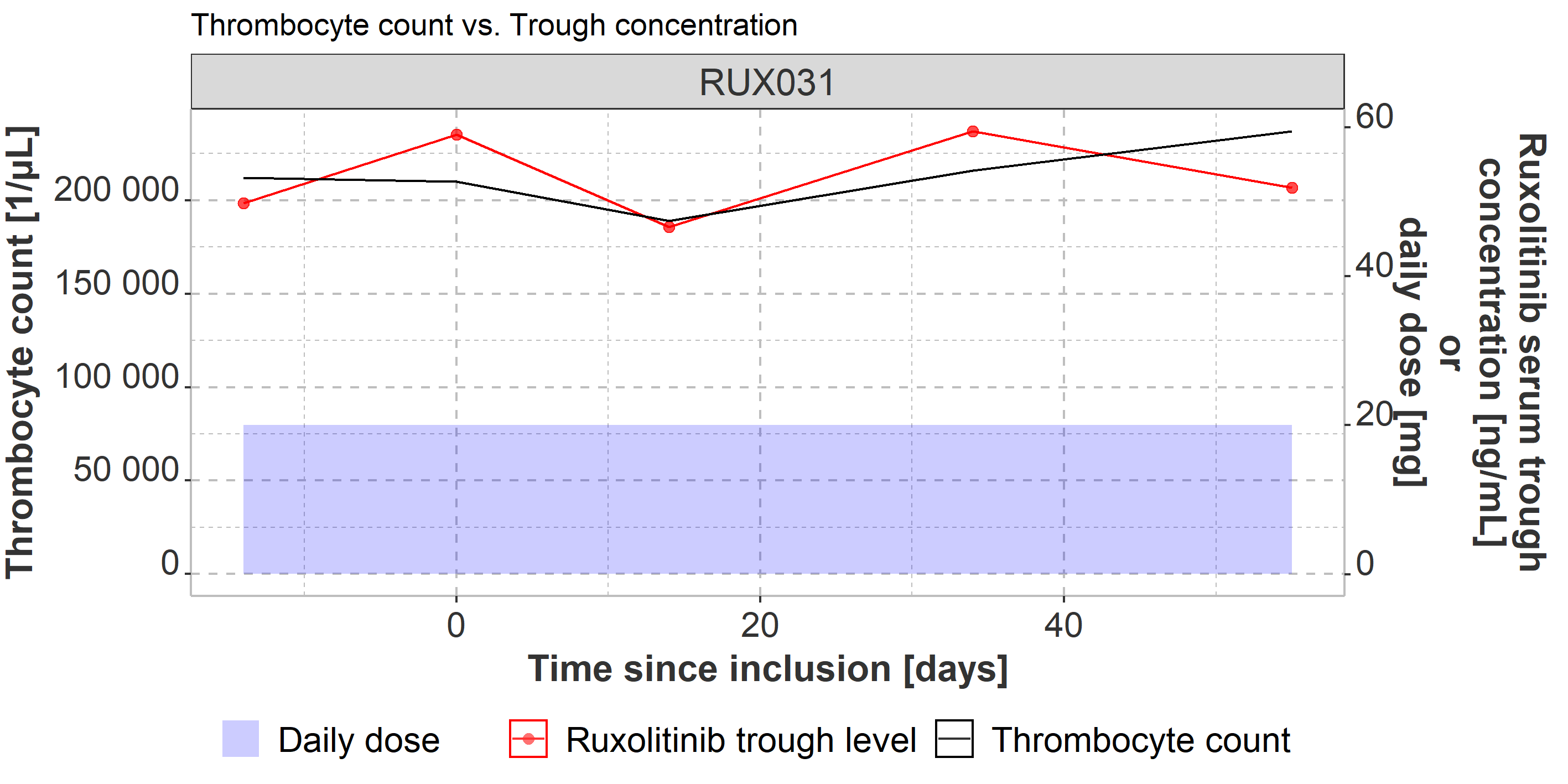 |

| **Supplementary Fig. 7.** Absolute neutrophil count vs. Ruxolitinib trough serum concentration | |
| --- | --- |
| 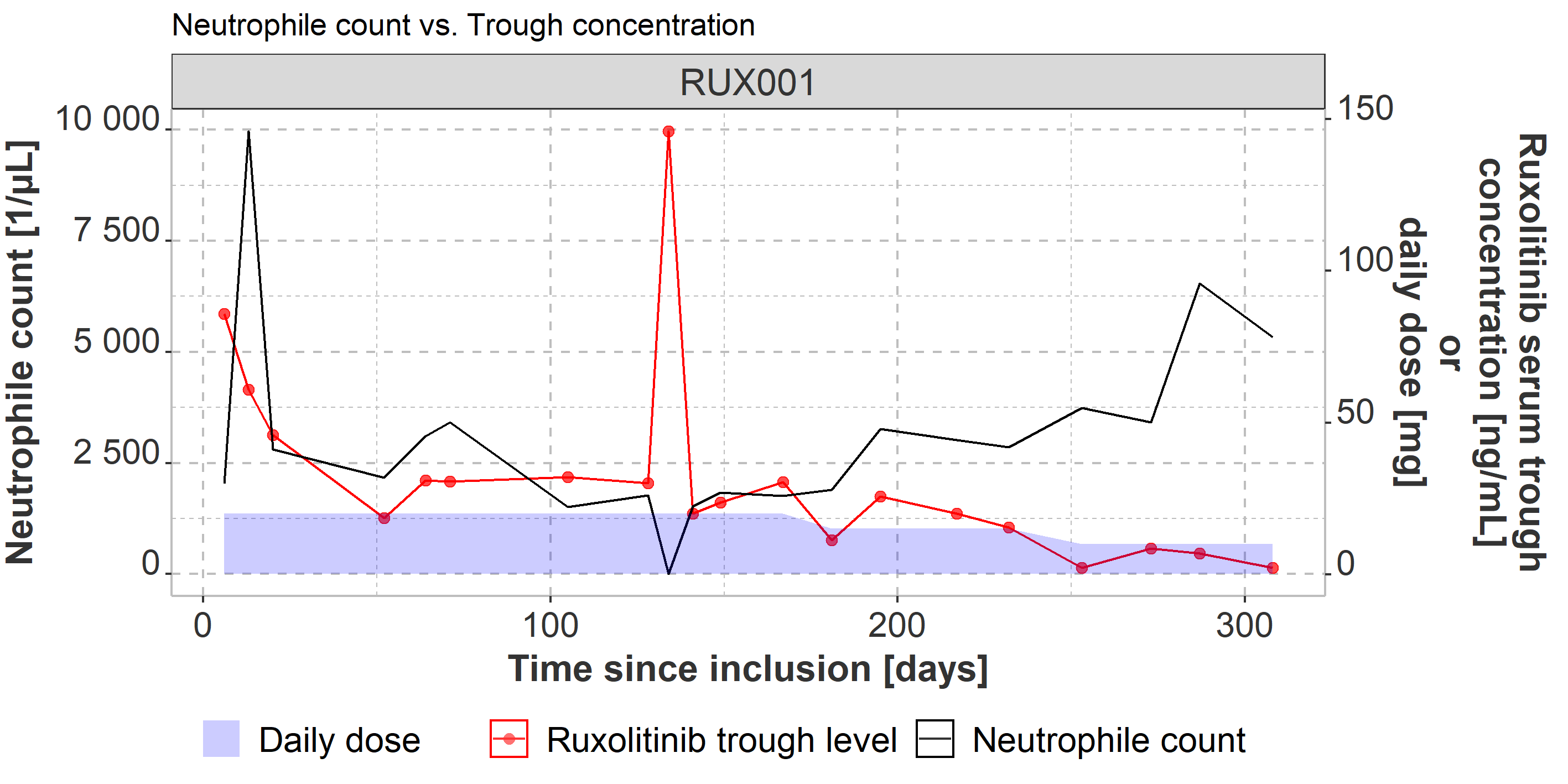 | 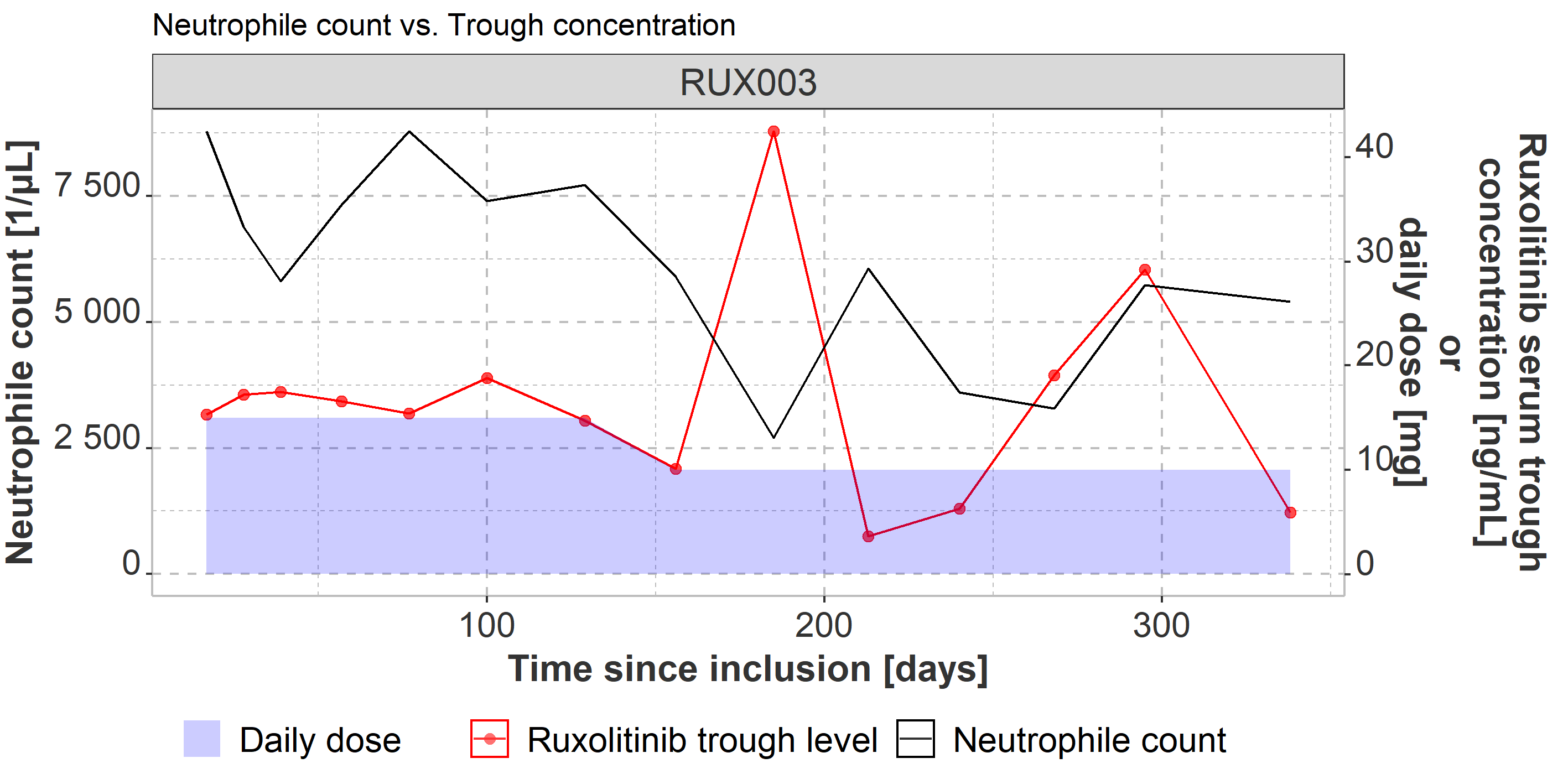 |
| 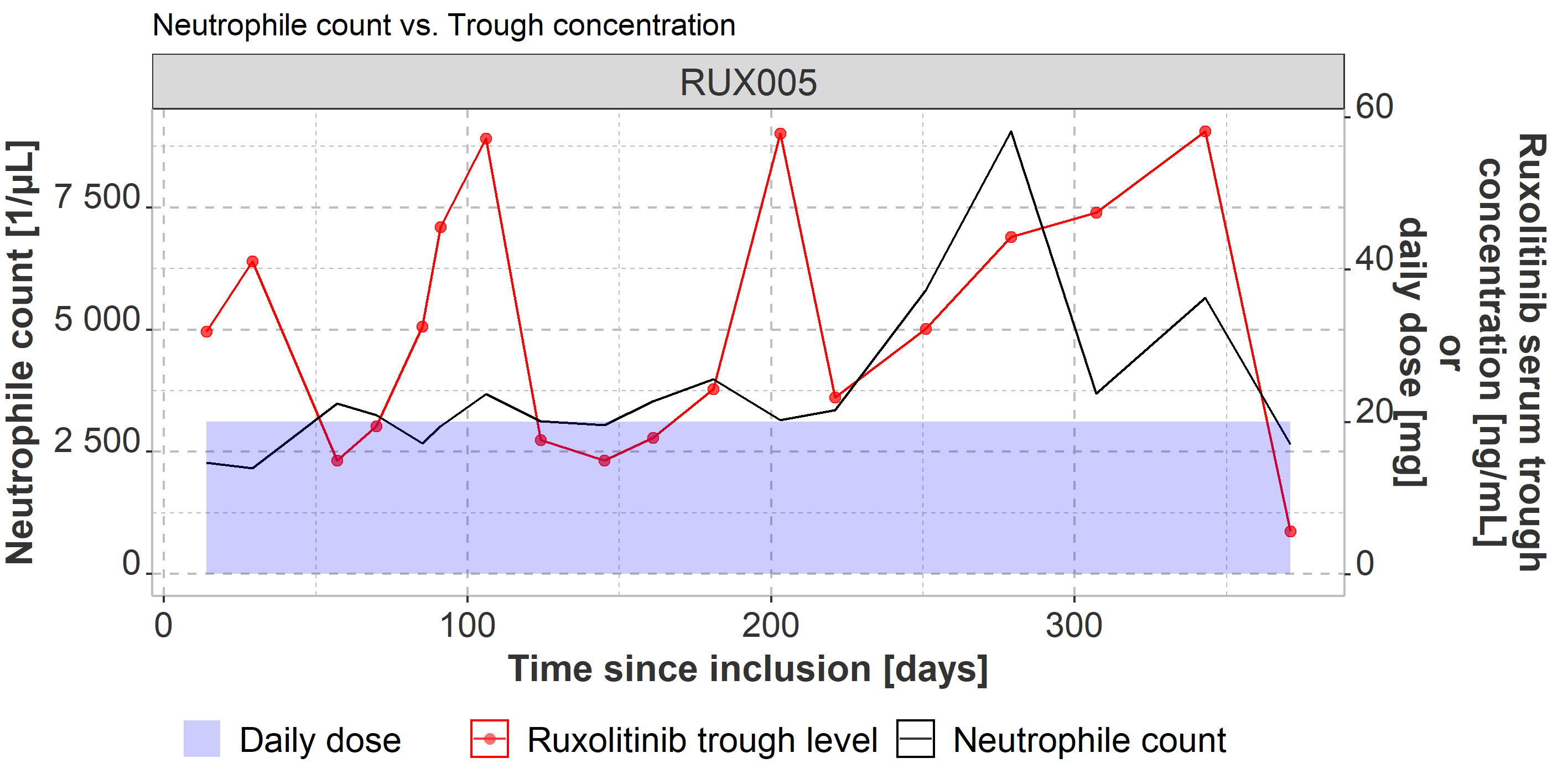 | 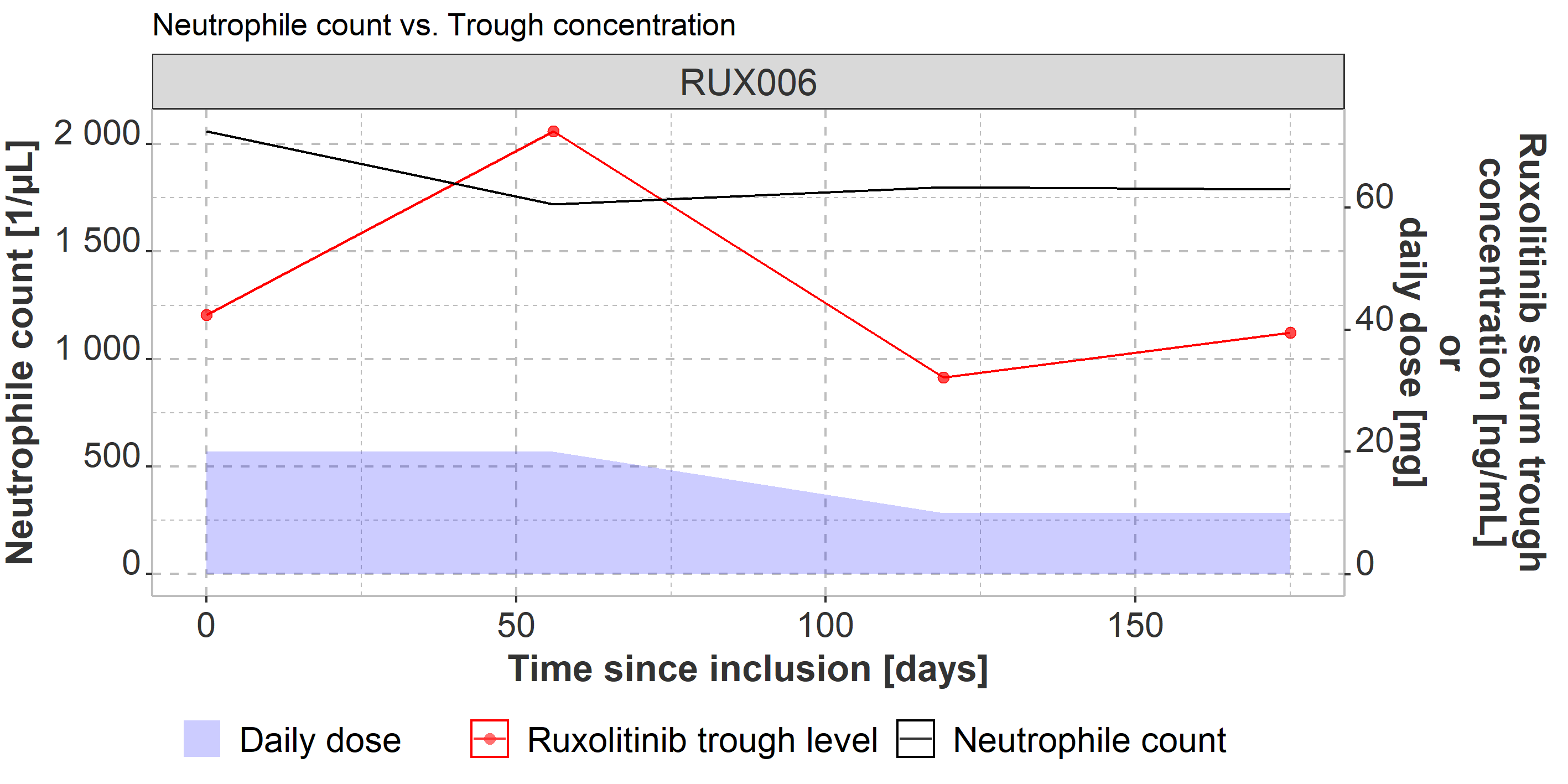 |
| 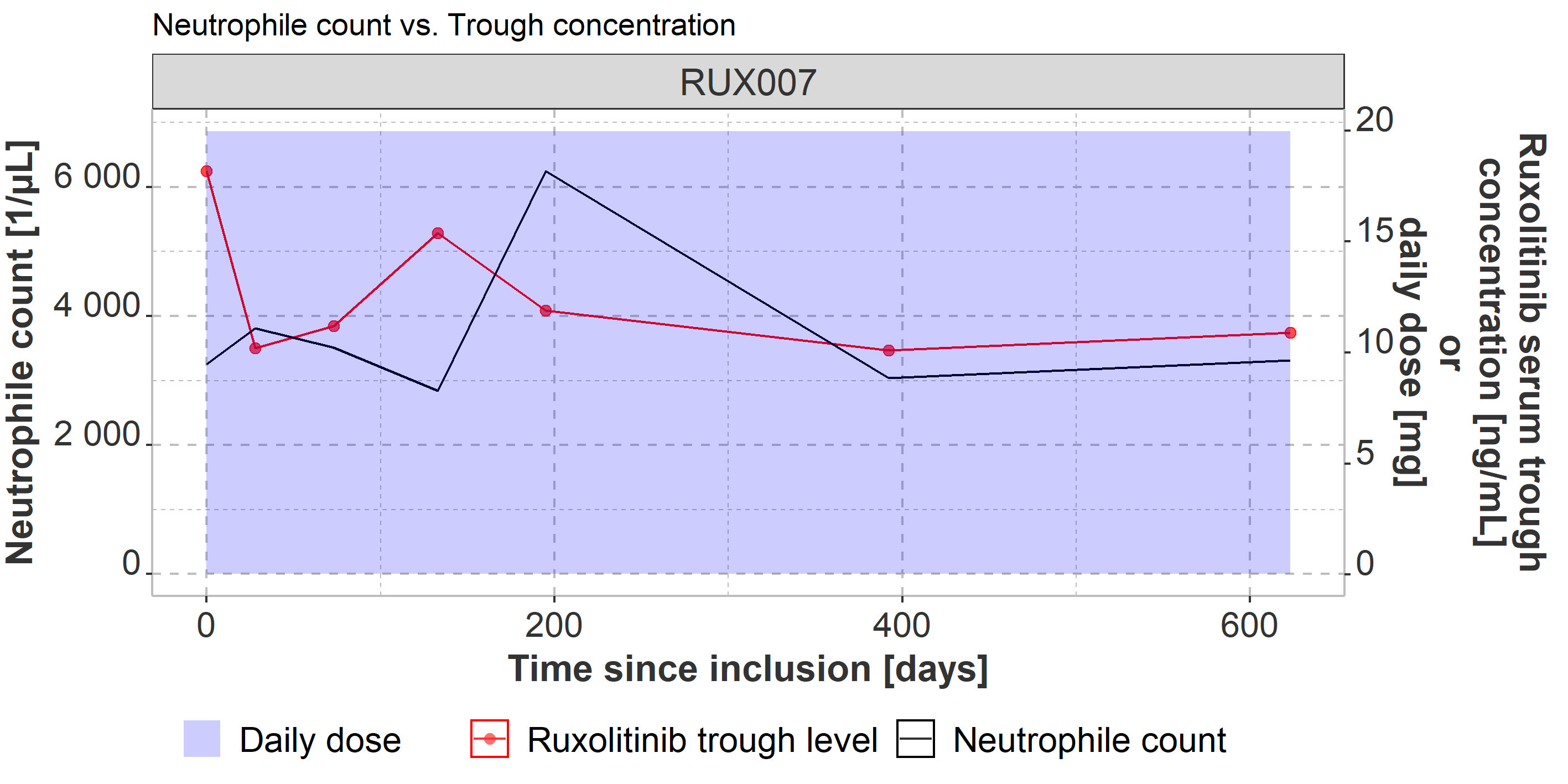 | 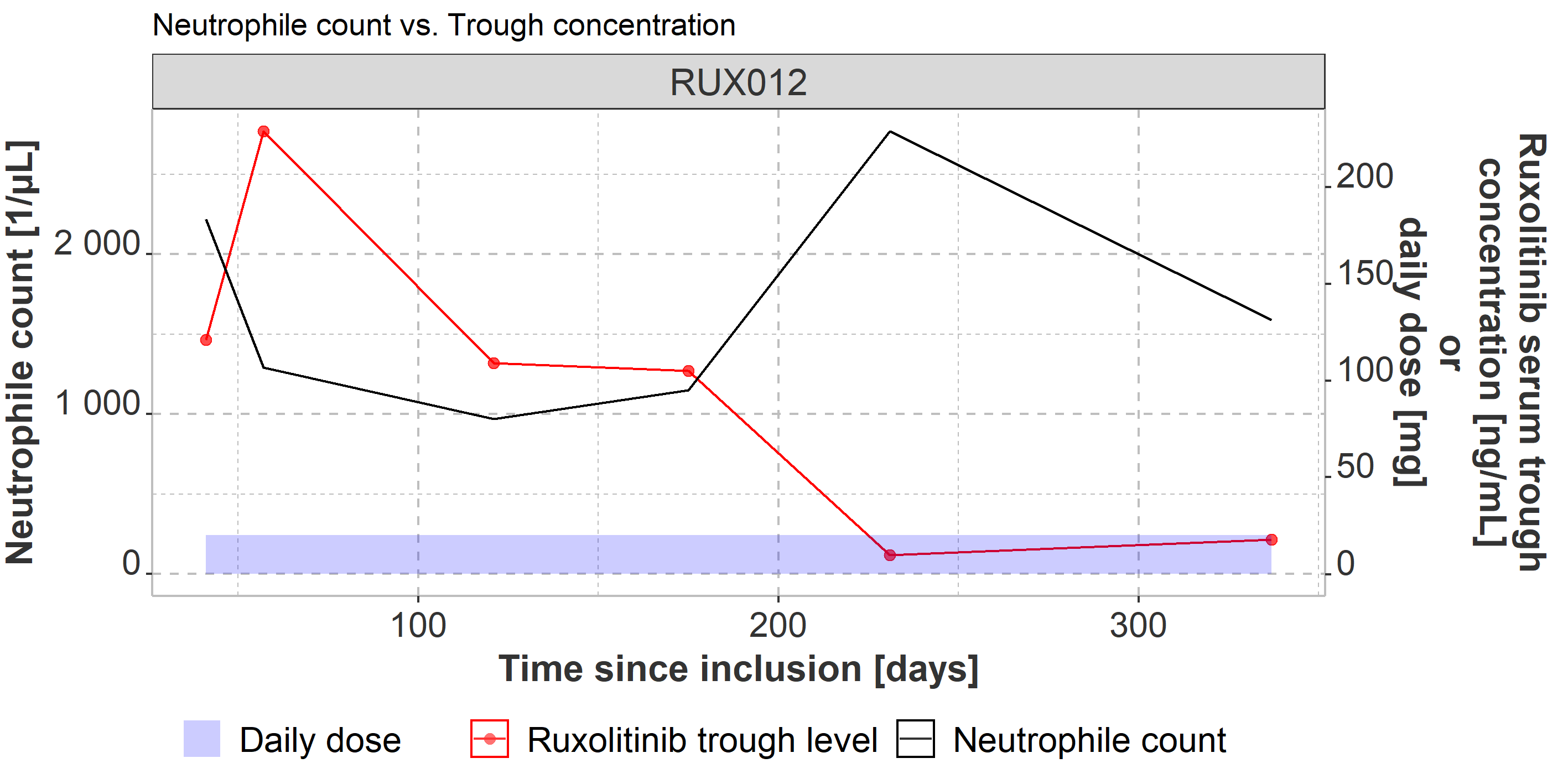 |
| 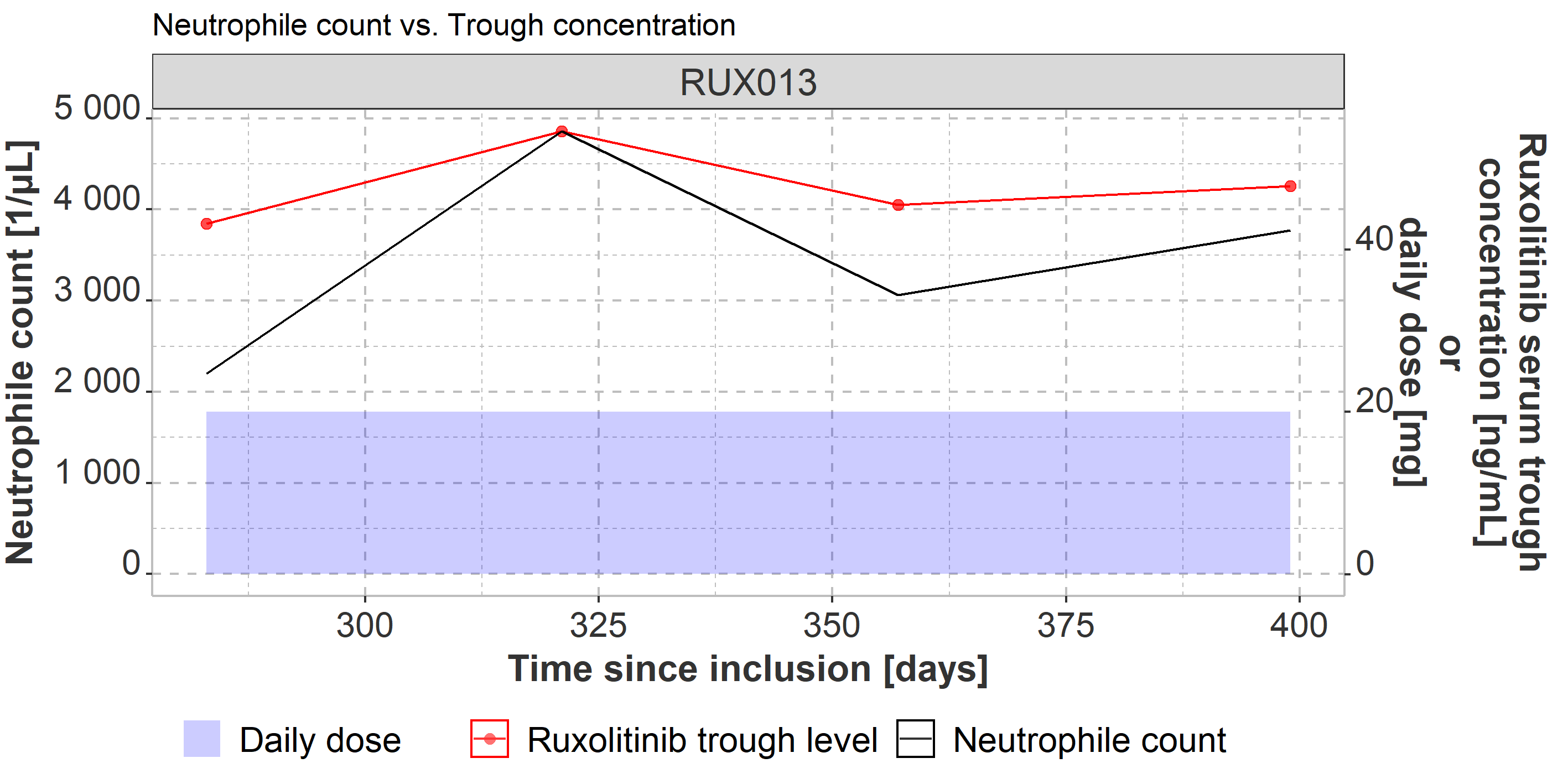 | 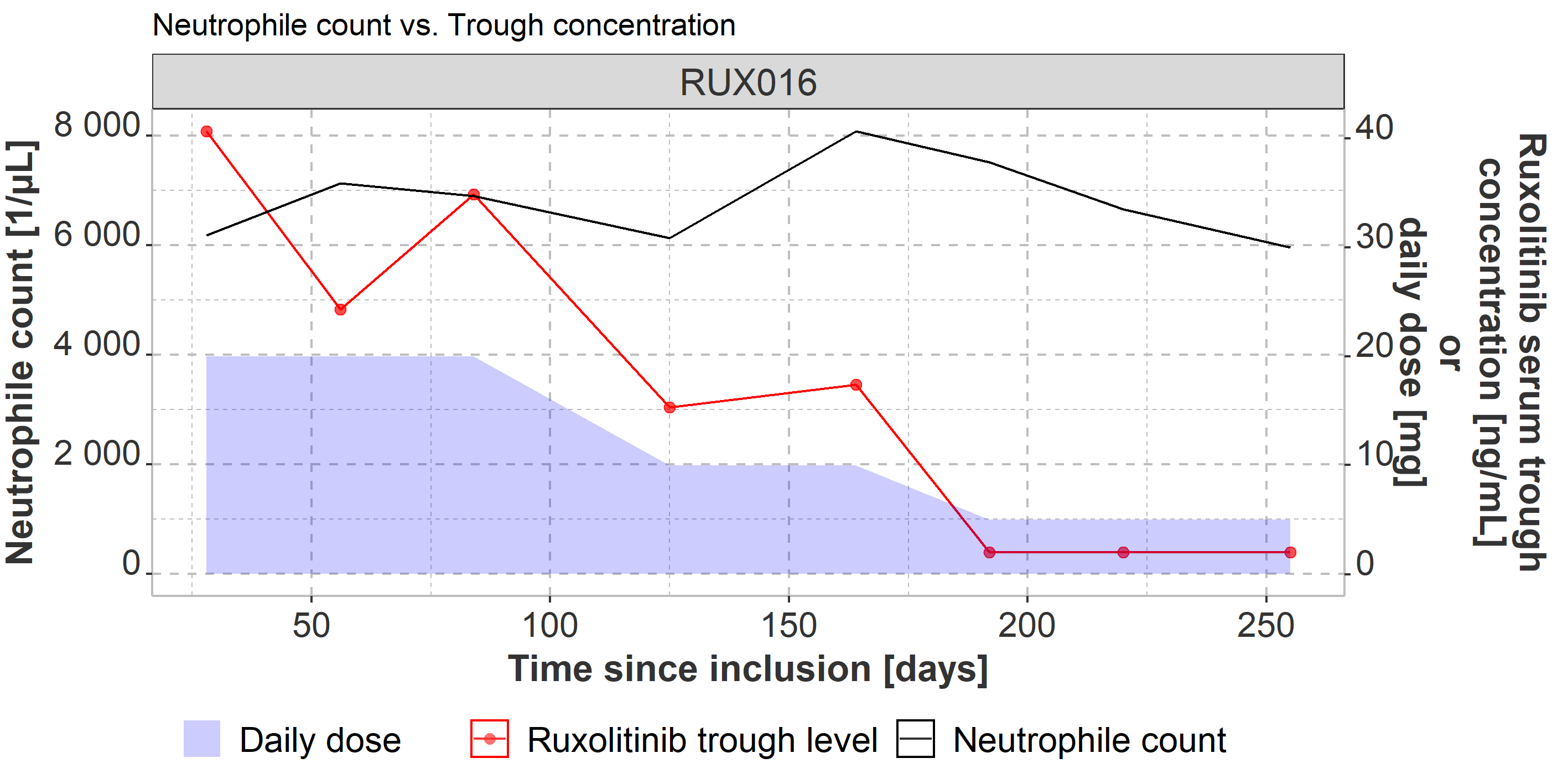 |
| 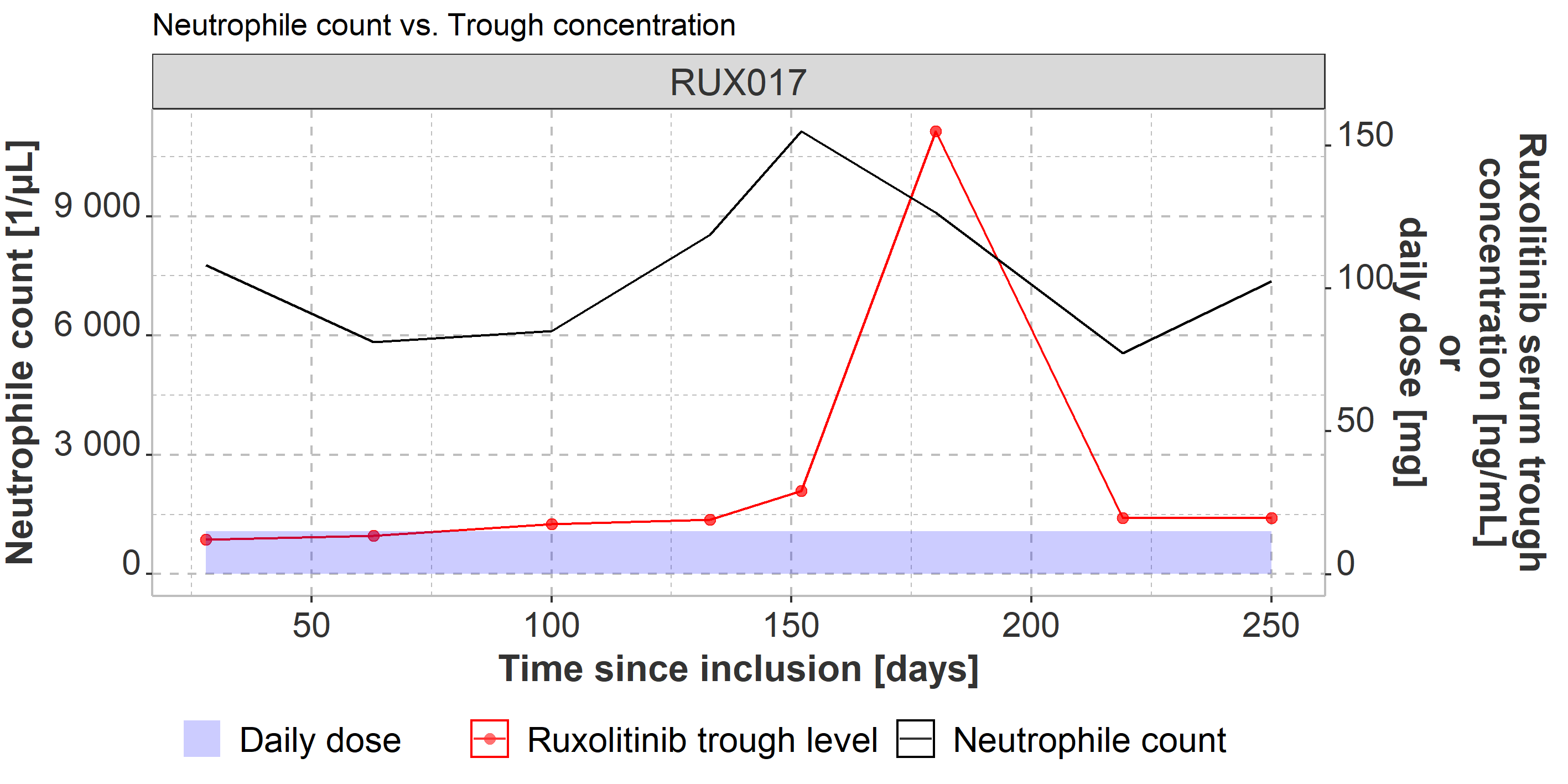 | 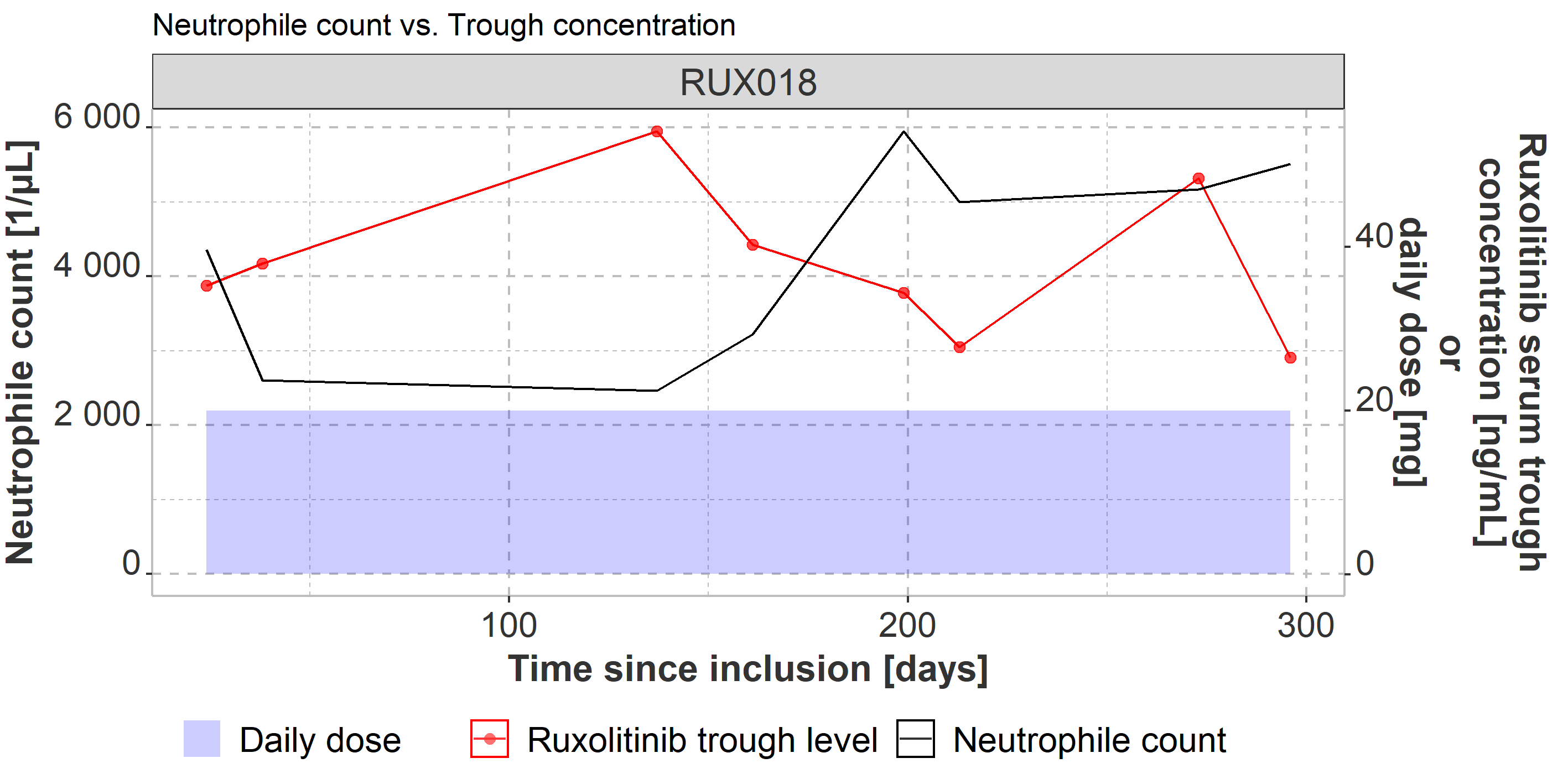 |
| 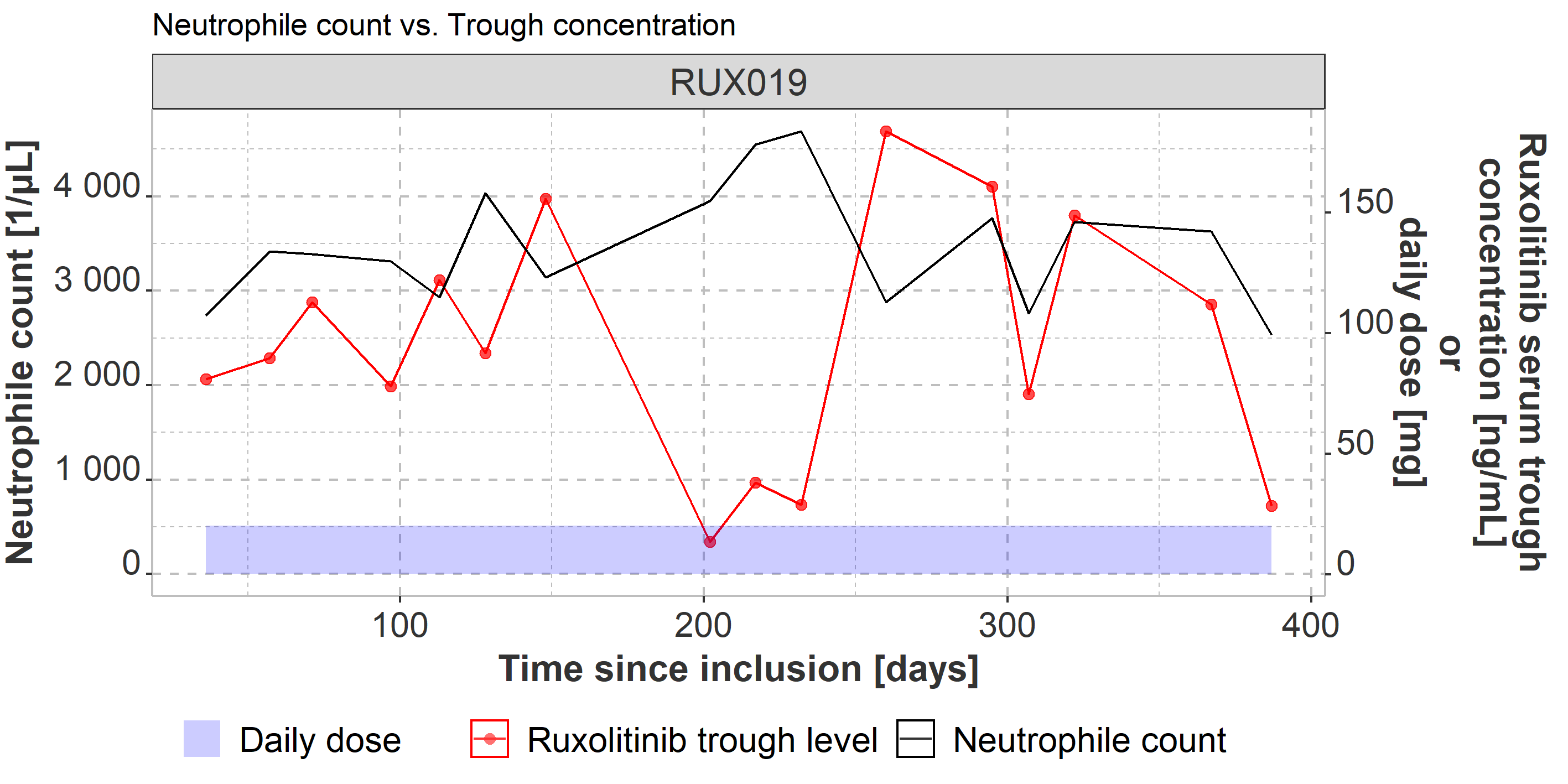 | 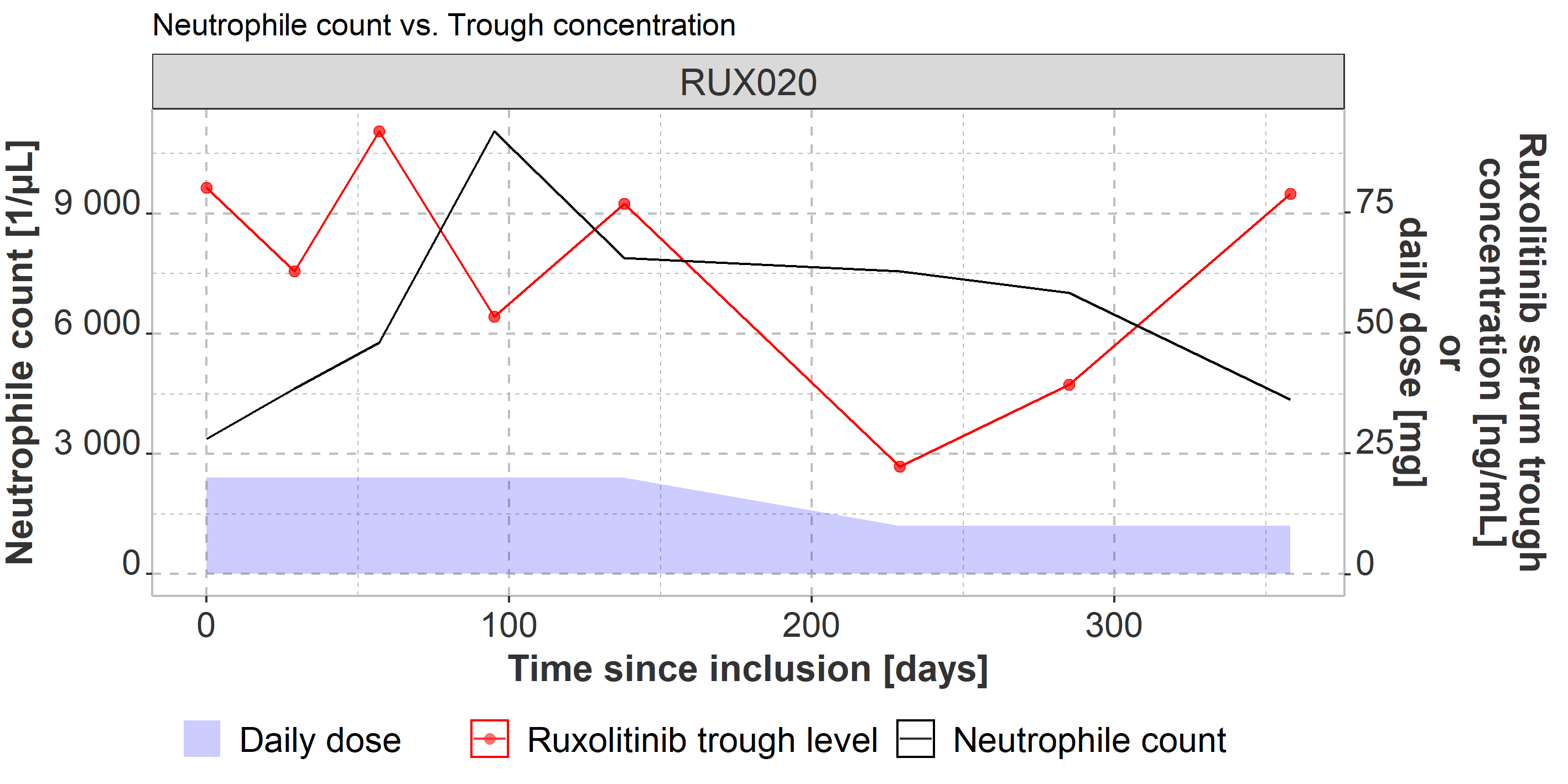 |
| 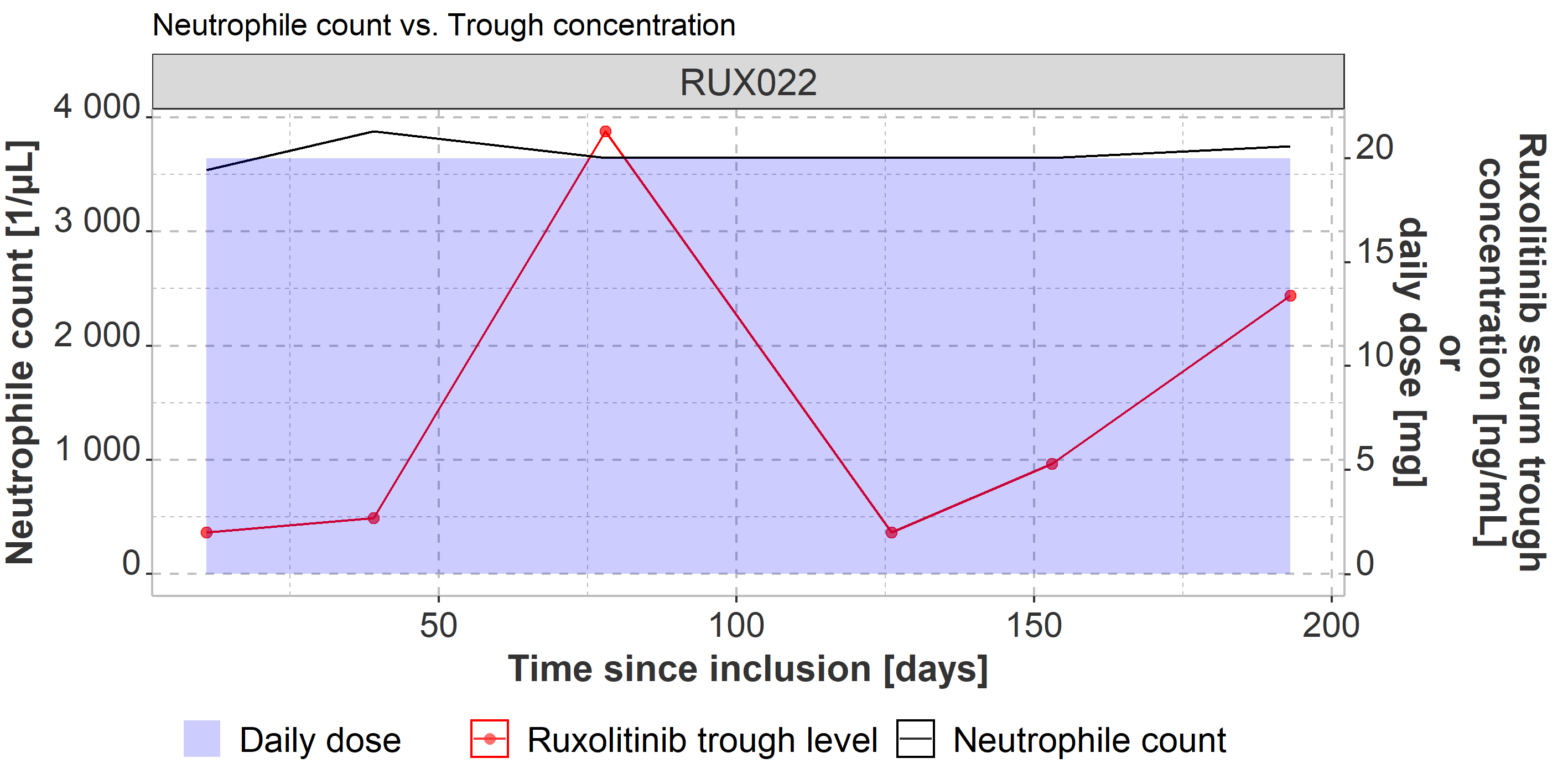 | 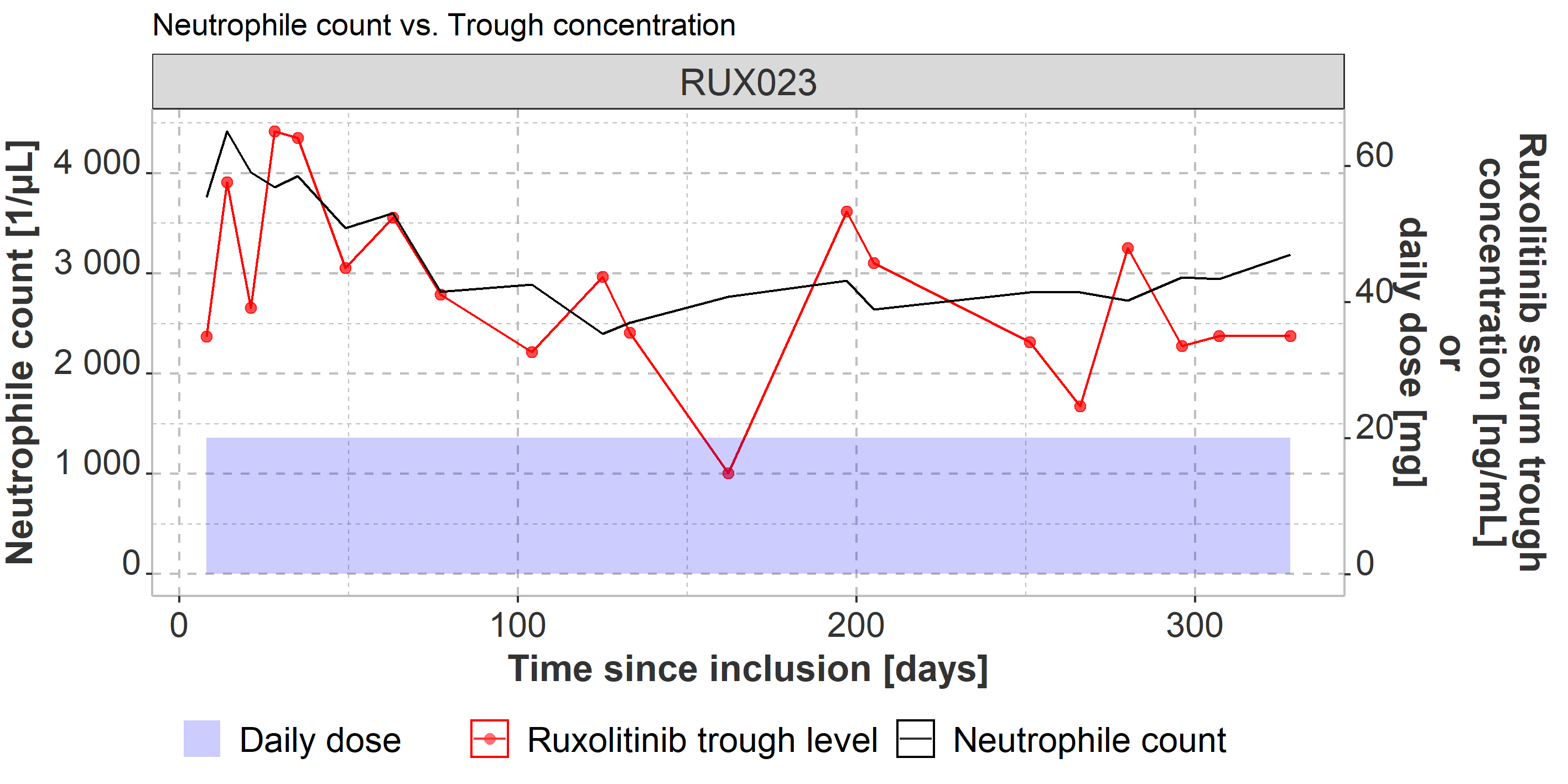 |
| 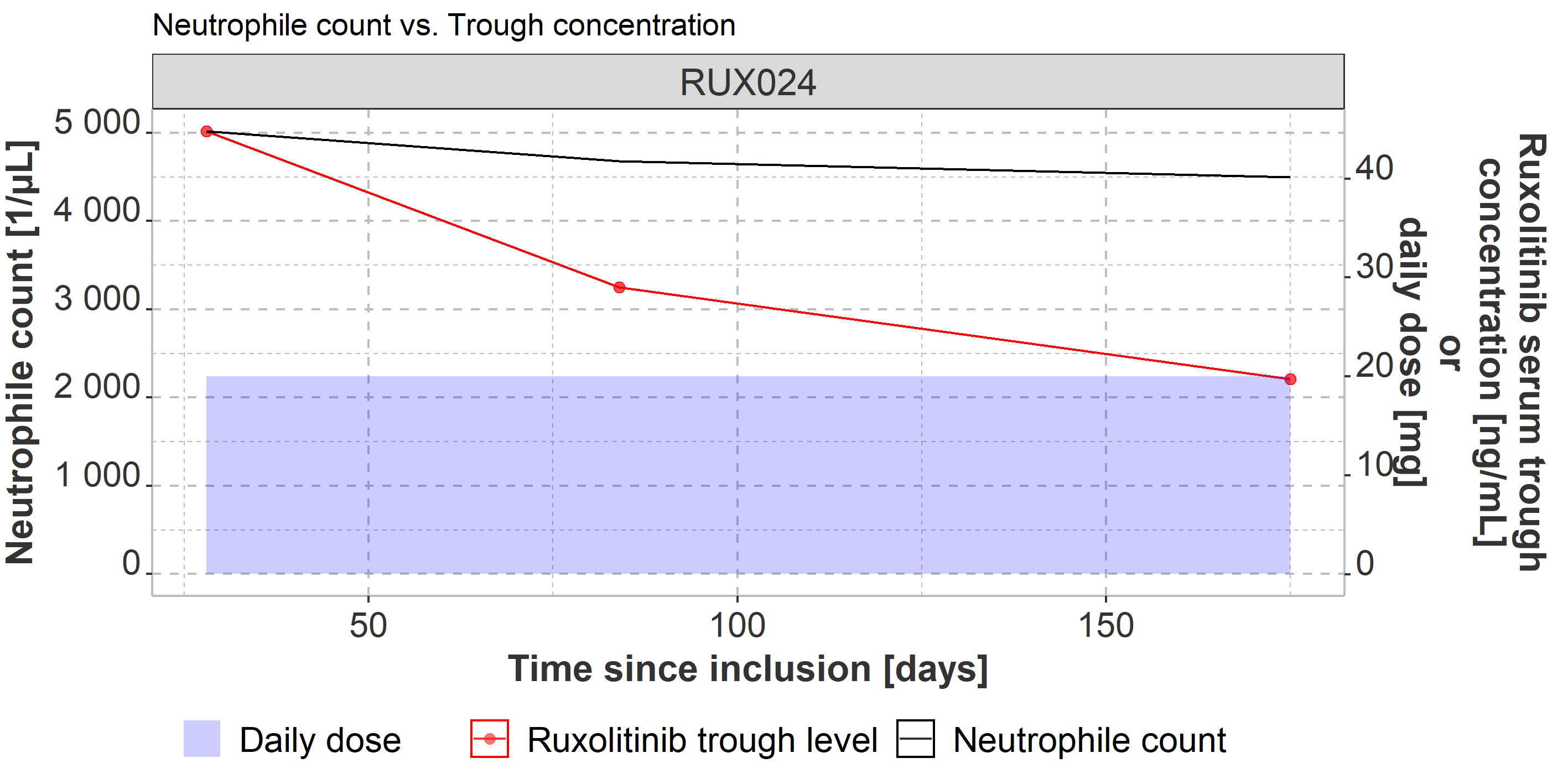 | 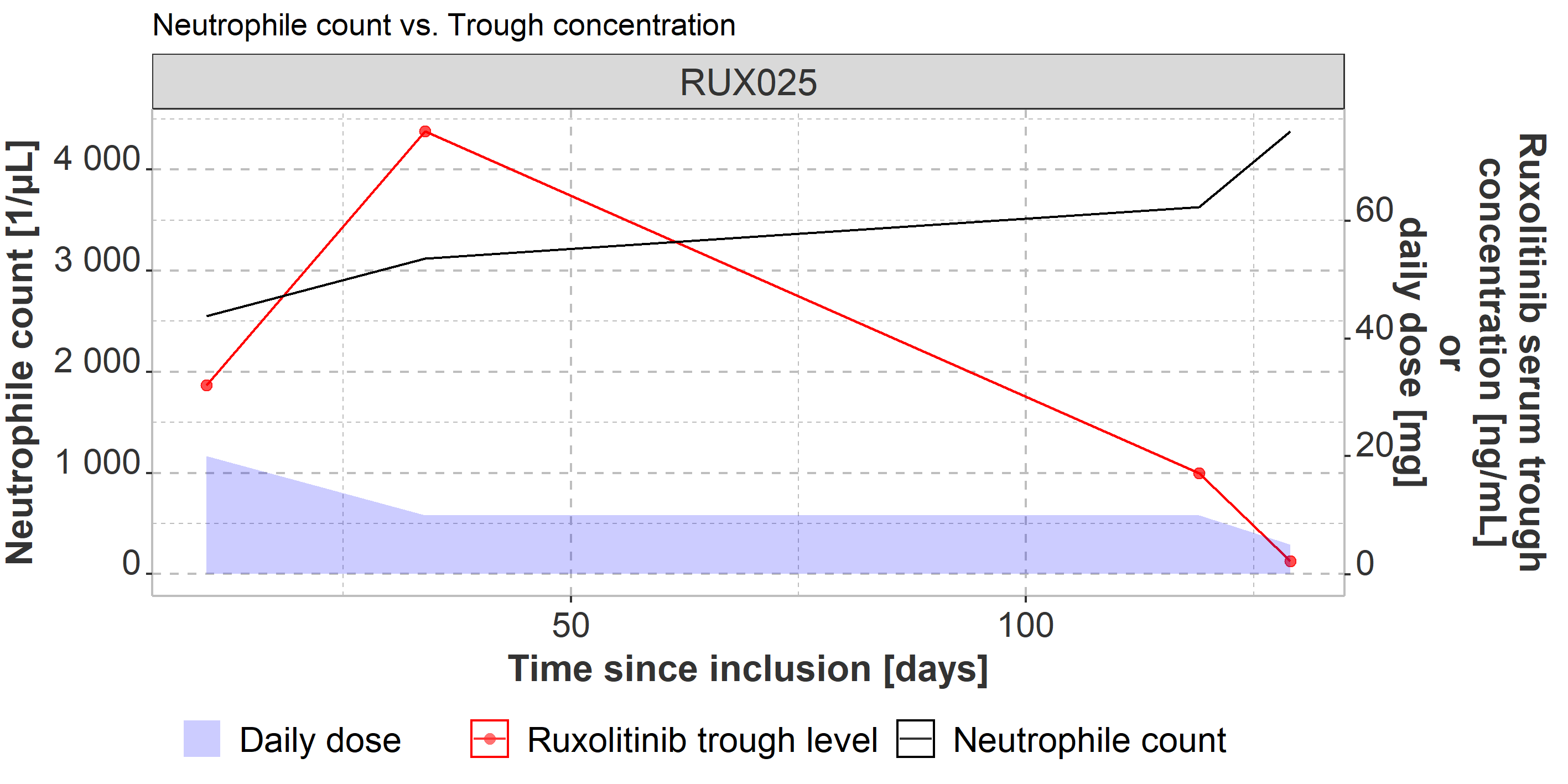 |
| 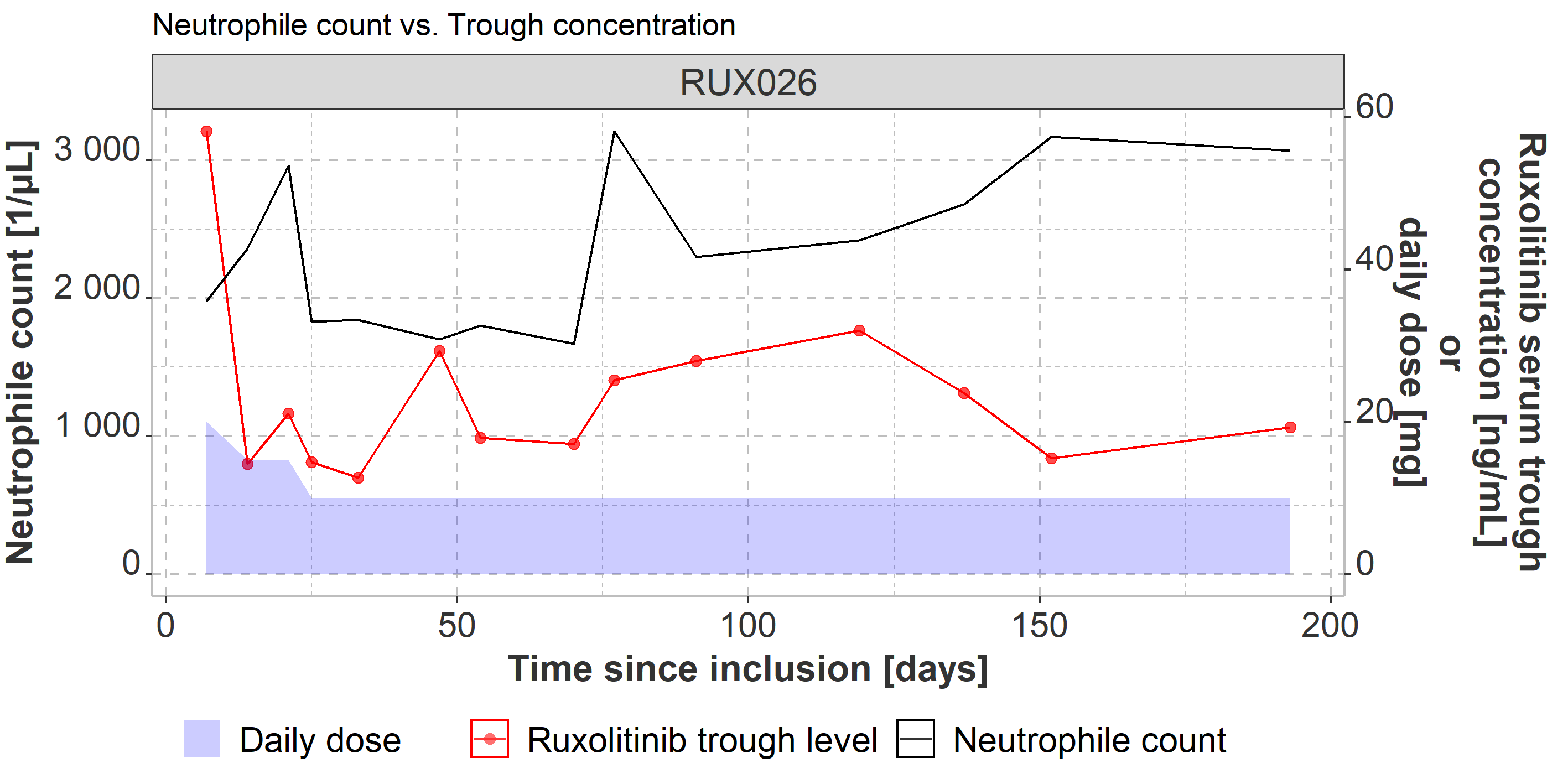 | 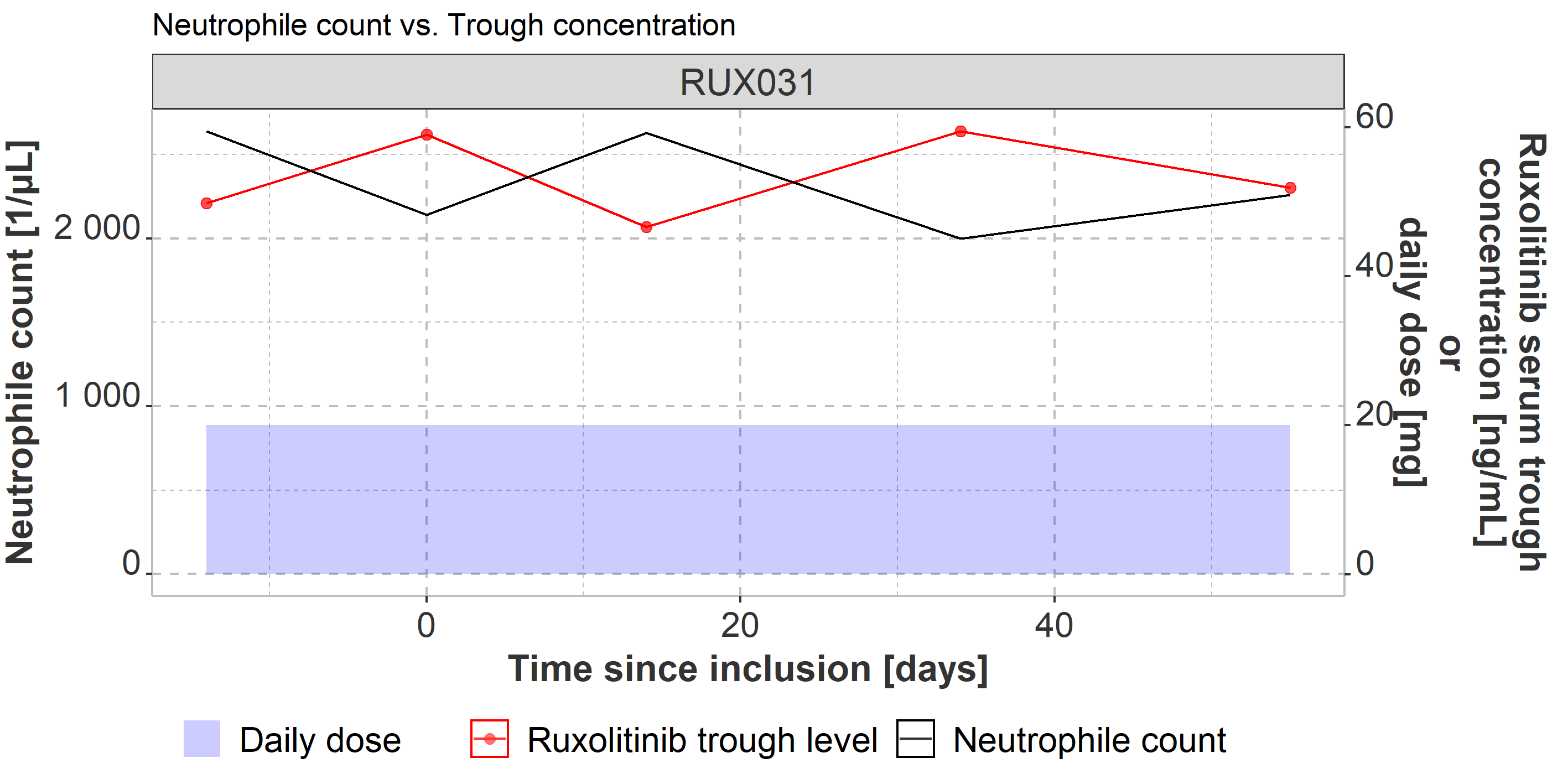 |

| **Supplementary Fig. 8:** Total bilirubin vs. Ruxolitinib trough serum concentration | |
| --- | --- |
| 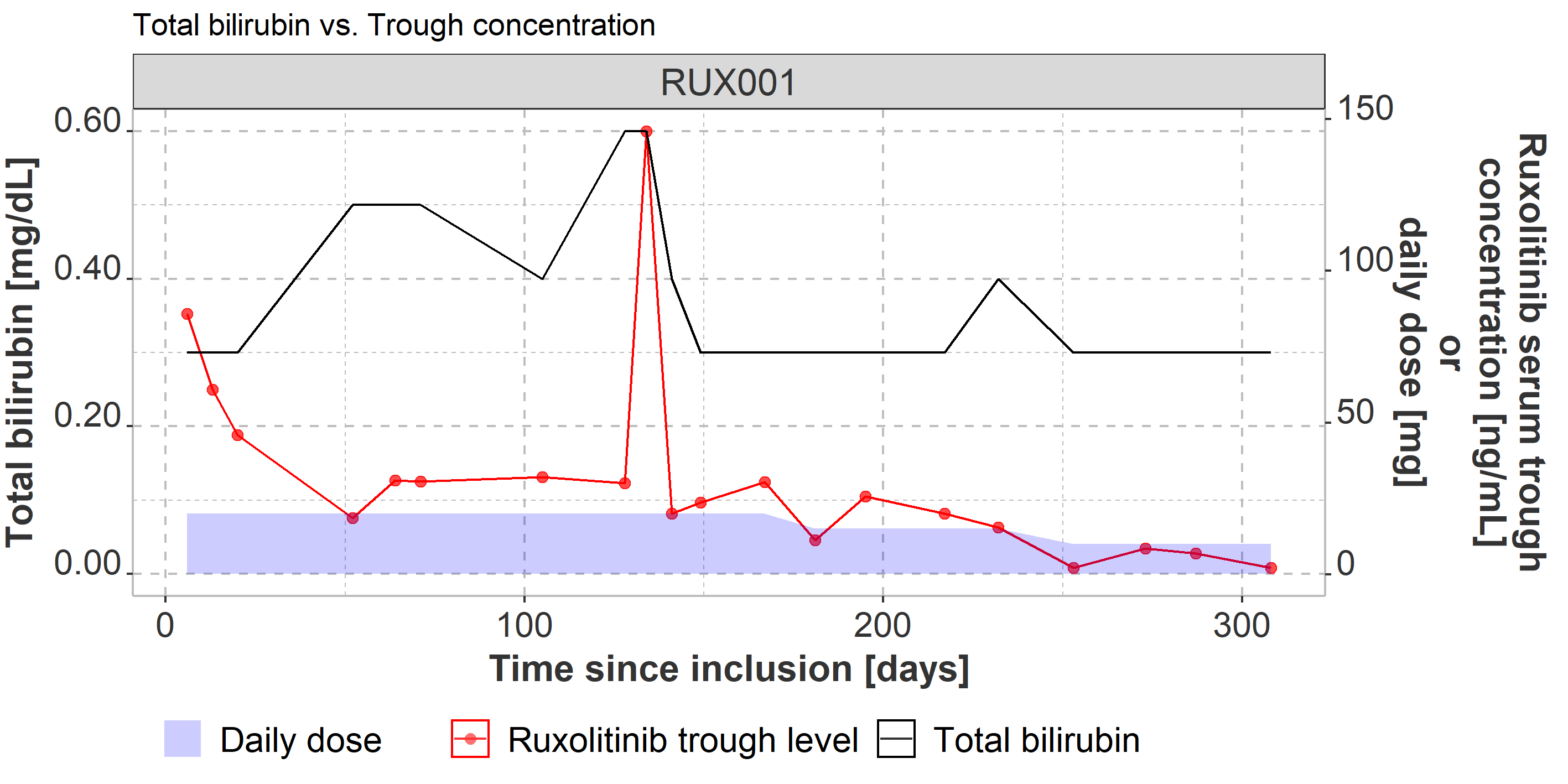 | 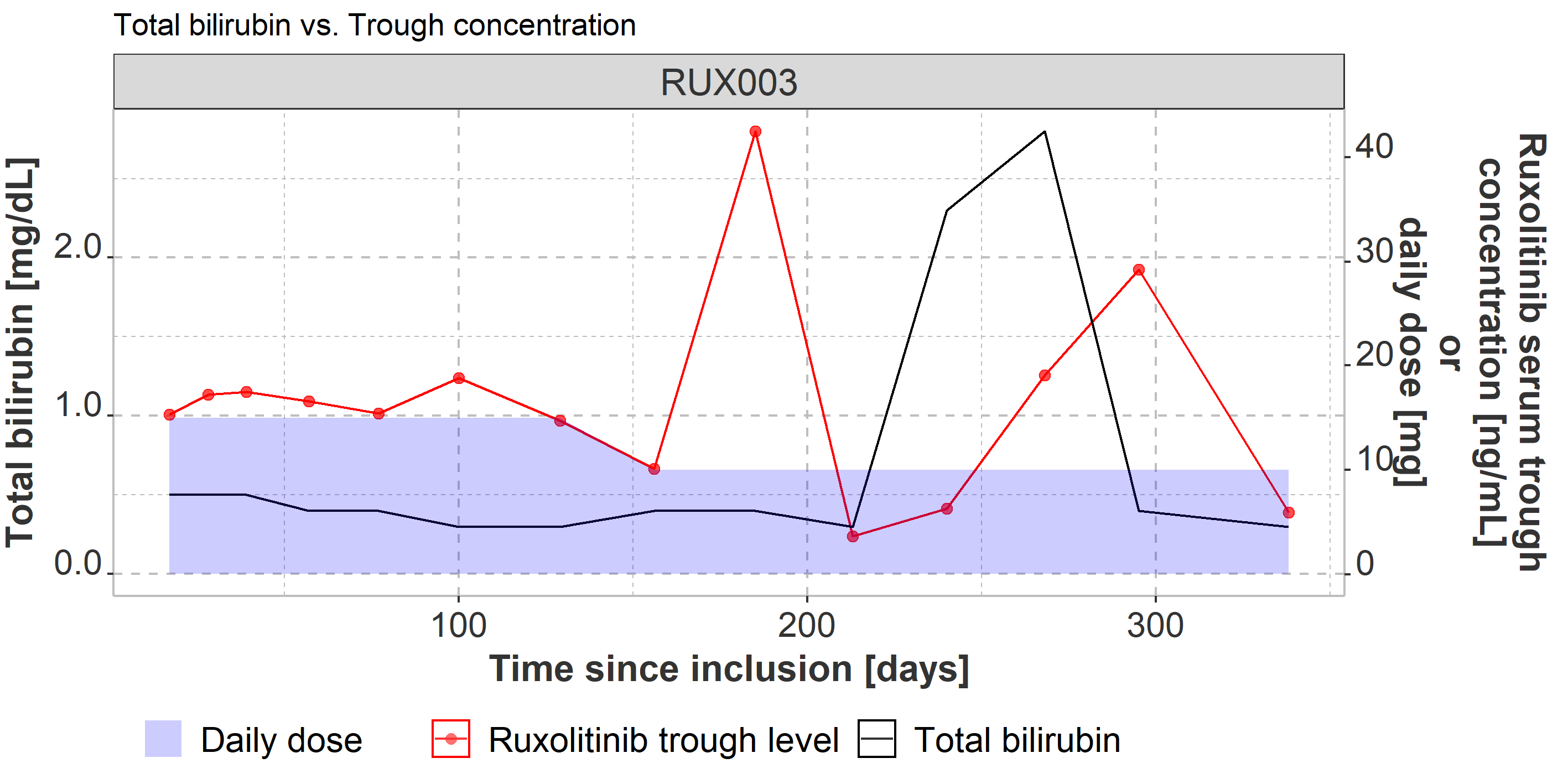 |
| 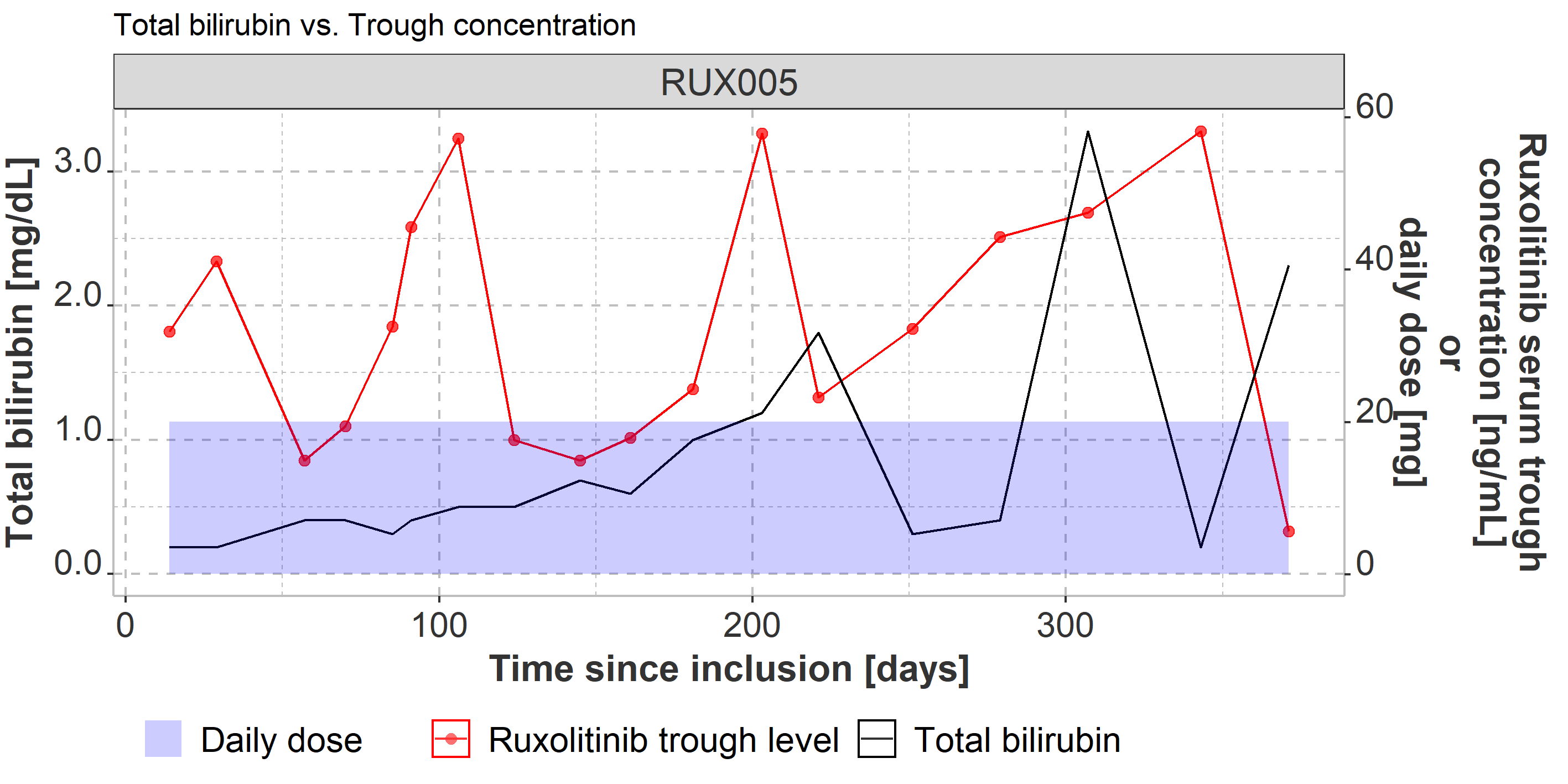 | 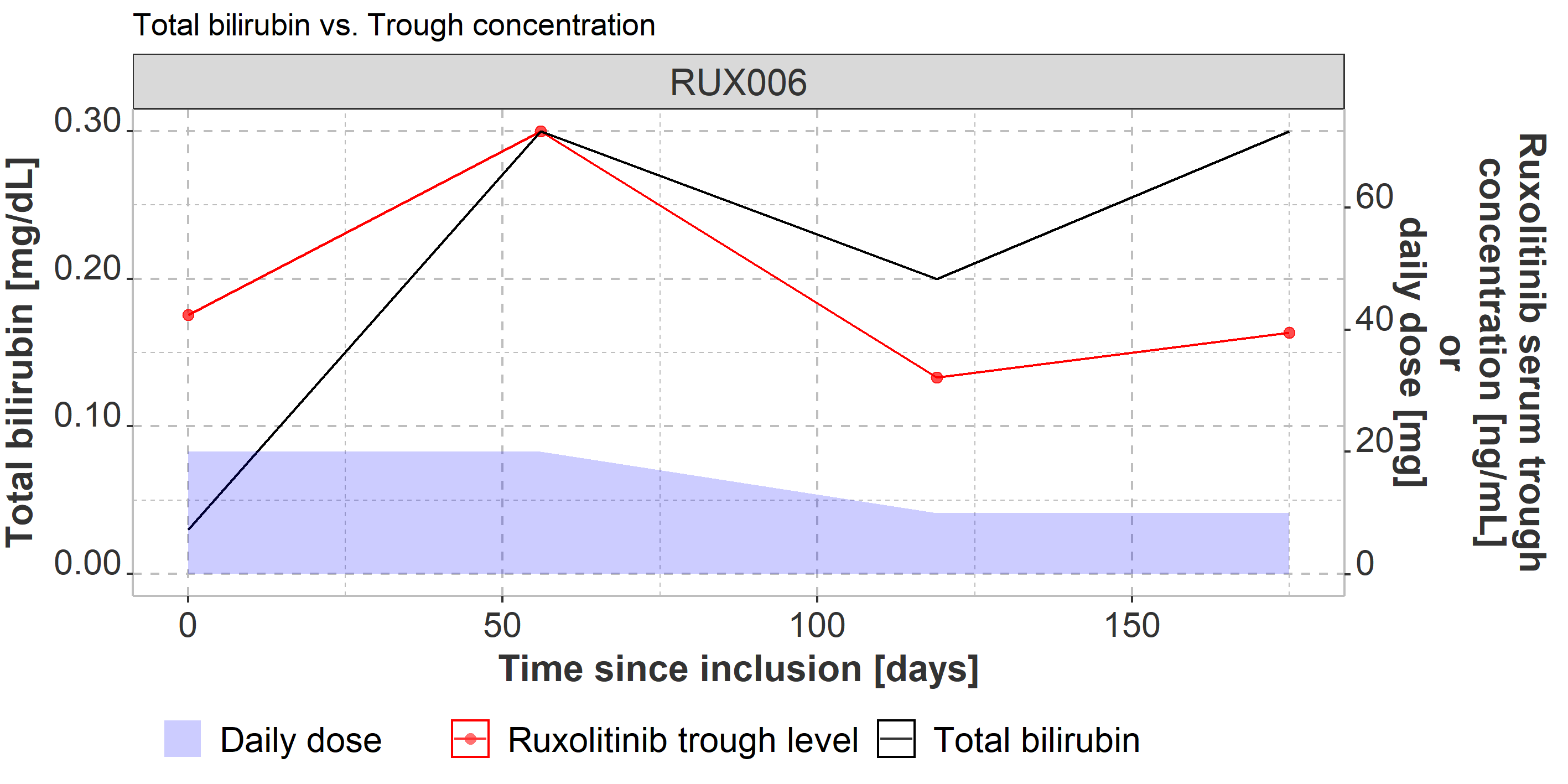 |
| 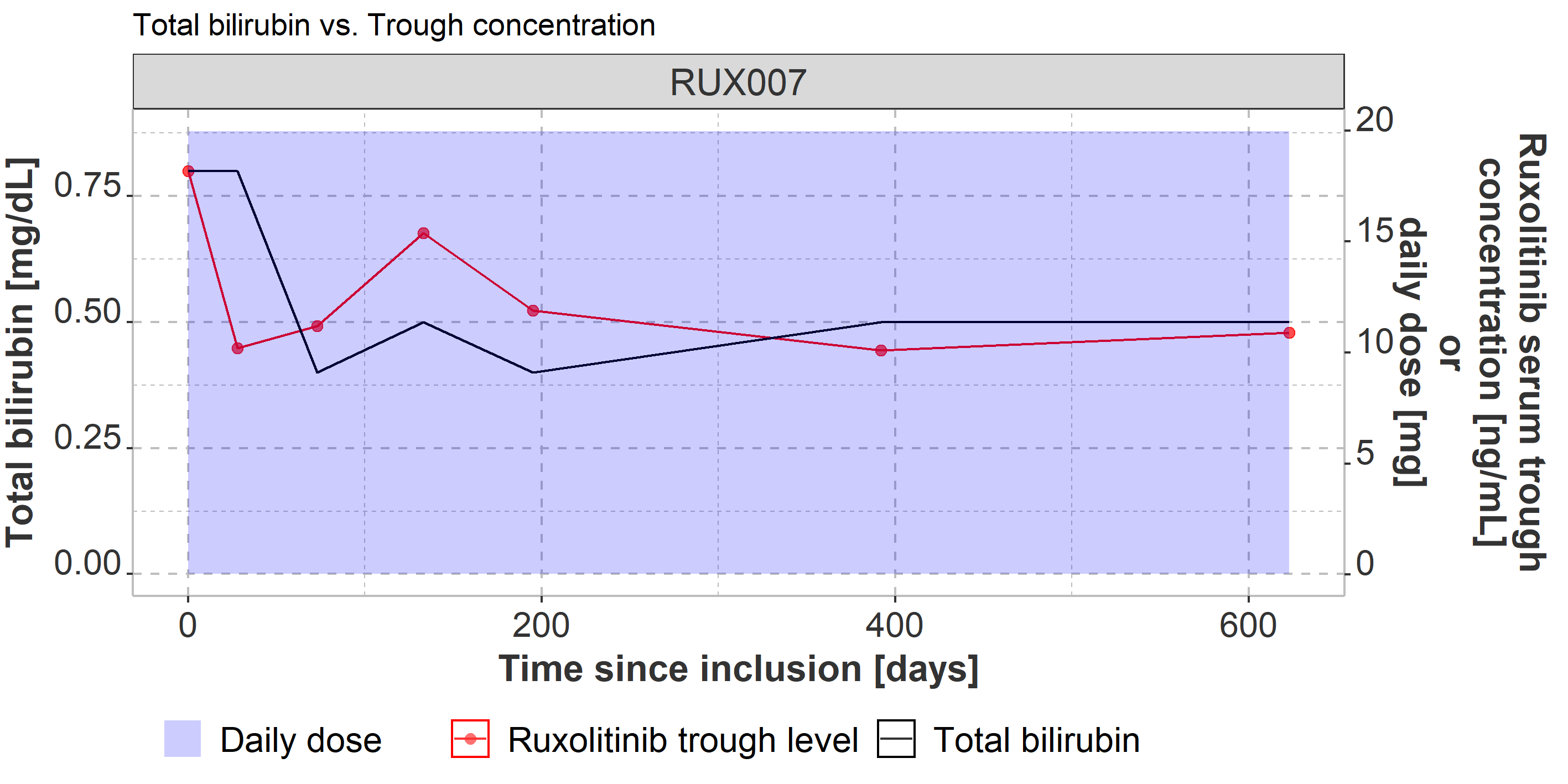 | 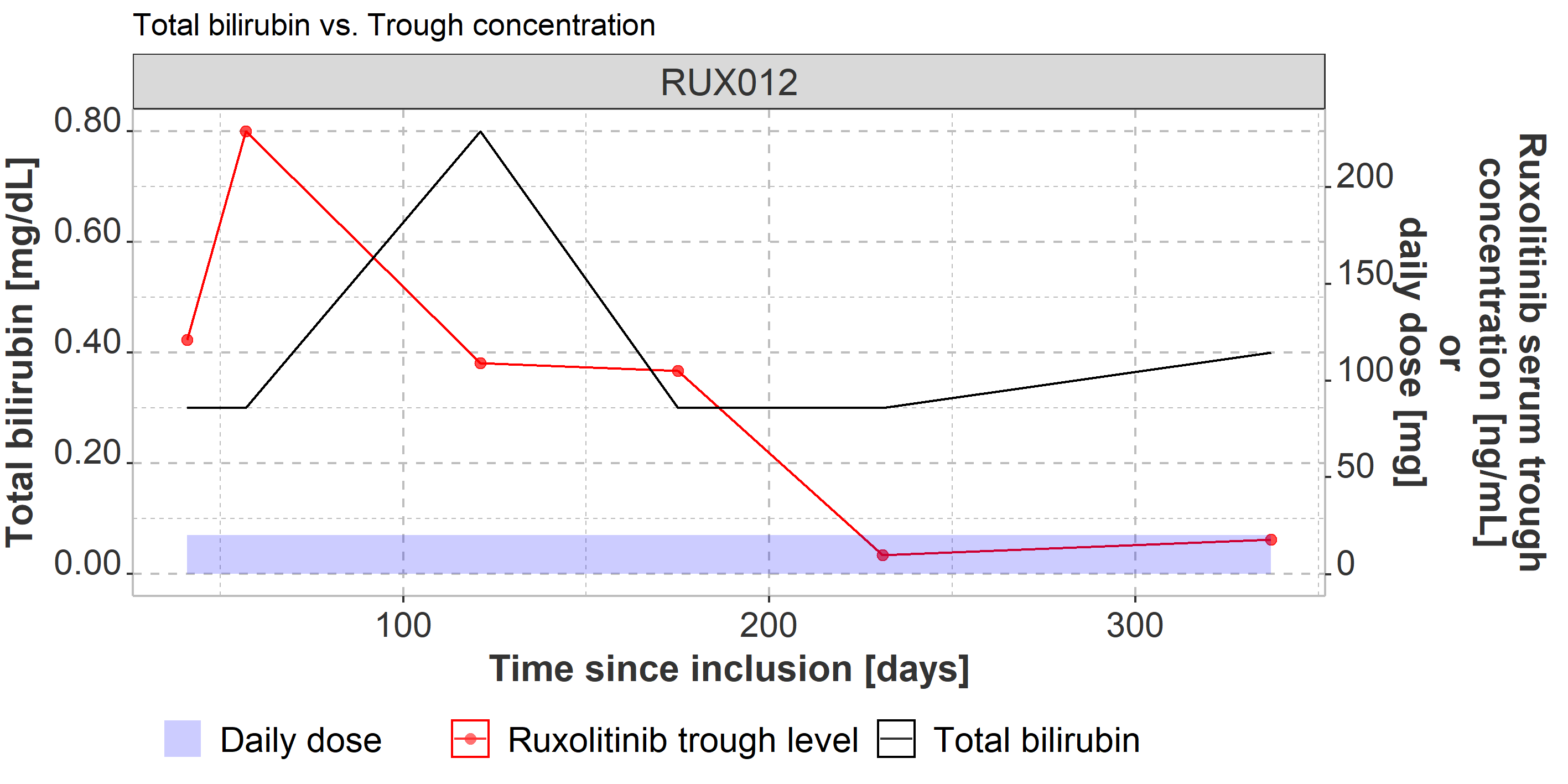 |
| 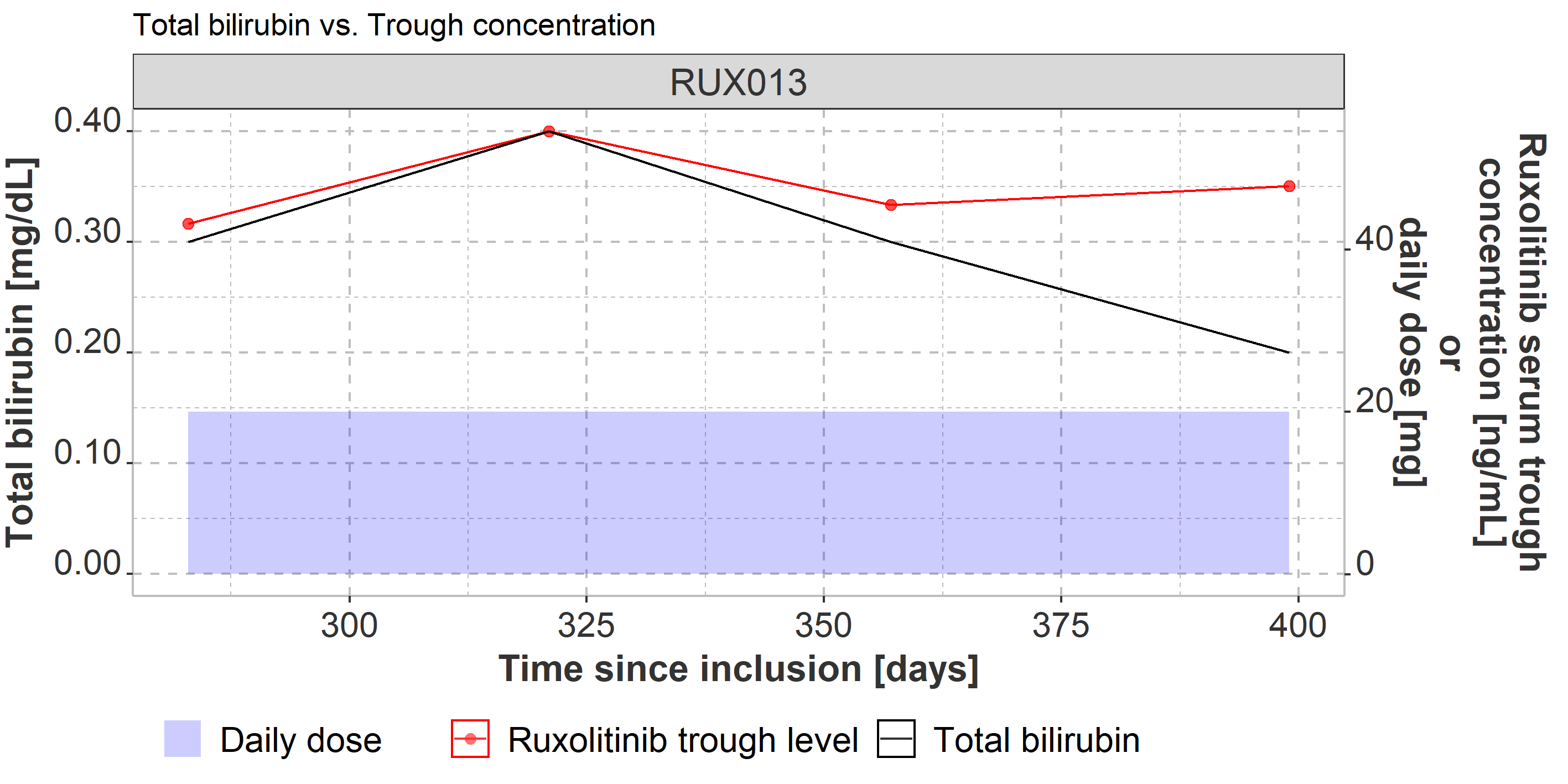 | 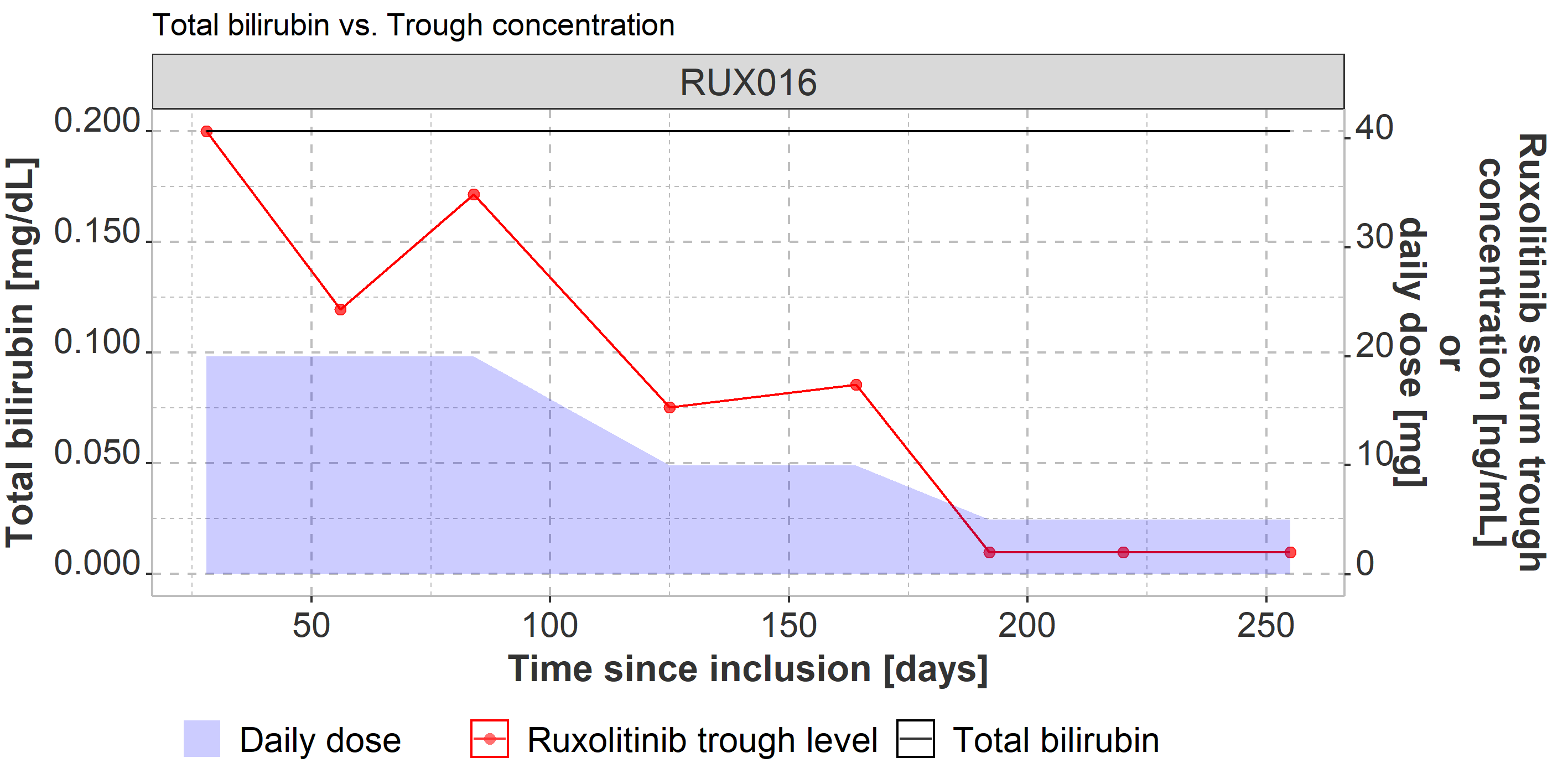 |
| 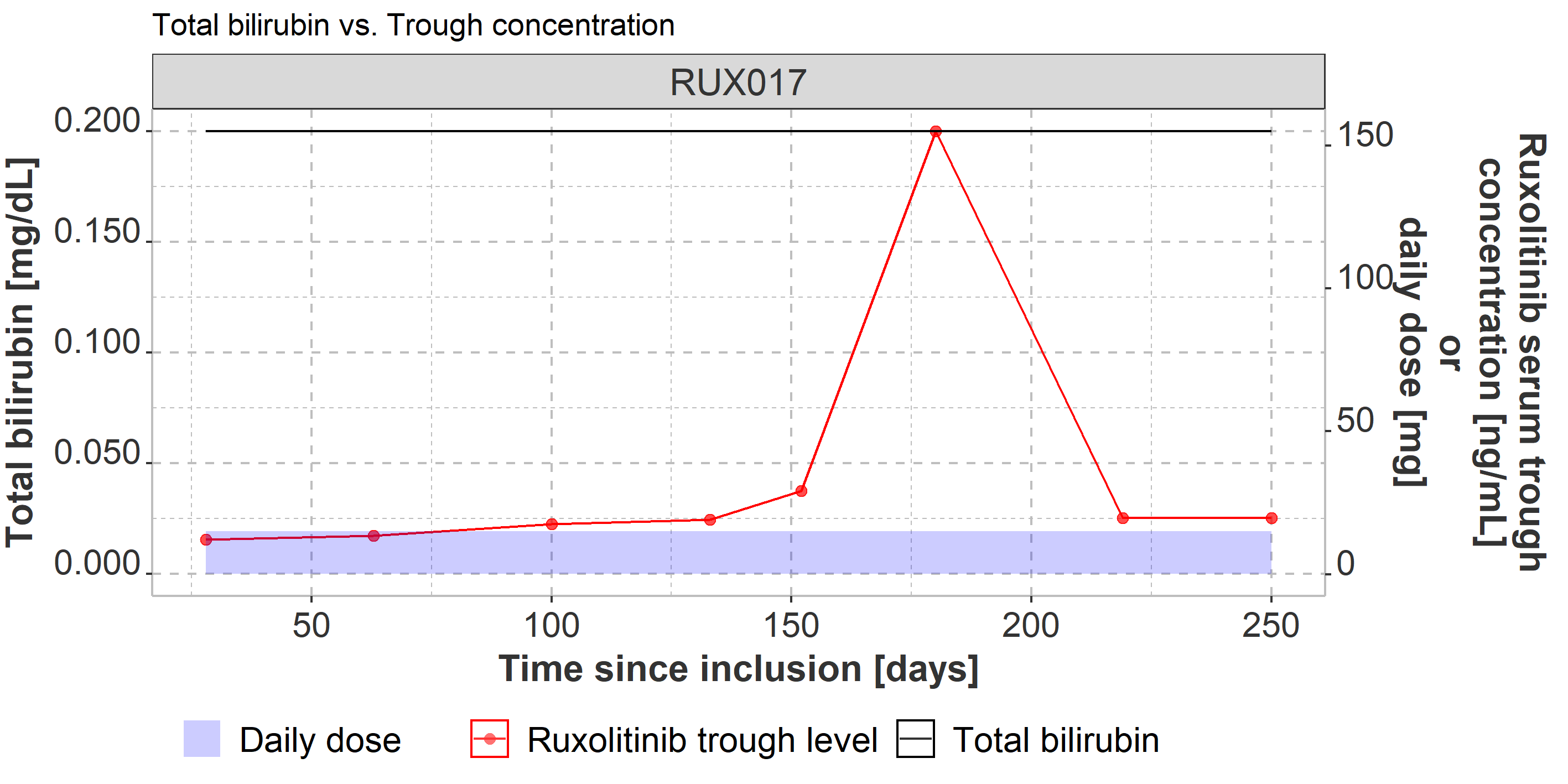 | 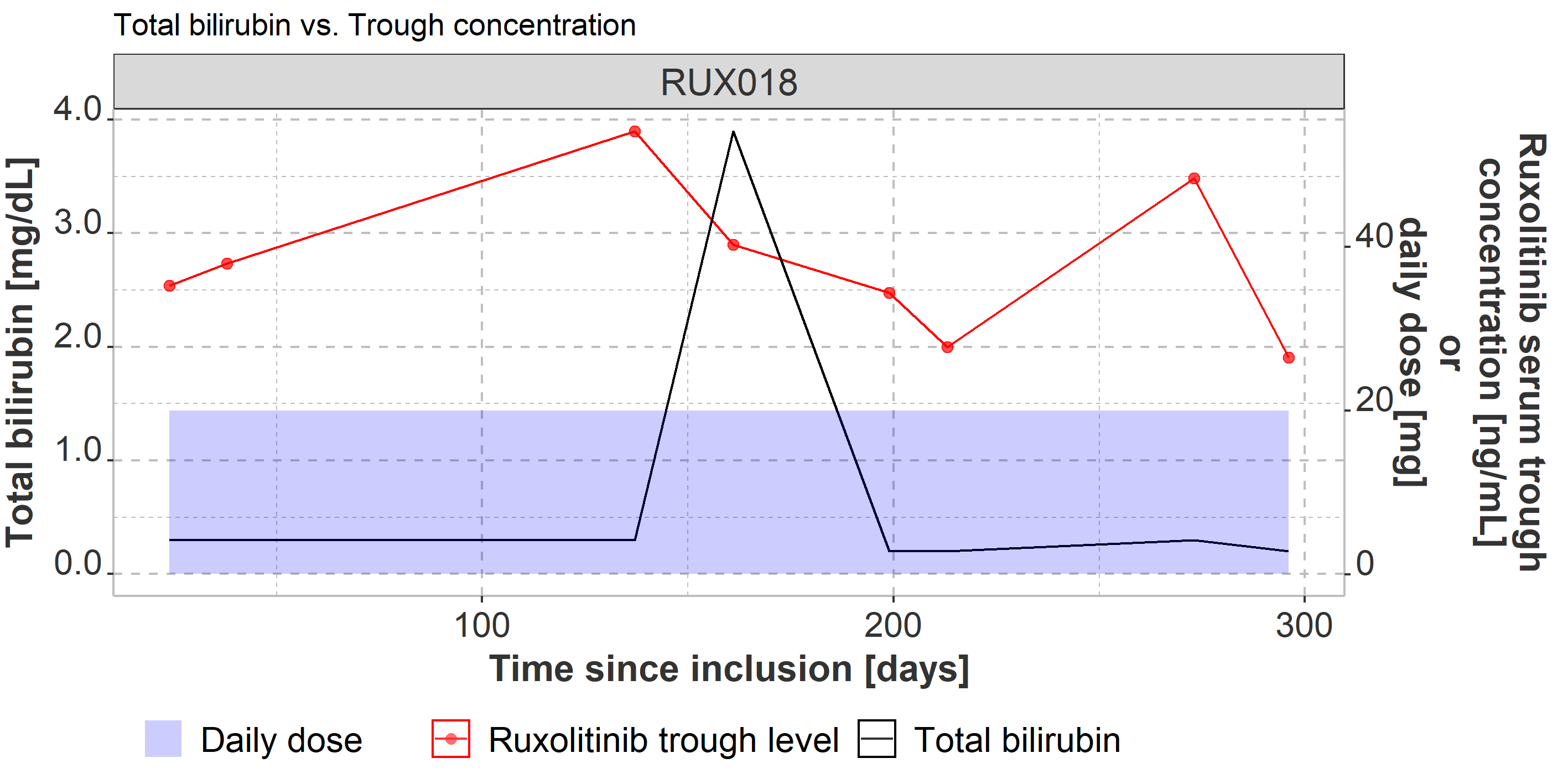 |
| 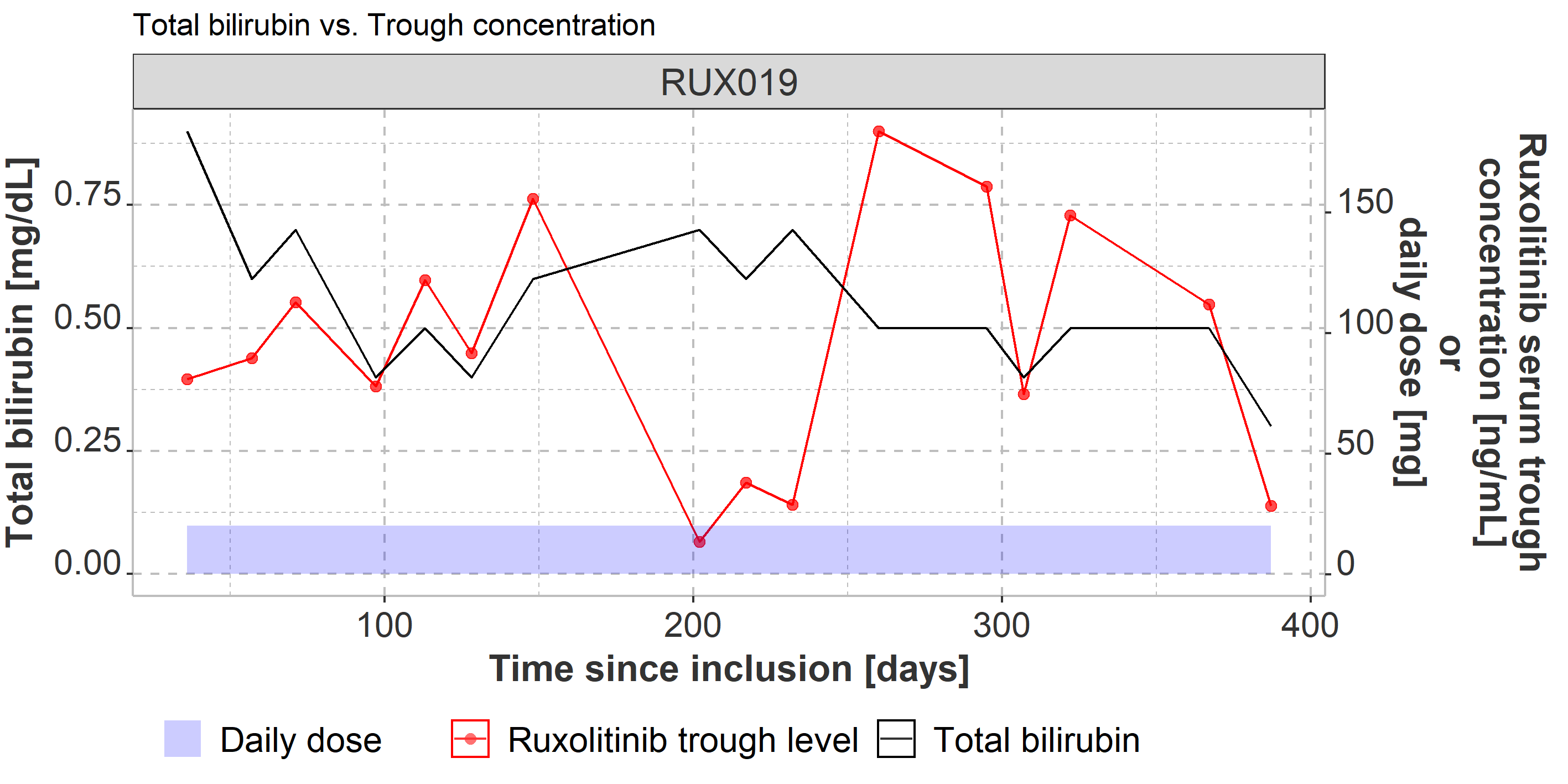 | 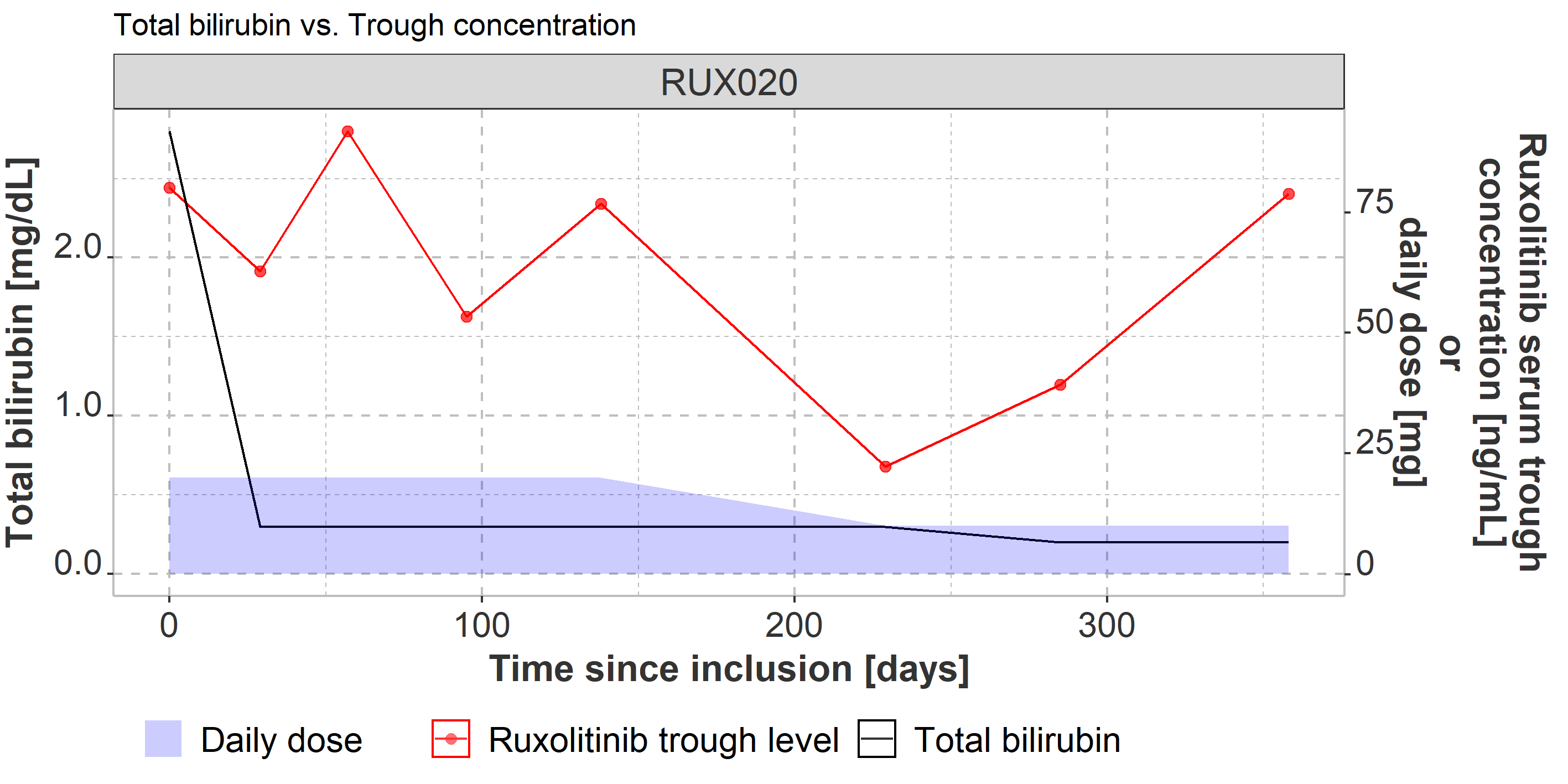 |
| 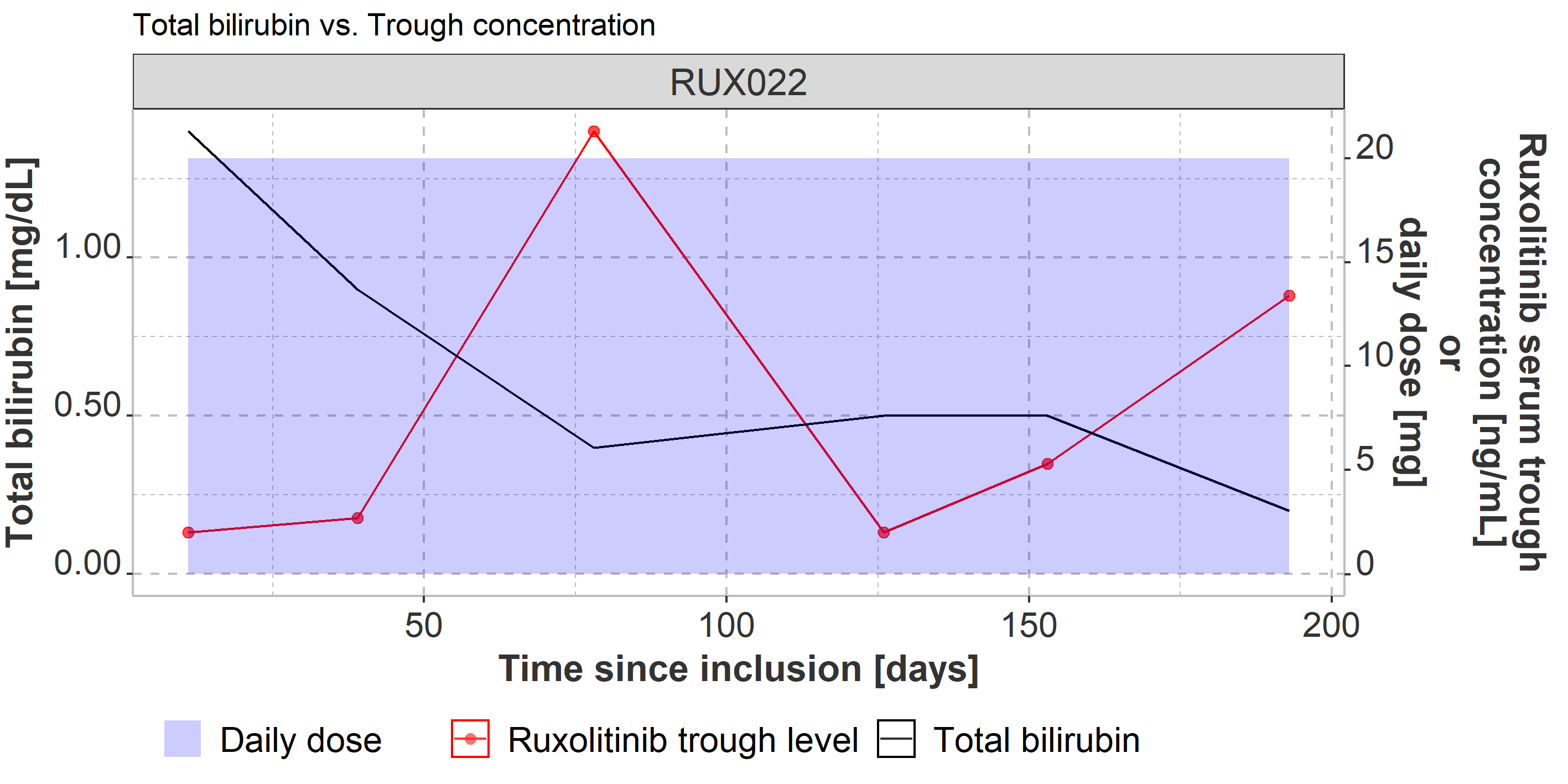 | 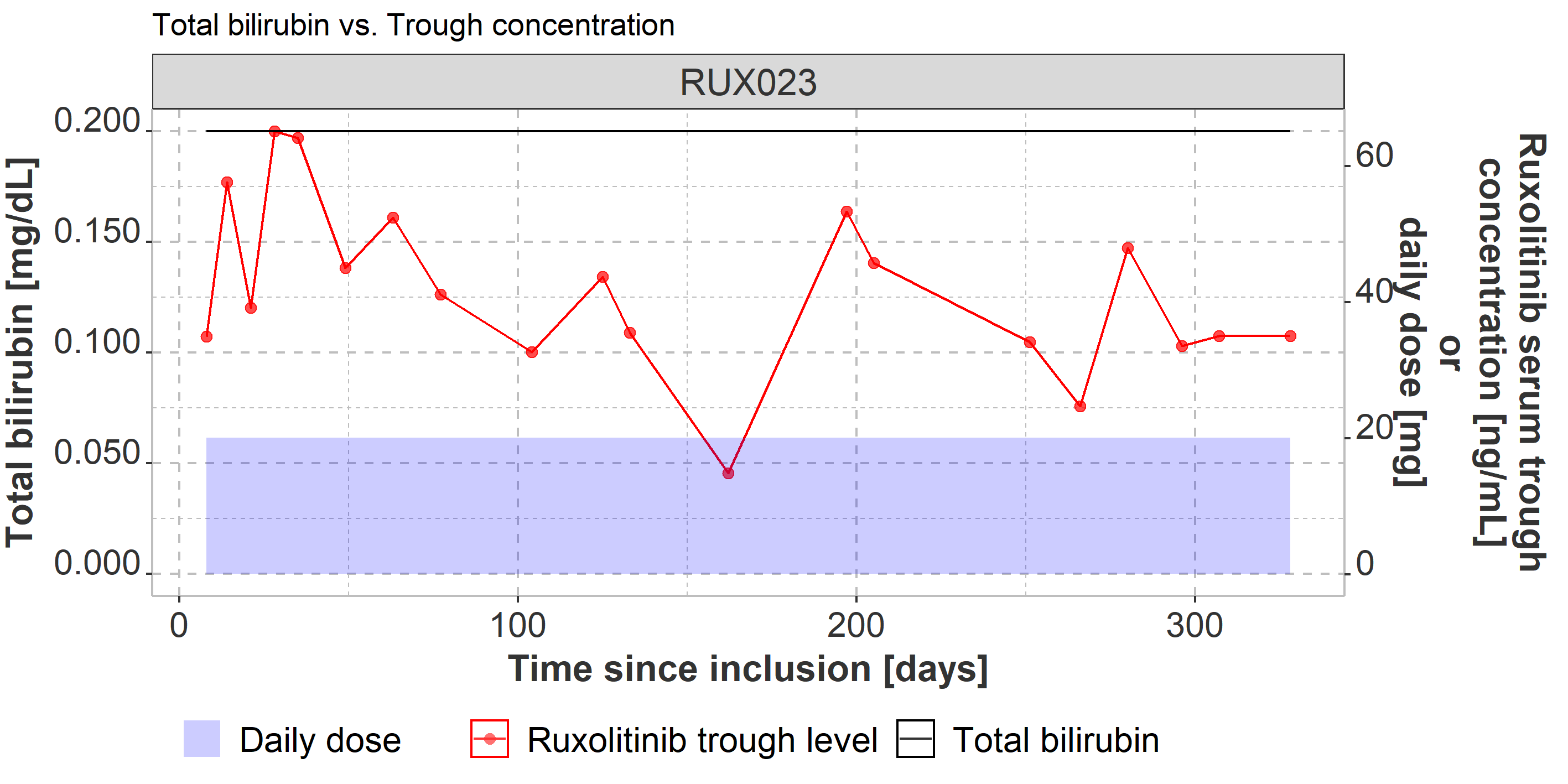 |
| 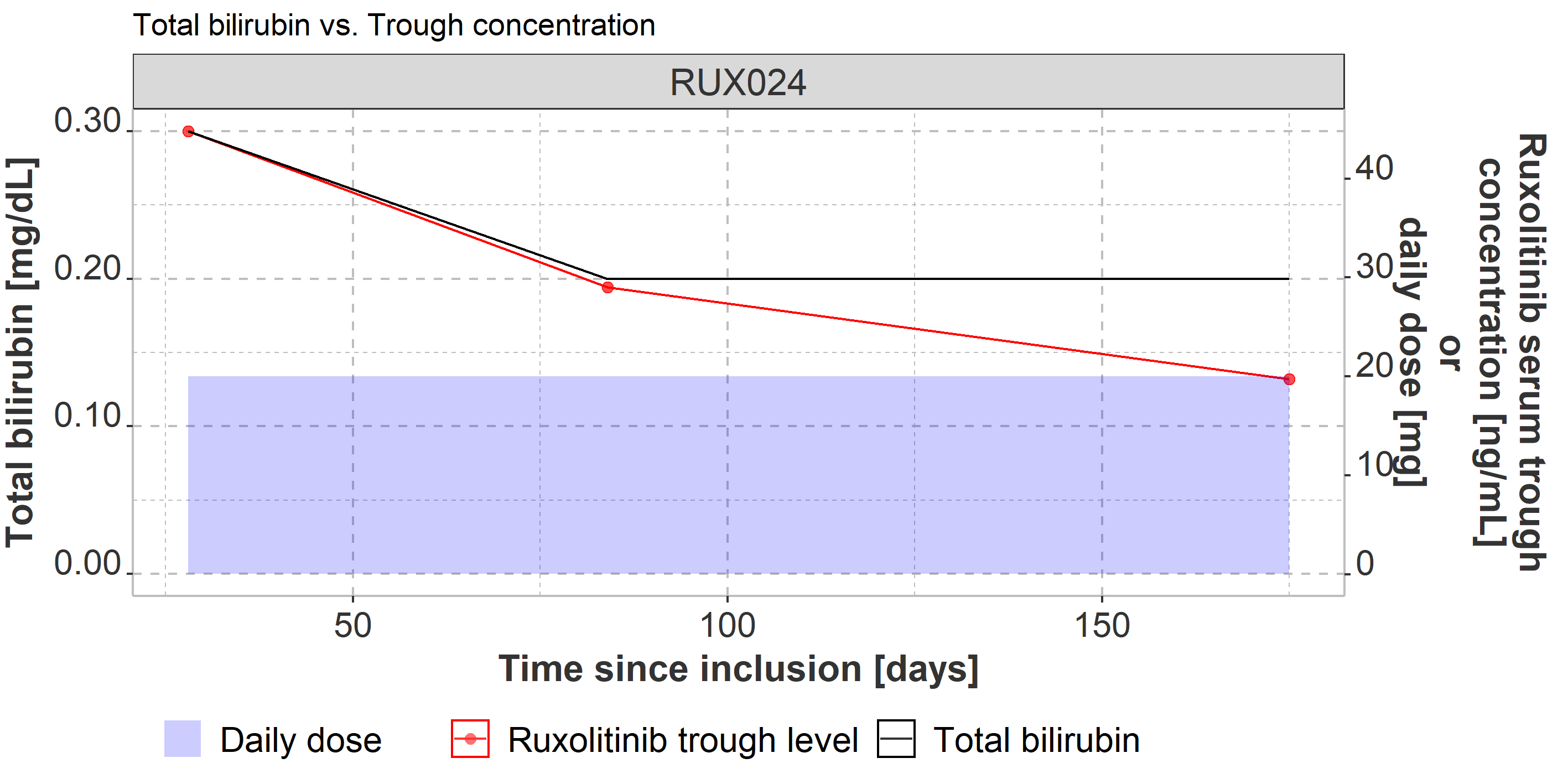 | 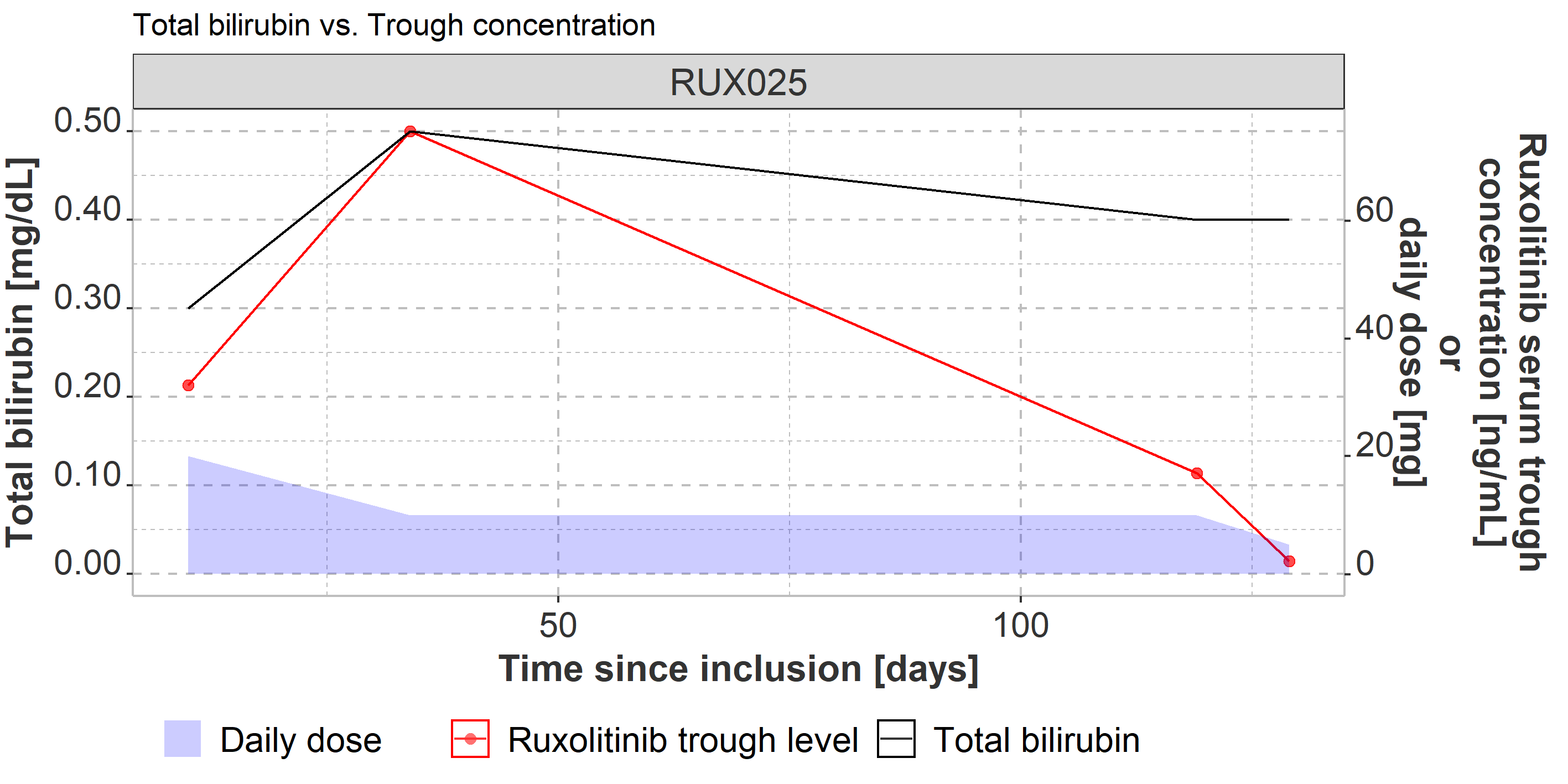 |
| 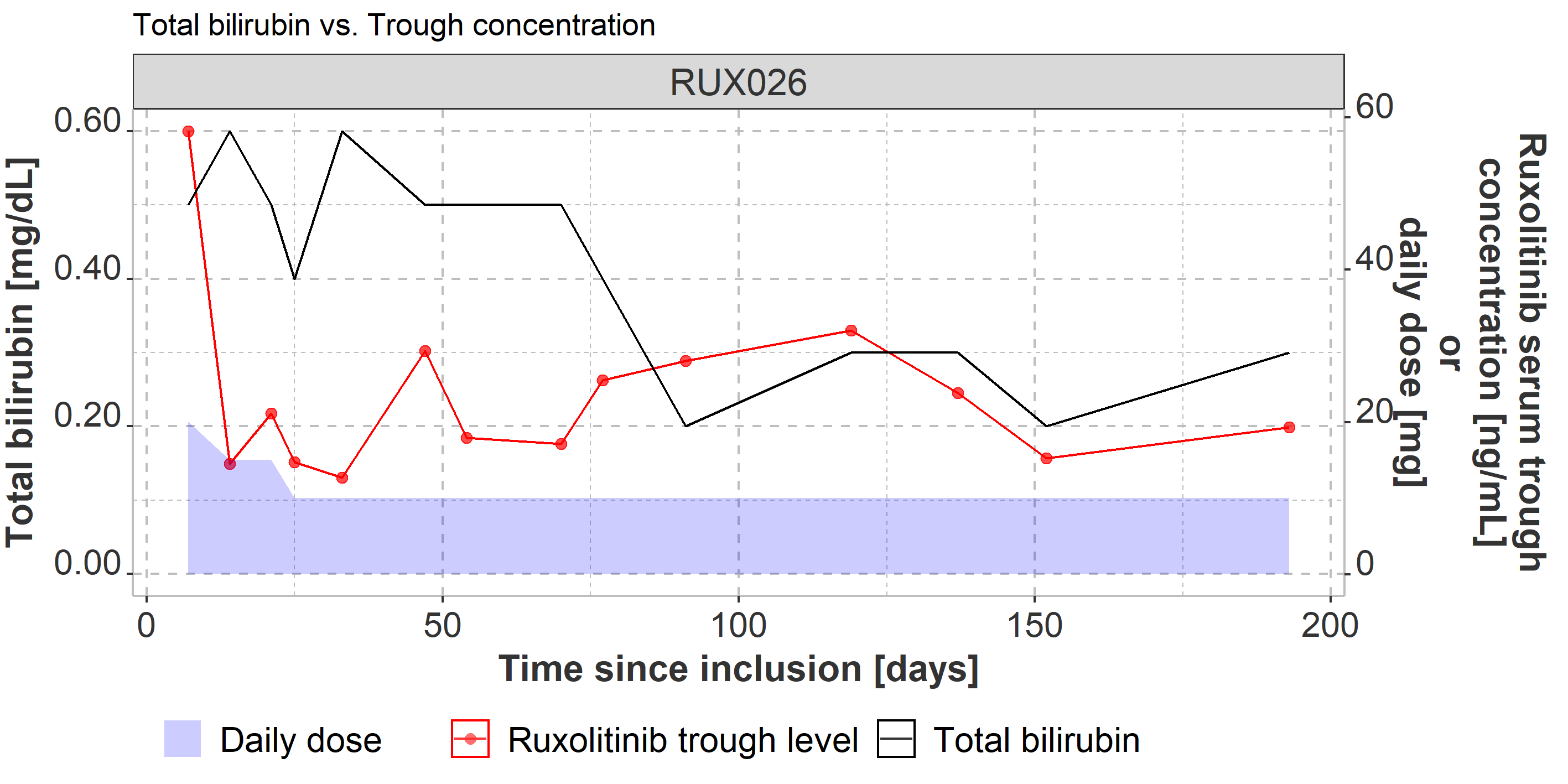 | 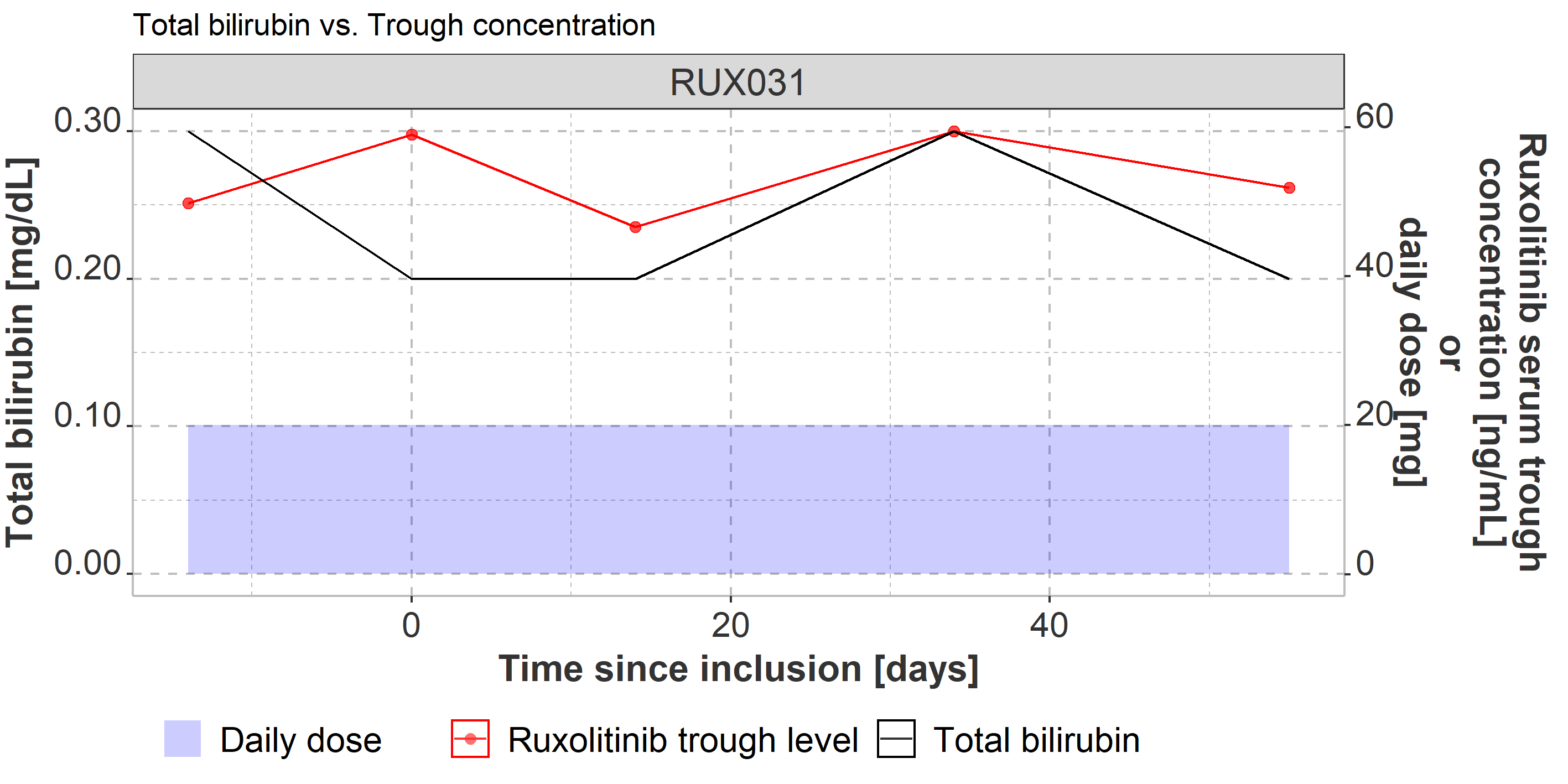 |

**
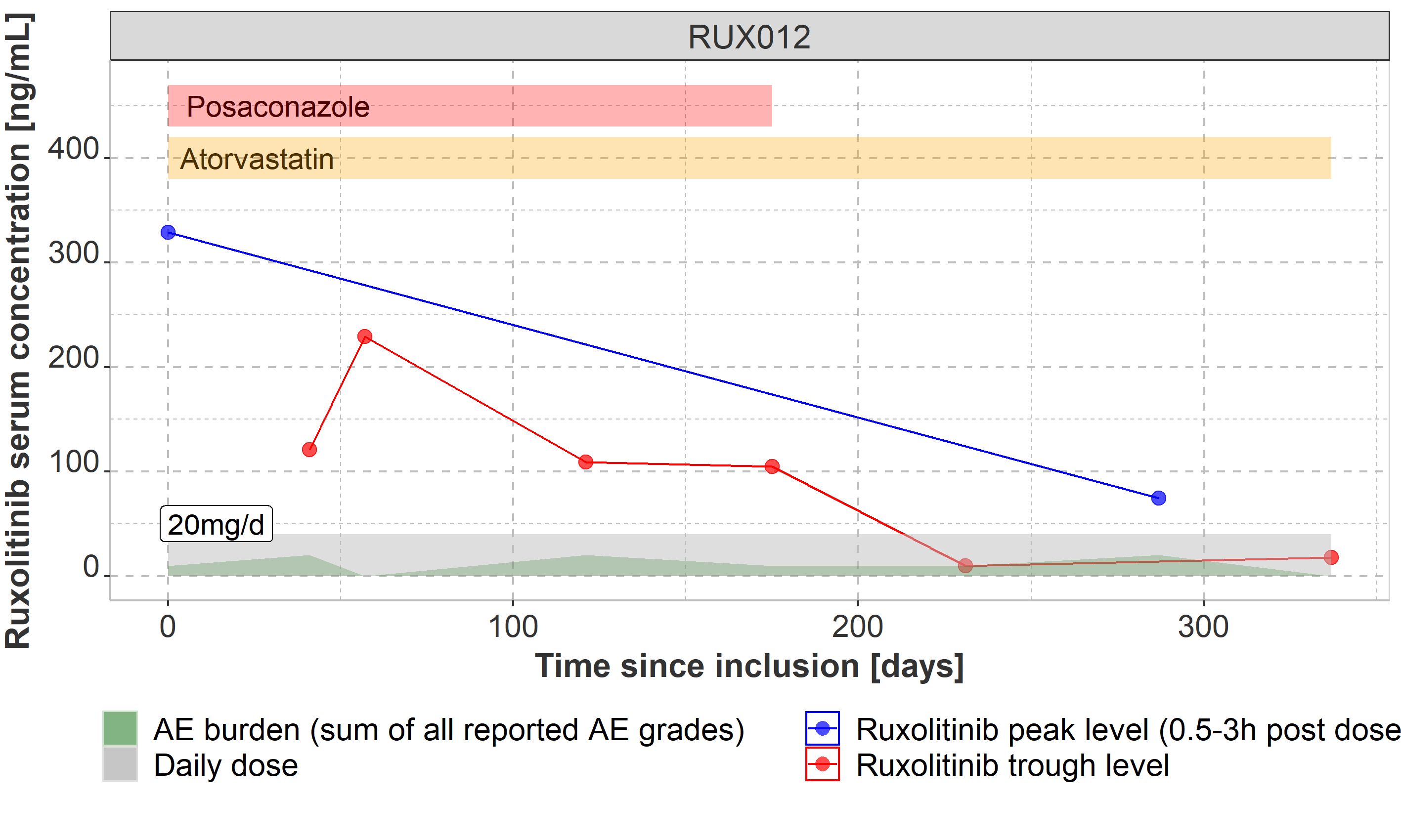

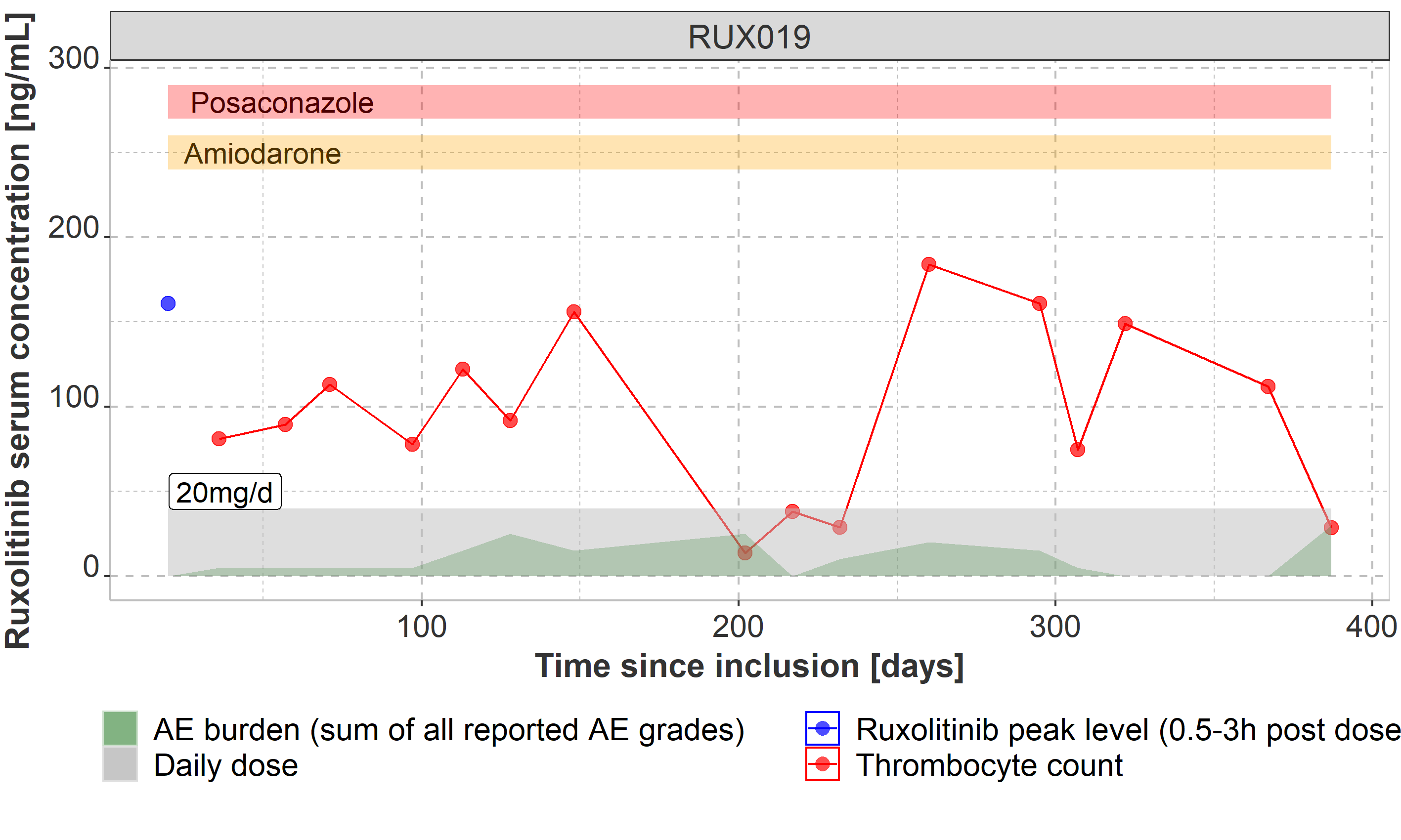
**

**Supplementary Figure 9:** Examples of two patients receiving two CYP inhibitors at the same time. Both patients show extremely high (mostly > 80 ng/mL) trough concentrations under dual inhibition. RUX012 experienced a drastic drop in Ruxolitinib trough serum concentrations after discontinuation of Posaconazole.
